# TNF drives aberrant BMP signaling to induce endothelial and mesenchymal dysregulation in pulmonary hypertension

**DOI:** 10.1172/jci.insight.174456

**Published:** 2025-06-26

**Authors:** Maria de la Luz Garcia-Hernandez, Javier Rangel-Moreno, Qingfu Xu, YeJin Jeong, Soumyaroop Bhattacharya, Ravi Misra, Stacey Duemmel, Ke Yuan, Benjamin D. Korman

**Affiliations:** 1 Division of Allergy, Immunology, and Rheumatology, Department of Medicine, and; 2 Department of Pediatrics, University of Rochester Medical Center, Rochester, New York, USA.; 3 Division of Pulmonary Medicine, Boston Children’s Hospital, Boston, Massachusetts, USA.

**Keywords:** Inflammation, Pulmonology, Vascular biology, Bioinformatics, Endothelial cells, Molecular pathology

## Abstract

The pathobiology of pulmonary hypertension (PH) is complex and multiple cell types contribute to disease pathogenesis. We sought to characterize the molecular crosstalk between endothelial and mesenchymal cells that promote PH in the tumor necrosis factor α–transgenic (TNF-Tg) model of PH. Pulmonary endothelial and mesenchymal cells were isolated from WT and TNF-Tg mice and underwent single-cell RNA sequencing. Data were analyzed using clustering, differential gene expression and pathway analysis, ligand-receptor interaction, transcription factor binding, and RNA velocity assessments. Significantly altered ligand-receptor interactions were confirmed with immunofluorescent staining. TNF-Tg mice had increases in smooth muscle cells and Col14^+^ fibroblasts, and reductions in general capillary (gCAP) endothelial cells, Col13^+^ fibroblasts, pericytes, and myofibroblasts. Pathway analysis demonstrated NF-κB–, JAK/STAT-, and interferon-mediated inflammation, endothelial apoptosis, loss of vasodilatory pathways, increased TGF-β signaling, and smooth muscle cell proliferation. Ligand-receptor analysis demonstrated a loss of BMPR2 signaling in TNF-Tg lungs and establishment of a maladaptive BMP signaling cascade, which functional studies revealed stemmed from endothelial NF-κB activation and subsequent endothelial SMAD2/3 signaling. This system highlights a complex set of changes in cellular composition, cell communication, and cell fate driven by TNF signaling that lead to aberrant BMP signaling that is critical for development of PH.

## Abstract 2

Molecular characterization of the TNF-Tg model of pulmonary hypertension recapitulates many features of human pulmonary hypertension and shows a striking alteration in BMP signaling

## Graphical abstract

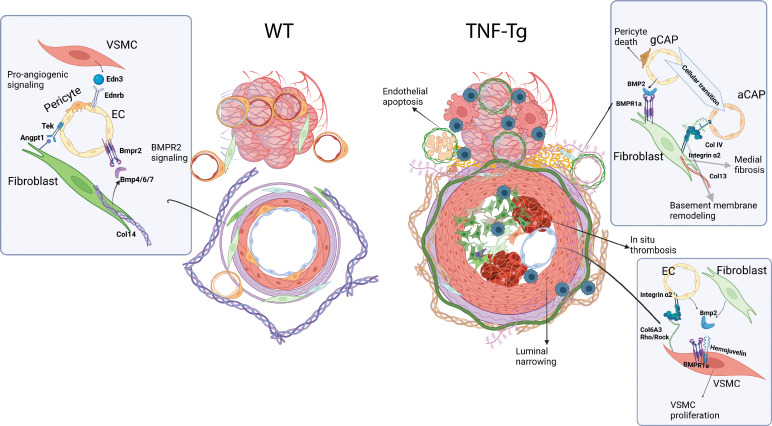

## Introduction

Pulmonary hypertension (PH) is a severe vasculopathy that leads to remodeling of the pulmonary circulation and ultimately causes right ventricular (RV) failure and death. Despite recent advances in therapy, patients with PH still manifest unacceptably high rates of morbidity and mortality. Although the pathophysiology of PH is complex, it is well established that endothelial apoptosis is involved in intimal change, smooth muscle cell hyperplasia contributes to medial thickening, and infiltration of inflammatory cells is critical for adventitial remodeling (1). Multiple pathway dysregulations occur in PH, including anomalies in BMP, Notch, TGF-β, VEGF, nitric oxide, and mitochondrial metabolism. Of note, BMPR2 signaling seems to be essential in PH pathogenesis, given its mutation association with the majority of genetically heritable pulmonary arterial hypertension (PAH) and changes in all types of PAH even without genetic mutations (1–3).

Animal models of PH have traditionally included the Sugen-hypoxia and monocrotaline models, which recapitulate multiple important aspects of disease pathophysiology, including vessel muscularization, RV hypertrophy, and elevated pulmonary arterial pressures (4). We recently described that tumor necrosis factor α–transgenic (TNF-Tg) mice, a mouse model of inflammatory arthritis, develop a severe obliterative pulmonary vasculopathy, which leads to death by 5–6 months (5). Using genomic assessment, we showed multiple pathway similarities between TNF-Tg mice and patients with connective tissue disease–associated PAH (CTD-PAH) (5), which is the second most common cause of PAH, and a subgroup of patients who have poorer outcomes than those with idiopathic PAH. In addition to characterizing the model and demonstrating substantially elevated pulmonary artery pressures and RV hypertrophy, we performed bone marrow chimera experiments, which revealed that nonhematopoietic cells rather than immune cells were necessary to drive the PAH phenotype (5). Given that mice with overexpression of TNF by pulmonary epithelial cells do not develop obliterative vascular lesions and have only mild PH only in the setting of emphysema (6), we surmised that PH in the TNF-Tg model is likely driven by either endothelial or mesenchymal cells.

Single-cell RNA sequencing (scRNA-seq) is a novel technology useful for simultaneous transcriptomics of thousands of cells at single-cell resolution and multiple downstream analyses, which can provide important insights into disease pathophysiology. Recent studies using this technology in human (7–9), rat (10), and mouse (11) models of PH have confirmed prior knowledge at a single-cell level, and generated new insights into potential genes and pathways that might contribute to disease pathogenesis. In this study, we sought to transcriptionally assess pulmonary endothelial and mesenchymal cells from mice with TNF-mediated PH over time and to perform velocity, cell communication, and ligand-receptor interaction analysis in addition to standard gene expression, pathway, and transcription factor analysis to better understand the microenvironment and cellular dynamics in PH and molecularly characterize the abnormal lung phenotype in the TNF-Tg model of PH. To validate and further understand the role of endothelial versus mesenchymal TNF and the specificity of TNF versus NF-κB signaling, in vitro studies using explanted WT and TNF endothelial and fibroblast cells were used to assess the role of NF-κB and TNF on BMP and SMAD expression.

## Results

### Single-cell molecular profiling identifies 15 cellular subtypes in WT and TNF-Tg lungs.

**Figure 1:**
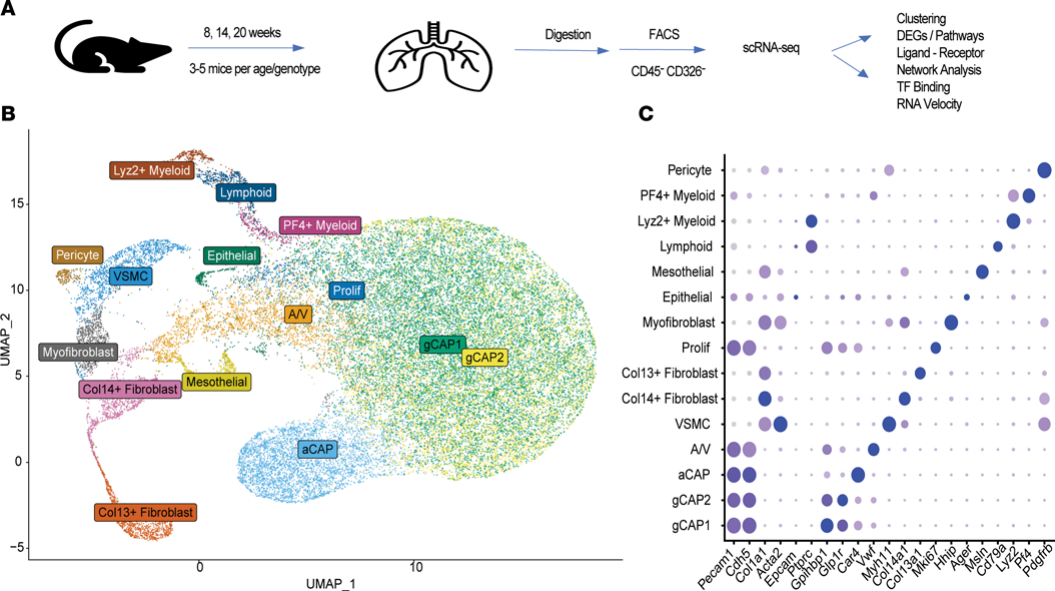
Identification and characterization of endothelial and mesenchymal cell types in scRNA-seq. (**A**) Schematic overview of study design. (**B**) UMAP projection of 15 cell types identified. (**C**) Dot plot indicating canonical endothelial (*Pecam1*, *Cdh5*), mesenchymal (*Col1a1*, *Acta2*), epithelial (*Epcam*), and immune (*Ptprc*) populations and one characteristic marker representing each cluster.

After depletion of CD45^+^ and CD326^+^ cells by cell sorting (Figure 1A, Supplemental Figure 1, and Supplemental Table 1; supplemental material available online with this article; https://doi.org/10.1172/jci.insight.174456DS1), a total of 39,184 cells were sequenced across 6 conditions (Supplemental Table 2). Fifteen cellular subpopulations were identified after performing clustering analysis across all cells (Figure 1B). The majority of cells represented endothelial cells with 5 subpopulations identified. General capillary (gCAP) endothelial cells were the most prominent population, followed by aerocyte (aCAP) and arteriovenous (A/V) endothelial cells. In addition, 5 mesenchymal populations, including vascular smooth muscle cells (VSMCs), 2 fibroblast populations (Col14^+^ matrix fibroblast and Col13^+^ lipofibroblast phenotypes) (12), as well as myofibroblasts and pericytes were identified based on canonical gene expression (Figure 1C). Reclustering in the mesenchymal subset demonstrated that the collagen 13^+^ and collagen 14^+^ fibroblast subsets further subdivided into smaller subpopulations, some of which were preferentially enriched or lost in TNF-Tg mice (Supplemental Figure 2). Despite depletion of hematopoietic and epithelial cells by flow cytometry, we still identified 3 immune cell populations, epithelial cells, and mesothelial cells, all of which represented minor cell populations and were excluded from further analysis.

### Temporal changes in endothelial and mesenchymal cell proportions in TNF-Tg lungs.

**Figure 2:**
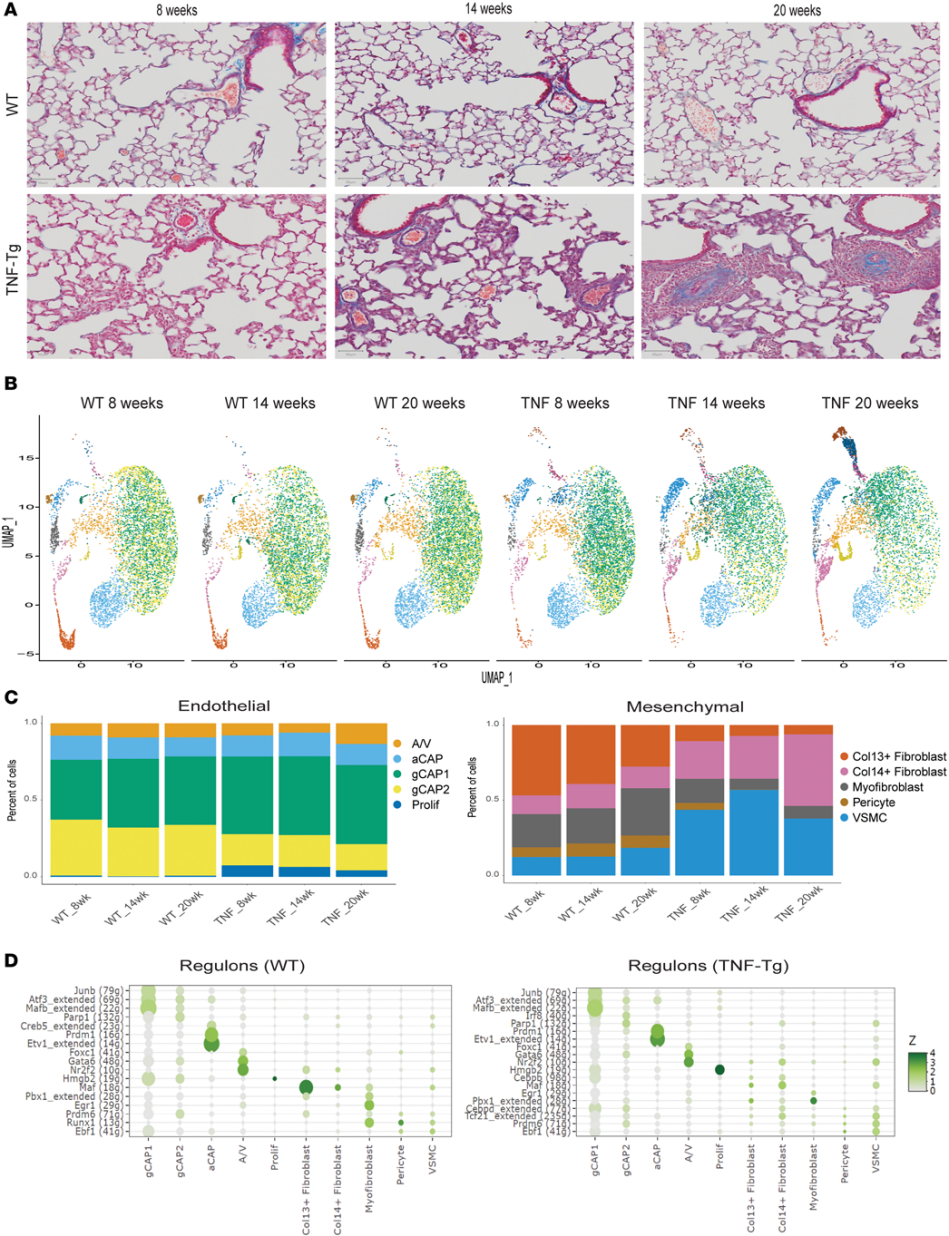
Changes in cellular composition of TNF-Tg lungs over time. (**A**) Lung histology from WT (upper) and TNF-Tg (lower) lungs: Masson’s trichrome stain. (**B**) UMAP projections demonstrating changes in cellular makeup by genotype and time point. Note increased VSMC and Col14^+^ fibroblast and decreased gCAP, Col13^+^ fibroblast, and pericyte populations in TNF-Tg mice. (**C**) Proportion of cells of each endothelial (left) and mesenchymal (right) cell subtype across WT and TNF-Tg mice at each time point. Significant change between cell proportions in WT and TNF-Tg conditions (scProportionTest *P* < 0.05). (**D**) Regulon specificity scores (RSS) were calculated for each cell type in WT (left) and TNF-Tg (right) using SCENIC and are displayed as a dot plot, with darker green indicating increased specificity of transcription factor binding in a given cell type.

As expected based on histology (Figure 2A), wild-type (WT) mice demonstrated no significant differences in cellular proportions from 8 to 20 weeks of age. In contrast, TNF-Tg mice showed little difference in cell composition compared with WT at 8 weeks of age, but showed considerable differences at 14 and 20 weeks of age. Among endothelial cells, TNF-Tg mice demonstrated a significant loss of gCAP2 cells and an increase in proliferating endothelial cells (difference in proportions *P* < 0.05, scProportionTest), while the number/proportion of gCAP1, aCAP, or A/V endothelial cells was comparable in WT and TNF-Tg lungs. Among mesenchymal cells of TNF-Tg mice, we saw a substantial increase in VSMCs, and decreases in Col13^+^ fibroblasts and pericytes, including a complete absence of pericytes by 20 weeks of age (Figure 2, B and C; *P* < 0.05, scProportionTest).

### Regulons of coexpressed genes in TNF-Tg lungs reveal cell-type-specific changes in transcription factor binding landscape in PH.

We next explored gene coexpression and its regulation by transcription factor binding using the SCENIC algorithm (13), to better understand the cause of changes in cell proportions. Analysis of regulon specificity scores (RSSs) identified 1 to 4 specific transcription factors that were active in each cell type in WT and TNF-Tg mice (Figure 2D). While most transcription factor binding was conserved across conditions, substantial changes in transcription factor binding included gCAP2 cells’ expression of an *IRF8* regulon in TNF-Tg mice, loss of *CREB5* regulon in TNF-Tg aCAP cells, decreased *MAF* regulon activity in TNF-Tg Col13^+^ fibroblasts, increased *Cebpb* regulon activity in TNF-Tg Col14^+^ fibroblasts, increased *PBX1* regulon and decreased *EGR1* and *RUNX1* regulons in TNF-Tg myofibroblasts, and loss of *Runx1* regulon in TNF-Tg pericytes (Figure 2D). Supplemental Figure 3 gives additional detail on specific regulons over- and underexpressed in WT versus TNF-Tg mice at 20 weeks of age and highlights coexpression of cell-specific markers and regulons using t-distributed stochastic neighbor embedding (t-SNE) projections, the specific binding motifs active in select cell types, and pathways of genes regulated by TNF-Tg–specific regulons in late-stage disease.

### RNA velocity analysis identifies changes in cell fate, with evidence for a gCAP to aCAP transition in TNF-Tg mice.

**Figure 3:**
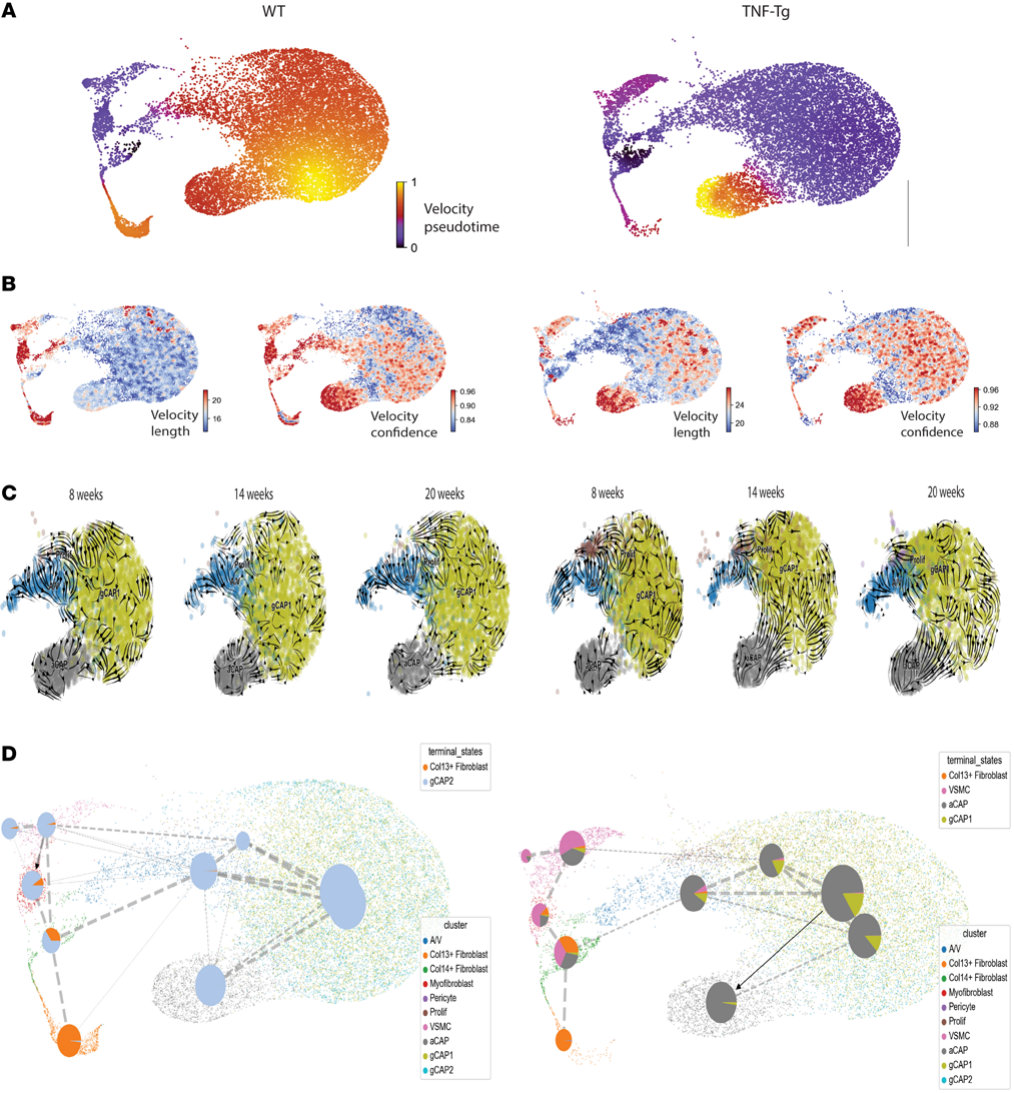
Velocity analysis demonstrates gCAP to aCAP transition in TNF-Tg mice. (**A**) RNA velocity pseudotime is shown across WT (left) and TNF-Tg (right) conditions overlaying prior UMAP plots. Purple indicates lower pseudotime and yellow indicates higher pseudotime. (**B**) Velocity length (left) and confidence (right) indicate the likelihood of cellular transition in WT (left) and TNF-Tg (right) lungs. (**C**) Velocity embeddings show vectors indicating cellular transition at 8, 14, and 20 weeks in WT (left) and TNF-Tg (right) conditions. Note prominent gCAP to aCAP transition at 14 and 20 weeks in TNF-Tg conditions. (**D**) Directed PAGA plots created with CellRank identify likelihood of given cellular fate decisions. Nodes correspond to cell type clusters and edge thickness denotes transcriptomic similarity. Directed edges reflect local velocity flow and velocity pseudotemporal ordering to ascertain initial/terminal cell states and pie charts within each node show the probability of each cell type transitioning to known terminal states. Colors indicate annotated cell populations.

We assessed cellular trajectory with RNA velocity, which is calculated on the basis of differentially spliced transcripts (Supplemental Figure 4 shows splicing by cell subset). We performed velocity analysis using scVelo (14) and compared velocity pseudotime, length, confidence, and velocity embeddings over time between endothelial cells in WT and TNF-Tg mice (Figure 3, A–C). We found that RNA velocity increased significantly in aCAP cells and smooth muscle cells and decreased in pericytes, myofibroblasts, A/V endothelial cells, and Col13^+^ fibroblasts in TNF-Tg conditions relative to WT. We utilized the Cellrank (15) algorithm to identify initial and terminal states and to assess trajectories that cells may transition through to more clearly assess cellular fates. This information, plotted as a directed partition-based graph abstraction (PAGA) plot (Figure 3D), indicates that in WT mice, most endothelial cells become gCAP2 cells, while a minority of myofibroblasts and Col14^+^ fibroblasts transform into Col13^+^ fibroblasts. In contrast, in TNF-Tg mice, endothelial cells, and most prominently gCAP cells, transition to an aCAP phenotype. Velocity embeddings demonstrate that the prominent gCAP to aCAP transition occurs primarily at 14 and 20 weeks of age in the TNF-Tg lungs (Figure 3C). In addition, a substantial portion of Col14^+^ fibroblasts, myofibroblasts, the majority of pericytes, and a minority of A/V endothelial cells transitioned to VSMCs only in TNF-Tg lungs. Supplemental Figure 5 shows velocity embeddings for all cells (Supplemental Figure 5A), fibroblasts (Supplemental Figure 5B), and mural cells (Supplemental Figure 5C).

### Global signaling changes in TNF-Tg lungs include inflammatory genes and established PH pathways.

**Figure 4:**
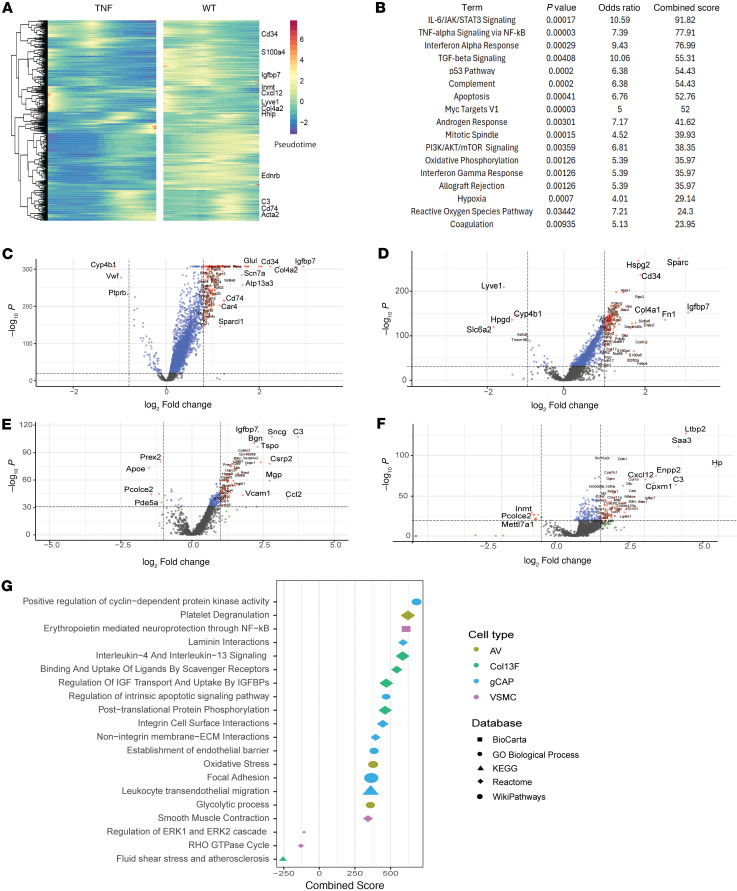
Differential gene expression and pathway changes in TNF-mediated PH. (**A**) Genes significantly altered across pseudotime in TNF-Tg mice. After trajectory analysis using Slingshot, pseudotime was calculated and genes expressing significantly altered pseudotime in TNF-Tg versus WT were calculated using tradeSeq. Heatmap demonstrates genes that vary over pseudotime. (**B**) Pathway analysis of genes with altered pseudotime in TNF-Tg conditions. Volcano plots demonstrate genes that are most differentially over- and underexpressed in TNF-Tg versus WT mice in (**C**) gCAP1 cells, (**D**) A/V endothelial cells, (**E**) VSMCs, and (**F**) collagen 13^+^ fibroblasts. (**G**) Dot plot indicating the most differentially regulated pathways in TNF-Tg versus WT cells in each of these 4 populations. Dot plots colors indicate the pathway database and the *x* axis indicates significance (*P* value, Fisher’s exact test) of overrepresentation of the pathway gCAP1 cells (blue), A/V endothelial cells (lime green), VSMCs (purple), and collagen 13^+^ fibroblasts (lime green).

We applied trajectory analysis using Slingshot to identify genes that change over RNA pseudotime and then compared genes that changed over pseudotime in TNF-Tg versus WT conditions using tradeSeq, in order to assess pathway differences across all cell populations. Genes differentially upregulated in TNF-Tg mice were plotted as a heatmap (Figure 4A) and pathway analysis was performed (Figure 4B). As expected, inflammatory pathways, including TNF/NF-κB, IL-6/JAK-Stat, interferon, and complement were the most highly overexpressed. Additional pathways relevant to PH, including TGF-β, apoptosis, oxidative phosphorylation, reactive oxygen species, and coagulation, were also increased in TNF lungs.

### Differential gene and pathway regulation in endothelial populations.

In the endothelial compartment, we focused on gCAP2 cells, which dramatically decreased in number and A/V endothelial cells, which are critical components in macrovascular lesions in PH. The most upregulated genes in gCAP2 cells included *IGFBP7*, *COL4A2*, and *CD34*, while the most downregulated genes included *CYP4B1*, *VWF*, and *PTPRB* (Figure 4C). Prominently upregulated pathways included regulation of cyclin-dependent kinase activity, laminin and integrin signaling, regulation of apoptosis, and transendothelial migration (Figure 4G). Downregulated pathways in gCAP2 cells included prostaglandin synthesis, macrophage differentiation, CCL2 production, and VEGF signaling (Supplemental Figure 6). In A/V endothelial cells, the most upregulated genes included *SPARC*, *HSPG2*, and *CD34*, while the most downregulated genes included *LYVE1*, *SLC6A2*, and *HPGD* (Figure 4D). Upregulated pathways included those related to platelet degranulation, oxidative stress, and glycolysis (Figure 4G), while downregulated pathways included neurotransmitter-related pathways, and lipid mediator pathways including lipoxins and resolvins (Supplemental Figure 6). Pathway analysis in additional endothelial populations is described in Supplemental Figure 7.

### Differential gene expression and pathway analysis in mesenchymal populations.

In the mesenchymal compartment, we focused on VSMCs, which dramatically increased in number and Col13^+^ fibroblasts, which were preferentially lost in TNF-driven PH. Among VSMCs, the most upregulated genes included *C3*, *SNCG*, and *TSPO*, while the most downregulated genes included *APOE*, *PREX2*, and *PCOLCE2* (Figure 4E). Pathway analysis demonstrated upregulation of erythropoietin-mediated neuroprotection via NF-κB, smooth muscle contraction, ERK signaling, and the RHO GTPase cycle (Figure 4G). Downregulated pathways included nitric oxide signaling, integrin-mediated cell adhesion, and antigen processing (Supplemental Figure 6). The most upregulated among the Col13^+^ fibroblast population genes included *HP*, *LTBP2*, and *SAA3*, while the most downregulated genes were *INMT*, *PCOLCE2*, and *METTL7A1* (Figure 4F). Pathway analysis revealed upregulation of IL-4/13 signaling, scavenger receptors, and insulin-like growth factor (IGF) signaling, while downregulated pathways included integrin interaction, response to LDL, and regulation of apoptosis (Supplemental Figure 6). Pathway analysis in additional mesenchymal populations is described in Supplemental Figure 7.

### Pathways downstream of TNF receptors.

To address the relative contribution of TNF/TNF receptor signaling in this PH model, we developed gene modules to represent 7 signaling cascades (FADD/TRADD-mediated apoptosis, RIP3K-mediated necroptosis, PI3K, MAPK, JAK-STAT, NF-κB, and p38-ERK), which are known to signal downstream of either TNF receptor 1 or TNF receptor 2. Gene module scores were developed and compared between WT and TNF-Tg mice across all endothelial and mesenchymal cell types (Supplemental Table 3). While some cell types demonstrated changes in many of these pathways, others were more specific. For example, pericytes demonstrated a prominent FADD/TRADD-mediated apoptosis signature.

### Ligand-receptor interaction modeling demonstrates central role of altered BMP signaling.

**Figure 5:**
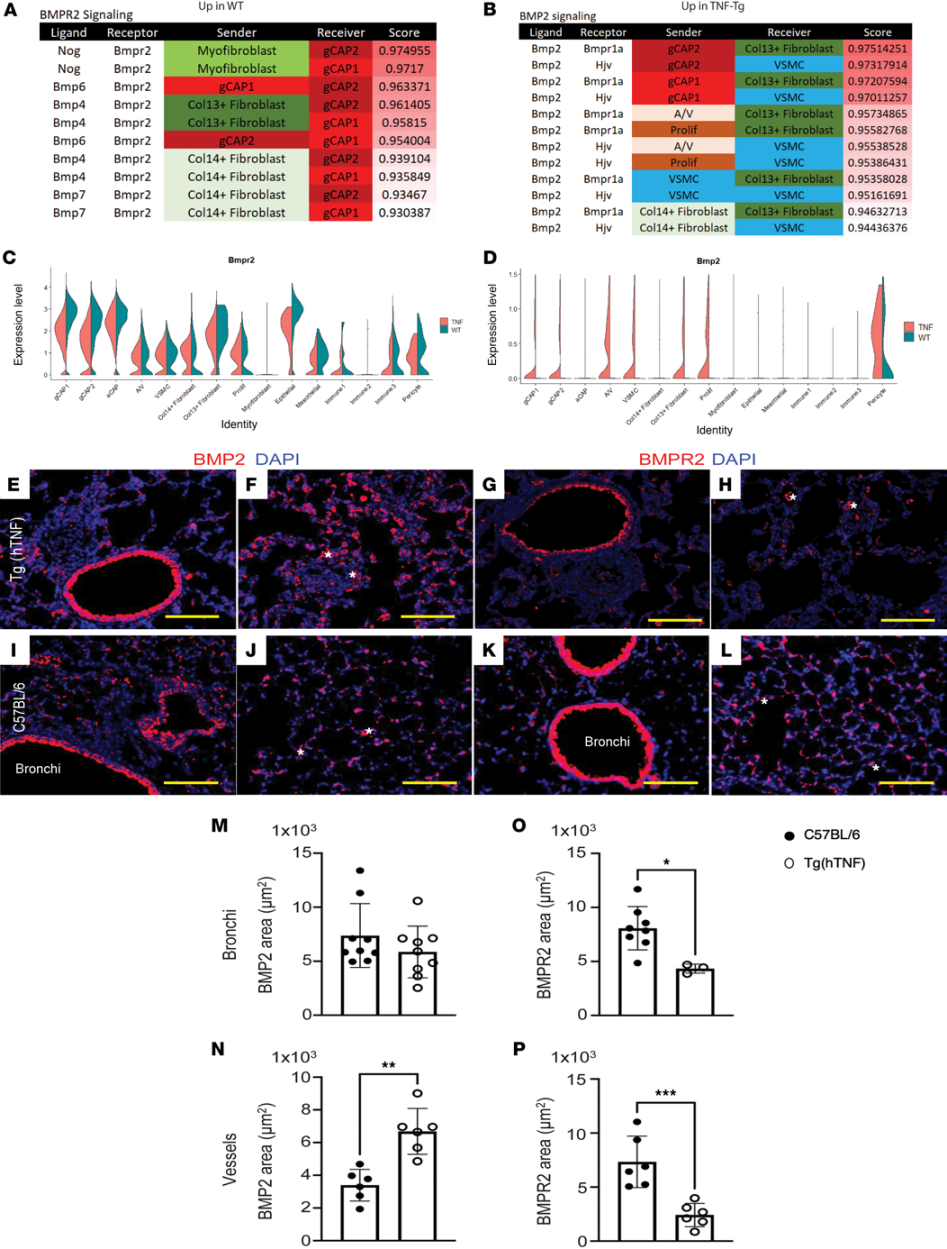
Ligand-receptor analysis demonstrates prominent role of altered BMP signaling in TNF-mediated PH. (**A**) BMPR2 signaling in TNF-Tg mice. MultiNicheNet was used to identify ligand-receptor pairs that were specific to either WT or TNF conditions. WT lungs demonstrated strong gCAP *Bmpr2* interactions with *Bmp4*, *Bmp6*, *Bmp7*, and *Nog* in fibroblasts, which were absent TNF-Tg conditions. (**B**) Aberrant *Bmp2* signaling in TNF-Tg lungs. TNF lungs demonstrated the presence of interactions between *Bmp2* in a variety of cells interacting with Hjv in smooth muscle cells and Bmpr1a in Col13^+^ fibroblasts, and these interactions were absent in WT lungs. (**C**) Decreased expression of *Bmpr2* in TNF-Tg gCAP cells. Violin plots demonstrate expression across cell types. (**D**) Increased expression of *Bmp2* in TNF-Tg lungs. Lungs from TNF-Tg and C57BL/6 mice were stained with antibodies specific for BMP2, BMPR2, and nuclei labeled with DAPI. BMP2 expression was comparable in the bronchial regions in the lungs of (**E**) TNF-Tg and (**I**) C57BL/6 mice. In contrast, BMP2 expression increased in the vascularized areas in the lungs of (**F**) TNF-Tg mice compared with (**J**) C57BL/6 mice. BMPR2 expression decreased in the bronchial areas of (**G**) TNF-Tg mice compared with (**K**) C57BL/6 mice. BMP2 pulmonary expression was higher in vascularized regions of (**H**) TNF-Tg mice than (**L**) C57BL/6 mice. Asterisks depict lumen of blood vessels. Scale bars: 100 μm. Three random pictures were taken (original magnification, ×200) in bronchial and vascularized areas in the lungs of TNF-Tg (*n* = 2–3, 3–9 pictures) and C57BL/6 mice (*n* = 2–3, 6–9 pictures). Representative pictures (×200) are shown. Area of BMP2^+^ or BMPR2^+^ signal in ×200 random fields was measured with NIH ImageJ. Area covered by BMP2 in (**M**) bronchial areas was comparable in TNF-Tg and C57BL/6 mice, while BMPR2 area was significantly increased in (**O**) bronchial areas of C57BL/6 mice. Area covered by (**N**) BMP2 and (**P**) BMPR2 in vascularized regions in the lungs of TNF-Tg mice was significantly larger than areas of BMP2 and BMPR2 signal in C57BL/6 mice. Graphs represent the mean ± SEM. Statistical significance was calculated with a paired, 2-tailed Student’s *t* test. *P ≤ 0.05, **P ≤ 0.005, ***P ≤ 0.0005.

Ligands and receptors were identified in each cell type using MultiNicheNet, an R package derived from NicheNet (16) (Supplemental Figures 8 and 9). When we assessed the ligand-receptor (L:R) interactions that were differentially expressed in the WT and TNF conditions, there was a striking central role of interactions centering on BMP signaling. In WT lungs, *BMPR2* served as a receptor for 8 of the 11 strongest L:R interactions, while in TNF-Tg lungs, *BMP2* served as a ligand in 9 of the 13 strongest L:R interactions. *BMPR2* is predicted to interact with *NOG*, *BMP4*, and *BMP7* only in WT cells, while *BMP2* is predicted to interact with *BMPR1A* in fibroblasts and hemojuvelin (*HJV*) in VSMCs only in TNF-Tg lungs (Figure 5, A and B). When we examined gene expression levels, *BMPR2* was significantly downregulated in TNF-Tg microvascular endothelial cells (gCAP and aCAP) compared with WT (Figure 5C), while *Bmp2* was aberrantly expressed in a variety of cells from TNF-Tg mice (endothelial, VSMCs, Col13^+^ fibroblasts), but no expression was seen in WT cells (Figure 5D). Given these striking findings, we sought to confirm changes in BMP signaling at the protein level by using multicolor immunofluorescence (Figure 5, I–P). We confirmed a decrease in endothelial BMPR2 (Figure 5E) and its ligand BMP4 in the perivascular region (Figure 5G) and an increase in perivascular BMPR1a (Figure 5F), HJV (Figure 5H), and their ligand BMP2 (Figure 5H) in 20-week-old TNF-Tg mice. We also observed a marked increase in NOG staining in TNF-Tg lungs (Figure 5, E–G). We confirmed increased NOG expression in TNF-Tg lungs compared with WT, which was mostly found in perivascular areas of the lung. To confirm that this signal was from nonhematopoietic cells, we performed costaining with CD45 and found that the vast majority of NOG^+^ cells did not costain with inflammatory cells (Supplemental Figure 15).

### Decreased angiogenesis and increased TGF-β/collagen/integrin signaling.

**Figure 6:**
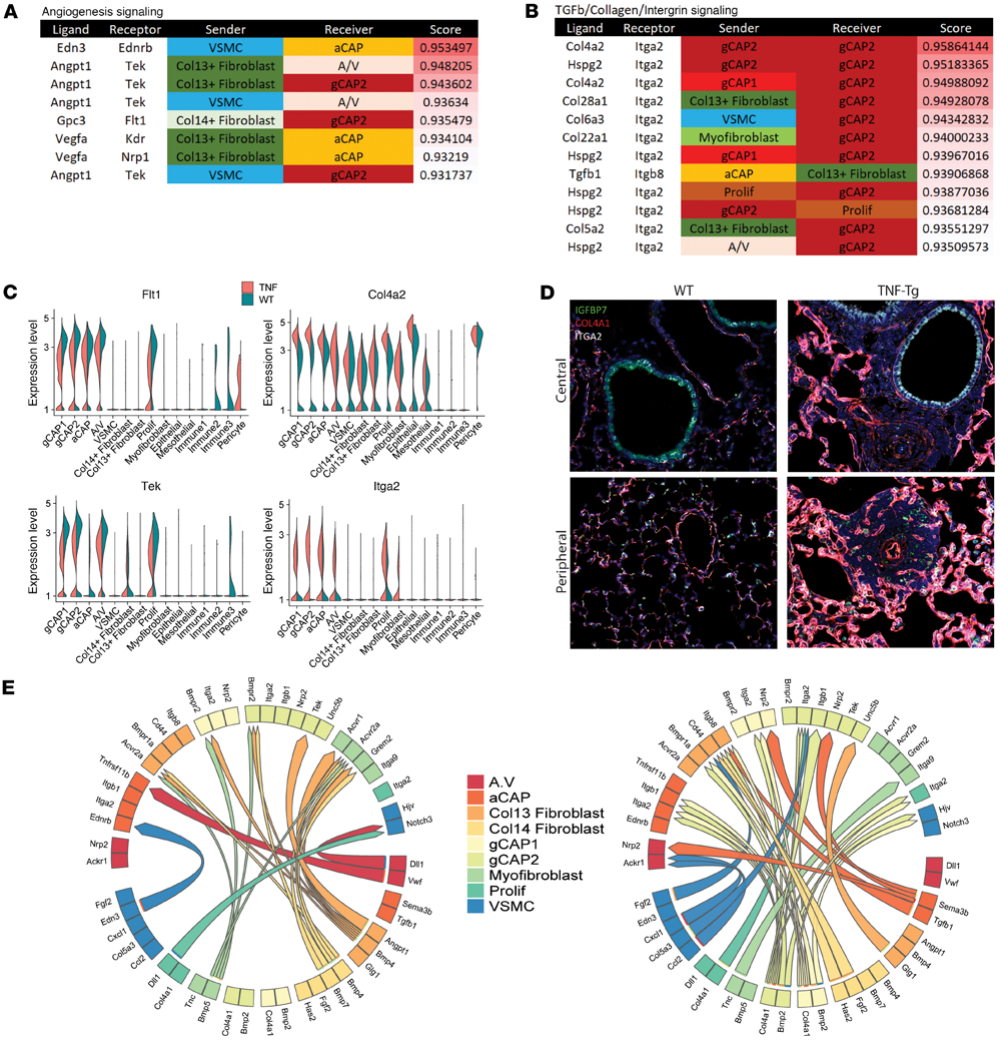
Decreased angiogenesis and increased integrin/basement membrane signaling in TNF lungs. (**A**) Loss of angiogenesis signaling in TNF-Tg mice. MultiNicheNet was used to identify ligand-receptor pairs that were specific to either WT or TNF conditions. WT lungs demonstrated strong Tek, Flt1, Kdr, and Ednrb interactions with Angt1, Vegfa, Gpc3, and Edn3, which were absent TNF-Tg conditions. (**B**) Increased TGF-β/integrin/basement membrane signaling in TNF-Tg lungs. TNF-Tg lungs demonstrated the presence of interactions between Itga2 (primarily in gCAP2 cells) and multiple collagen genes, Hspg2, and Tgfb1. (**C**) Decreased expression of Flt1 and Tek and increased expression of Col4a2 and Itga2 in TNF-Tg gCAP cells. Violin plots demonstrate expression across cell types. (**D**) Immunostaining confirms increased expression of Col4a1, Itga2, and Igfbp7 in TNF-Tg lungs. Original magnification × 200. (**E**) Circos plots indicating most differentially regulated ligand-receptor pairs ascertained by MultiNicheNet in WT lungs (left) and TNF-Tg (right) lungs; sender cells are listed on the bottom, while arrows point to receiver cells on the top.

In addition to BMP signaling, 2 additional pathways became evident in the L:R analysis. We found the presence of proangiogenic L:R pairs such as *VEGFA/KDR*, *ANGPT1/TEK*, and *EDN3/EDNRB* exclusively in WT lungs (Figure 6A). In contrast, TNF-Tg lungs expressed substantial interactions of integrin α2 (*ITGA2*), a known receptor for collagens (*COL4A2*, *COL5A2*, *COL6A3*, *COL22A1*, and *COL28A1*), *HSPG2*, and *TGFB1* (Figure 6B). We found upregulation of collagen IV and integrin α2 in multiple TNF-Tg cell types (Figure 6C) and confirmed this finding by multicolor immunofluorescence (Figure 6D and Supplemental Figure 16).

### Network analysis of changes in cell interactions in TNF-mediated PH.

We used the differential implementation of CellChat to better understand global cell-cell communication (17). We found that while TNF-Tg lungs had a greater number of cellular interactions, these interactions tended to be weaker than those seen in WT mice (Supplemental Figure 13A). The cell type with the greatest increase in differential interactions in TNF-Tg relative to WT mice was the Col13^+^ fibroblast (Supplemental Figure 13B). We visualized the relative strength of differential pathway expression in each cell type (Supplemental Figure 13C) and found pathways overexpressed in TNF-Tg cells included TRAIL, RANKL, IL-2, CXCL, periostin, Kit, and TGF-β. In contrast, PDGF, SPP1, MIF, Visfatin, EGF, ANGPT, WNT, VEGF, BMP, EDN, ANGPTL, and ncWNT pathways were upregulated in WT lungs. Cellular communication was then visualized as incoming and outgoing signaling modules, alluvial plots, and pathway signaling pathways (Supplemental Figure 13D).

The L:R findings from NicheNet were visualized with Circos plots highlighting the differences in signaling (Figure 6E) and a network analysis of these findings demonstrated the centrality of BMP4/BMPR2 signaling in WT and BMP2/COL4A1/FN1 and loss of VEGFA signaling in PAH lung (Supplemental Figure 14).

### In vitro assessment of BMP and SMAD signaling in explanted endothelial cells and fibroblasts.

**Figure 7:**
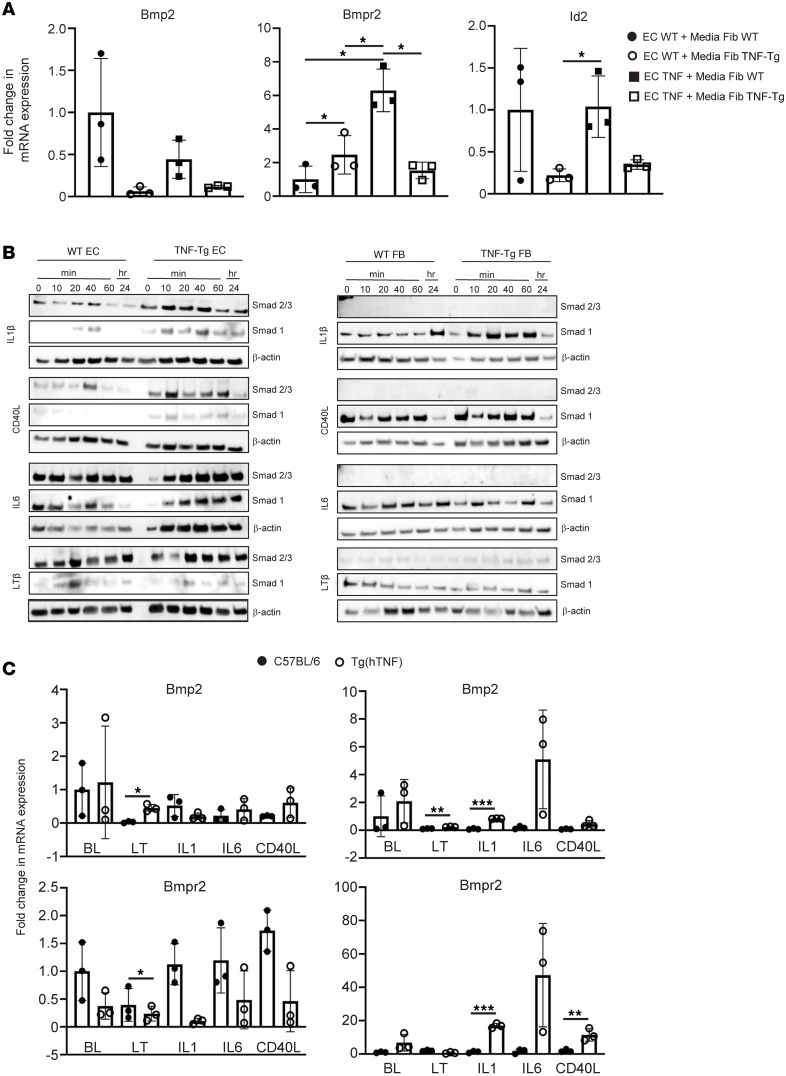
Cell-specific effects of NF-κB activators and conditioned media from TNF-activated fibroblasts (FBs) on BMP2/BMPR2 and SMAD signaling. (**A**) Endothelial cells (ECs) from C57BL/6 mice (WT) or TNF-Tg mice (TNF) were cocultured with media from fibroblasts activated with TNF for 72 hours. RNA was isolated from cocultures 6 hours later and cDNA prepared for qPCR. Gene expression was normalized to *18s* and gene expression in cocultures was compared to ECs from C57BL/6 mice stimulated with media from WT FBs stimulated with TNF. *P < 0.05 by 1-way ANOVA, and by Student’s 2-tailed *t* test. (**B**) Western blot analysis shows a global increase in Smad 2/3 in endothelial cells (left) of TNF-Tg mice stimulated with NF-κB agonists, which was not seen in fibroblasts (right). (**C**) BMP2, BMPR2, and ID2 in TNF-Tg ECs are downregulated by media from TNF-Tg FBs. FBs or ECs were stimulated with NF-κB agonists to determine their impact on BMP2 and BMPR2 expression. Gene expression was normalized to *18S* compared to cells incubated with PBS (BL). LTβ increases BMP2 in ECs and FBs of TNF-Tg mice, while BMPR2 is globally reduced in stimulated fibroblasts of TNF-Tg mice. *P ≤ 0.05; **P ≤ 0.01; ***P < 0.001 by Student’s 2-tailed *t* test.

Conditioned media transfer experiments were performed to determine the specificity of the effects of BMP2 and BMPR2 and their signaling (assessed using ID2) on endothelial and fibroblast cell interactions. We found that TNF fibroblast media was able to effectively suppress the expression of BMP2 and ID2 in both WT and TNF-Tg endothelial cells and that BMPR2 was suppressed only when TNF-Tg endothelial cells were treated with TNF-Tg fibroblast media (Figure 7A). Treatment of fibroblasts with endothelial cell–conditioned media did not show significant differences in BMP2, BMPR2, or ID2 regardless of the source of the conditioned media (Supplemental Figure 17).

To address whether the changes in BMP signaling and TGF-β/SMAD activation are specific to TNF signaling or may be induced by alternative means of activating NF-κB, we isolated primary WT and TNF-Tg endothelial cells and fibroblasts and stimulated them with 4 cytokines known to activate NF-κB signaling through non-TNF pathways: IL-1, IL-6, CD40L, and LTAβ. All of these stimuli were able to induce SMAD2/3 signaling in endothelial cells (Figure 7B), and some (IL-1, LTA) were able to recapitulate gene expression changes in BMP2, ID2, and BMPR2 (Figure 7C).

## Discussion

The TNF-Tg model of PH molecularly recapitulates many features of human PH, including VSMC proliferation with associated extracellular matrix (ECM) deposition, loss of peripheral endothelial cells and pericytes, and changes in fibroblast subtypes. These changes in cellular composition appear to be driven by TNF signaling, which leads to loss of BMPR2 signaling, increase in TGF-β/integrin signaling mediated by increased basement membrane and other ECM proteins, and loss of vasodilatory signals. Analysis of scRNA-seq data in this model reveals a complex network of cell-cell interactions, which are present in normal lung and lost in response to TNF signaling and the resulting landscape of aberrant signaling in the PH microenvironment. Moreover, the PH transcriptional niche is regulated by a unique set of transcription factors and leads to a newly described gCAP to aCAP transition.

We previously described the TNF-Tg model of PH as having multiple pathologic features consistent with human disease, including obliterative vascular lesions with loss of peripheral lung vasculature, substantially elevated pulmonary arterial pressures (average RV systolic pressure of 75 at 5.5 months), and RV hypertrophy (5). Given that these mice also develop prominent arthritis and have a cellular nonfibrotic interstitial component to their lung disease, along with lung genomic studies indicating their similarity to scleroderma and other CTDs, we have proposed that this represents a model of CTD-PAH.

This study using scRNA-seq now confirms that the TNF-Tg model also recapitulates multiple facets of human PH at the molecular level, including a central role for loss of BMPR2 signaling, which is the most common cause of genetic PAH (3) and broadly dysregulated across PH of different etiologies. A recent study using scRNA-seq in rat models of PH, including both the monocrotaline and Sugen-hypoxia models, demonstrated that TNF/NF-κB signaling is a central molecular pathway in these well-characterized PH models (10) and this overlap suggests the TNF and particularly TNF signaling through NF-κB is likely an important upstream driver of PH across models. This is also in concordance with data that have shown that patients with idiopathic and hereditary PAH have increased local vascular expression of TNF and that TNF/NF-κB signaling selectively reduces BMPR2 mRNA and BMPR2 protein in distal VSMCs and pulmonary arterial endothelial cells, suggesting that TNF may also play a critical role in human disease (18).

A recent scRNA-seq study has shown that patients with systemic sclerosis–associated PAH (SSc-PAH) have altered endothelial cell subphenotypes, altered SMAD signaling, an endothelial-mesenchymal transition (EndMT) phenotype, and that altered VEGF, TGF-β, and members of the TNF superfamily (TWEAK) can regulate this phenotype (19). Further studies will need to be performed in order to more directly compare and contrast the human and mouse studies and determine which features are species or model/disease specific. As TNF is an important part of the secreted as part of the senescence-associated secretory phenotype (SASP), it is possible that increased cellular senescence may be a reason that some patients with CTDs develop PAH. Of interest, another SASP protein, IGFBP7, is encoded by the most highly upregulated gene in gCAP cells in our dataset and is also highly upregulated in other cell types (Figure 4, C–F). This molecule has previously been shown to be elevated in SSc-PAH (20) and to be a risk factor for PAH severity in SSc and other forms of PAH (21, 22).

In the endothelial compartment, we identified 5 cellular subsets: 2 clusters of gCAP endothelial cells, A/V, aCAP, and proliferating endothelial cells, consistent with other descriptions of pulmonary endothelial cell populations (11, 23, 24). Intriguingly, gCAP1 cells remained stable over time in the TNF-Tg mice, while there was a progressive loss of gCAP2 cells. Proliferating endothelial cells increased, while no significant changes were seen in the numbers of A/V or aCAP cells. This is in line with prior studies, which demonstrated both endothelial apoptosis and increased endothelial proliferation in PH (25). While we were able to identify genes specific for gCAP1 and gCAP2, these distinctions are not well established in the literature. However, we interestingly found that gCAP2 have reduced numbers of spliced transcripts, and that gCAP2 cells from TNF-Tg mice had increased *IRF8* transcription factor binding, suggesting a role for interferon signaling in cell death. When we performed pathway analysis across different endothelial cell populations, we found a number of intriguing changes, including upregulation of vascular wound healing, coagulation, TGF-β production, and apoptosis. We also noticed downregulation of prostaglandin synthesis, macrophage differentiation, and VEGF signaling in gCAP cells, and upregulation of lipid metabolism and downregulation of neurovascular signaling genes in A/V cells.

Among the most interesting findings in the endothelial compartment are the changes in cellular transitions identified using RNA velocity analysis. WT mice demonstrated that most endothelial subtypes tend to transition toward gCAP2 cells. In stark contrast, the vast majority of endothelial cells and even many mesenchymal cells in the TNF-Tg lung transitioned toward aCAP cells. Thus, it is likely that the substantial loss of gCAP2 cells seen in TNF-Tg lungs is explained at least partially by gCAP to aCAP transition rather than by cell death. Based on aCAP cells’ central role in gas exchange (26), the capacity of TNF to induce endothelial apoptosis (27, 28) and the transcriptional evidence of apoptosis in all endothelial subsets, we speculate that the lung preferentially replaces dying aCAP cells to ensure oxygenation for as long as possible at the expense of other endothelial (gCAP) cells. EndMT is well described in PH (29); velocity analysis suggested that a small number of A/V endothelial cells transition to become VSMCs at later time points in this model, but this did not appear to be a dominant cellular fate change. Given that this was a minor change in a small number of cells and was only seen in the velocity analysis, more definitive studies are needed to confirm whether this in fact represents EndMT.

Among mural cells, we found increased numbers of VSMCs, and a striking loss of pericytes, which was complete by 20 weeks of age in the TNF-Tg mice. Among VSMCs, pathway analysis demonstrated increased cardiac muscle differentiation, WNT signaling pathways, and downregulation of nitric oxide and antigen-processing pathways, suggesting that these cells are actively differentiating, do not produce vasodilatory signals, and lose their role in immune regulation. While we expected a loss of pericytes, the near complete loss at 14 weeks and complete absence of these cells at 20 weeks of age was striking. Looking only at the 8-week time point (data not shown), CellChat analysis demonstrated loss of *SPP1* signaling and increase in periostin pathway in these cells, while transcription factor binding analysis showed loss of *EBF1*, a marker of pericyte commitment (30). TNF-Tg VSMCs at later time points expressed a *PRDM6* regulon, which is an epigenetic regulator of smooth muscle cell phenotypic plasticity that suppresses differentiation and maintains the proliferative potential of VSMCs (31). Interestingly, in WT conditions, velocity analysis showed that VSMCs tended to differentiate into pericytes, but this phenotype was lost in TNF-mediated PAH. This suggests that, in addition to apoptosis, the loss of pericytes may be partially due to the VSMC program driven by *PRDM6*, which may prevent this transition (31). Further study of the loss of pericytes is likely to yield important insights into this model, and lineage tracing studies and/or a more careful time course with staining for multiple markers of pericytes, subsets of fibroblasts, and smooth muscle cells, while beyond the scope of this study, could more definitively address whether pericytes are replaced by other mesenchymal cells.

In the fibroblast compartment, we found a decrease in the number of myofibroblasts, and saw a change in fibroblast phenotype with a loss of Col13^+^ fibroblasts and an increase in Col14^+^ fibroblasts. Col13^+^ fibroblasts express markers such as *PLIN2* suggestive of lipofibroblasts as well as more undifferentiated fibroblast markers such as Pi16 and have been reported to consist largely of adventitial fibroblasts, whereas Col14^+^ fibroblasts express *DCN*, *COL1A1*, and *COL1A2*, which are more suggestive of matrix fibroblasts. Pathway analysis demonstrated increases in collagen and ECM production, BMP pathway changes, and complement activation, all relevant pathways in PH (3, 32, 33). Transcription factor binding analysis showed an increase in *TWIST1* in Col13^+^ fibroblasts that is known both as an EMT and EndMT factor and plays a role in PH (34, 35), suggesting that these cells may be taking on more of a matrix-producing role. Myofibroblasts and mesothelial cells overexpressed transcription factor *WT1*, which can drive fibroproliferation in fibrotic lung disease (36). In the RNA velocity analysis, we found that Col14^+^ fibroblasts had the greatest ability to differentiate into other cell types (other fibroblasts, VSMCs, and endothelial cells), while Col13^+^ fibroblasts remained very restricted to their lineage. We found heterogeneity within Col13^+^ fibroblasts, a population described to have a lipofibroblast phenotype (12). The previously described *INMT-*expressing lipofibroblast subset was largely lost in TNF-Tg lungs, whereas the cells that remained preferentially expressed *ADH7*, a marker of fibroblasts in adventitial cuffs and near arteries in bronchovascular bundles (37).

One of the innovative aspects of this study is the granular detail of the cell-cell communication and L:R interactions occurring between lung endothelial and mesenchymal cells in PH. Using MultiNicheNet, an R package derived from NicheNet that allows for comparison of L:R interactions in multisample and multicondition studies, we detected differential intracellular signaling across the multiple cell types studied. In exploring the most upregulated and downregulated L:R pairs in TNF-Tg relative to WT across cell types, the most striking finding was the change in BMP signaling, particularly downregulation of *Bmpr2* in TNF-Tg lung endothelial cells. *BMPR2* is highly expressed in WT endothelial cells and binds ligands that include *NOG*, *BMP4*, *BMP6*, and *BMP7* in multiple cell types, but these interactions are lost in TNF-Tg lungs.

Loss-of-function mutations in *BMPR2* account for approximately 70% of genetic PAH, and reductions in BMPR2 protein have been reported in PAH patients with and without *BMPR2* mutations, suggesting that this receptor likely contributes to disease pathogenesis independently of etiology. Endothelial cells from PH patients with *BMPR2* mutations demonstrate increased proliferation, reduced monolayer integrity, and increased susceptibility to apoptosis. Silencing of *BMPR2* in normal pulmonary artery endothelial cells reproduces many of the PAH features. Mice lacking BMPR2 develop spontaneous PH, and BMPR2 activity is decreased in other animal models of PH. Previous studies have suggested that TNF can lead to BMPR2 loss in smooth muscle cells (18, 38). Our findings confirm the centrality of TNF in driving a decrease in the expression of *BMPR2* by smooth muscle and endothelial cells. Given the importance of this pathway in pathophysiology, we performed immunostaining to confirm the validity of the ligand-receptor analysis and indeed found decreased BMPR2 and BMP4 proteins in the TNF-Tg pulmonary vessels relative to WT. Loss of BMPR2 by itself does not recapitulate TNF overexpression, and whether an additional TNF-mediated pathway (such as endothelial senescence) beyond loss of BMPR2 promotes the profound remodeling and PAH in this model remains to be determined.

In the absence of *BMPR2*, the ligand-receptor analysis indicates that TNF-Tg mice demonstrate an altered BMP signaling network in which multiple cells aberrantly produce *BMP2*, which binds *BMPR1A* on fibroblasts and *HJV* on VSMCs. These findings were also confirmed histologically, with increased BMP2 in proximity to HJV and BMPR1A in perivascular areas of TNF-Tg lungs, and the absence of these staining patterns in WT mice.

BMP2 is upregulated in response to hypoxia, protecting against hypoxia by upregulating eNOS expression. HJV functions as a BMP coreceptor that binds BMP ligands (primarily BMP2/4) and receptors to enhance SMAD phosphorylation, but these interactions and signaling pathways have not been described in PH. Interestingly, BMP/HJV signaling is necessary for hepcidin production and iron regulation (39), and could contribute to PH patients’ frequent iron deficiency (40).

Conditioned media experiments were conducted to confirm the results of the ligand-receptor interaction analysis with respect to BMP2 and BMPR2. Interestingly, BMP2 and ID2 (a downstream BMP responsive gene) were suppressed in endothelial cells by TNF-Tg fibroblast media regardless of the endothelial genotype, while TNF-Tg endothelial cells were required to demonstrate suppression of BMPR2, suggesting that something intrinsic to the TNF-Tg endothelial cells, likely NF-κB activation, is needed to be responsive to the TNF mesenchymal cytokine milieu.

As multiple cytokines other than TNF that stimulate NF-κB signaling were able to induce SMAD2/3 protein in endothelial cells, this suggests that endothelial NF-κB signaling is a key downstream regulator of BMP and SMAD signaling, but that this is not specific to TNF. These findings need to be interpreted cautiously, as they are drawn from total SMAD expression and did not specifically address phosphorylated SMAD proteins. It is possible that transcriptional change mediated by regulons such as *CREB5*, which we found to be altered in TNF-Tg endothelial cells, may be drivers of SMAD2/3 activation, but further studies will be needed to confirm this.

Staining for Noggin, an extracellular inhibitor of BMP signaling commonly upregulated in PH, was increased in TNF-Tg lungs. The fact that we found increased Noggin protein expression is likely due to Noggin’s long-lived nature and accumulation in the ECM, indicating that its mRNA expression (which is expressed at low levels, and may be early or transient) does not explain its function in this context where it likely binds free BMP2. To support the notion of early Noggin expression, unlike other BMP proteins, there was marked increased Noggin staining in 8-week-old TNF-Tg lungs.

PH has been frequently described as requiring both downregulation of BMP signaling and an upregulation of TGF-β signaling. Our L:R interaction module, as well as our pathway analysis, showed substantial upregulation of ECM proteins (collagens), integrin signaling, focal adhesion, and basement membrane proteins in TNF-driven PH. TNF cell-cell communication is marked by increases in *COL4A1*, *COL4A2*, *COL6A3*, *COL28A1*, *TGFB1*, and *HSPG2* molecules interacting with integrins (primarily integrin a2) on a variety of cells. Collagen IV and HSPG2 are both key basement membrane proteins, and it was recently shown that PH lungs have substantial remodeling of the basement membrane (41). The prominence of collagen IV staining in the interstitium shows that this protein is produced primarily by gCAP cells. This suggests that overproduction of endothelial basement membrane proteins may account for a substantial portion of the interstitial lung disease component described in TNF-Tg mice, especially considering a lack of overexpression of fibrillar collagens by TNF-Tg fibroblasts and myofibroblasts. Moreover, given that collagen IV is typically produced by pericytes that are no longer present in capillaries, this likely represents the endothelial cells trying to maintain barrier integrity lost by pericyte death. In addition to BMP and TGF-β signaling, other key vascular ligands lost in TNF conditions include *VEGFA*, *IGF2*, *EDN3*, *ANGPT1*, and *LPL*. This indicates that vasodilatory pathways, many of which are treatment targets in PH, are also altered by TNF overexpression.

Some of the limitations of the study include the use of flow cytometry to focus on endothelial and mesenchymal cells and the exclusion of other key cells in PAH, including immune cells and epithelial cells. The TNF-Tg mouse does have a more prominent inflammatory infiltrate than is seen in other rodent models or idiopathic PAH. Our prior work described the model as more closely recapitulating CTD-PAH using whole-lung RNA-seq, but given that we showed nonhematopoietic cells were responsible for the phenotype using bone marrow chimera experiments (5), we excluded immune cell types in the present analysis, which limits our ability to understand the complex molecular dialogue between immune cells and stromal cells in this study. While we did sequence a small number of immune cells, these were excluded from the analysis because our flow cytometry depleted CD45^+^ cells and these cells therefore likely have a skewed phenotype. While speculative, it is interesting to note that a large number of the myeloid cells sequenced highly express PF4 (*CXCL4*) that is expressed by plasmacytoid dendritic cells and is associated with PAH in patients with systemic sclerosis (42), although this population might also contain megakaryocytes. Furthermore, beyond the confirmatory staining for the BMP pathway, our findings do not give spatial context to the cellular interactions seen and future studies should confirm findings using high-depth immunostaining, mass cytometry, or spatial transcriptomics to visualize the cellular and molecular processes and interactions within the PAH microenvironment. While many of the findings in the TNF-Tg model are shared with PAH patients and other rodent PH models, the RNA velocity analysis findings including the gCAP to aCAP transition should be confirmed in other models and PAH patients, and lineage tracing would be useful to more definitively confirm cellular fate transitions. Another limitation is that we were not able to subset A/V endothelial cells into arterial and venous using clustering or previously described markers, and therefore the A/V cluster lacks ideal resolution to answer questions specifically about arterial endothelial cells.

The potential for anti-TNF therapy as a treatment target in PH and particularly in CTD-PAH is attractive, and studies looking at drug repurposing for PH have identified anti-TNF as a strong potential candidate (43). We previously showed that anti-TNF therapy prevents disease progression in TNF-Tg mice (5), and other groups observed improvement or resolution of disease using anti-TNF therapy in rat Sugen-hypoxia (18) and monocrotaline models (44), and a pig model of endotoxin-induced PH (45). While no prospective clinical trials of anti-TNF therapy have been performed in PH and there is concern due to a study in which infliximab caused worsening heart failure (46), there are case reports of patients with scleroderma and rheumatoid arthritis who experienced worsening of PAH after cessation of anti-TNF therapy (47, 48) suggesting potential benefit particularly in the setting of CTD-PAH.

In conclusion, our data provide strong evidence that TNF is a central player in the pathophysiology of PH and that overexpression of TNF causes a cascade of cellular and molecular changes linked to the endothelial and mesenchymal phenotypes seen in this devastating disease, including loss of BMPR2 signaling. Our results suggest the utility of the TNF-Tg model of PH for designing mechanistic studies and provide further rationale for anti-TNF clinical studies of patients with CTD-PAH.

## Methods

### Sex as a biological variable.

Based on previous work in which we have longitudinally assessed sexual dimorphisms of cardiopulmonary phenotype of TNF-Tg mice, we demonstrated that early mortality in female, but not male, mice is due to PH and heart failure (5). We therefore utilized female mice in the present study so that we could examine the entire spectrum of severity of disease in this model.

### Mice.

The TNF-Tg mouse line 3647 (49) was generated by Georgios Kollias of the Alexander Fleming Biomedical Sciences Research Center (BSRC) and was used with permission of Alexander Fleming BSRC. The PH phenotype in TNF-Tg and WT mice has been previously described (5). In this study, we characterized cells from 3–5 female mice at each of 3 time points: 8 weeks, 14 weeks, and 20 weeks of age. Based on prior experience with the TNF-Tg model in which female mice develop histologic evidence of pulmonary vasculopathy between 9 and 12 weeks of age, have significantly elevated RV pressures by 3–4 months of age, and die by 5.5–6 months of age (5), we chose these time points to represent a natural history of disease: 8 weeks represents an early time point prior to onset of PH, 14 weeks a point with established PH but without overt heart failure, and 20 weeks of age represents end-stage disease.

### Lung digestion.

Lungs were pooled by genotype/age and single-cell suspensions were obtained from the lungs mice by a combination of mechanical dissociation and enzymatic digestion. Lungs were first dissociated with a micro-scissor and a GentleMACS Octo Dissociator (Miltenyi Biotec). Dissociated lungs were then digested at 37°C and 5% CO_2_ for 30–45 minutes in digestion buffer containing 10 mM *N*-2-hydroxyethylpiperazine-*N*-2-ethane sulfonic acid (HEPES), 150 mM NaCl, 5.0 mM KCl, 1.0 mM MgCl_2_, 1.8 mM CaCl_2_, 0.3–0.4 U/mL collagenase A (COLLA-RO, Roche), 800 U/mL DNase I (Sigma-Aldrich, DN-25), 1.5–2.0 U/mL elastase (Worthington, ESL LS002292), and 10 U/mL Dispase II (Corning, 354235). The enzymatic reaction was neutralized by Dulbecco’s PBS (DPBS) containing 10% FBS and red blood cells were removed by ACK Lysing Buffer (Thermo Fisher Scientific, A104920). Cells were pelleted at 1500 rpm (462*g*) at 4°C for 10 minutes, washed twice with FACS staining buffer (DPBS containing 5% FBS and 2 mM EDTA) and resuspended into FACS buffer at 1 × 10^7^ to 5 × 10^7^ cells/mL.

### Flow sorting to deplete bone marrow–derived and epithelial cells.

The single-cell suspensions were then stained with a fluorescently conjugated antibody specific for CD45 (clone 30-F11, PE-Cyanine 7, BioLegend), CD326 (PE-Dazzle 594, BioLegend), and CD31 (APC-Fire 750, BioLegend) for 30 minutes at 4°C in the dark after incubation with FcBlock (BD Biosciences, anti-CD16/anti-CD32) for 15 minutes at 4°C. The cells were washed with FACS staining buffer twice and a total of 1 × 10^7^ to 2 × 10^7^ stained cells were analyzed on an LSRII flow cytometer (BD Biosciences). 7-AAD (BioLegend) was used to exclude dead cells and doublet cells were removed based on their forward/side scatter properties. CD45^–^CD326^–^ live cells were sorted into FACS buffer and kept on ice until the cells were further processed for scRNA-seq.

### scRNA-seq across time and conditions.

7-AAD^–^CD45^–^CD326^–^ flow sorted cells (100,000–300,000 cells) were collected and submitted for single-cell capture. Cellular suspensions were loaded on a Chromium Single-Cell Instrument (10× Genomics) to generate single-cell Gel Bead–in–Emulsions. scRNA-seq libraries were prepared using a Chromium Single-Cell 3′ Library & Gel Bead Kit (version 1.1; 10× Genomics). The beads were dissolved, and cells were lysed per the manufacturer’s recommendations. Libraries were sequenced using Illumina’s NovaSeq 6000 instrument. Cells (5000–6000) were captured per condition at a read depth of 50,000 reads per cell. The sequencing reads were examined by quality metrics, transcripts mapped to reference mouse genome (GRCm38), and assigned to individual cells according to cell barcodes, using Cell Ranger (10× Genomics).

### Identification of individual lung cell populations.

Data analysis was performed using R (version 4.1.3). Seurat 4.0 (50) was used for initial data analysis, normalization of gene expression, integration of samples, and identification and visualization of cell populations. Cell populations were identified based on gene markers and visualized by uniform manifold approximation and projection (UMAP) plots. Cell populations were identified by using the FindConservedMarkers function and then passing top cell type canonical marker genes to annotation databases, including Descartes Cell Types and Tissue 2021 (https://descartes.brotmanbaty.org), CellMarker Augmented 2021 (http://bio-bigdata.hrbmu.edu.cn/CellMarker), and Tabula Muris (https://www.czbiohub.org/sf/tabula-muris/) to identify likely cell types and then confirmed using a literature-based search of established marker genes for known pulmonary cell types. Supplemental Table 1 annotates the number of cells classified as each of the 15 canonical populations from the data set across the 6 conditions used. Reclustering was performed for the endothelial and mesenchymal compartments. Cell proportion bar plots were generated using the dittoSeq package. Further downstream analysis utilized the subset of endothelial and mesenchymal cells from the integrated analysis. The epithelial, immune, and mesothelial cells which were captured were excluded from analysis.

### Assessment of differentially expressed genes in TNF-mediated PH.

Differentially expressed genes were assessed between WT and TNF-Tg lungs in each cell type using the FindMarkers feature of Seurat. Filtering was performed to exclude genes which were expressed in less than 10% of cells in either condition. Differentially expressed genes were visualized using volcano plots generated using the EnhancedVolcano package.

### Assessment of pathways upregulated in TNF-Tg mice across all cell types.

To identify individuals genes that drive changes in transcription over the endothelial-mesenchymal axis in TNF-Tg lungs relative to control, we used the trajectory fitted by Slingshot and performed clustering, differential gene expression analysis, and visualization of genes that change significantly over pseudotime using the tradeSeq (51) package. Pathway analysis was performed using the enrichR package and the MSigDB Hallmark 2020 database (https://github.com/wjawaid/enrichR). Pathways with odds ratios of greater than 5 were considered and ordered by combined score (which incorporates both *P* values and odds ratios).

### Assessment of up- and downregulated pathways across relevant cell populations.

Pathway analysis was performed using the enrichR package and up- and downregulated genes for cell types and genes in differentially regulated regulons were assessed for pathway enrichment using (https://github.com/wjawaid/enrichR,) BioCarta_2016, ChEA_2022, GO_Biological_Process_2021, KEGG_2019_Mouse, Panther_2016, Reactome_2022, and WikiPathways_2019_Mouse databases. Pathways with odds ratios of greater than 10 were considered and then top pathways were visualized by a Cleveland dot plot to demonstrate top pathways with the lowest *P* values on the left and sequentially decreasing significance.

### Assessment of transcription factor binding in WT and TNF-Tg lungs.

The SCENIC (13) package was used to identify gene coexpression modules (regulons) controlled by common transcription factor binding sites using *cis*-regulatory analysis. Briefly, this method builds a gene regulatory network (GRN) by first identifying potential targets for each transcription factor based on coexpression. We identified coexpressed genes using GENIE3 to infer potential transcription factor targets based on the expression data and SCENIC wrapper functions in R to build the gene regulatory network, obtain coexpression modules, identify regulons and cell states, score regulons in the cells with AUCell, and clustering cells according to the gene regulatory network activity. Cell-type-specific regulators based on the RSS were identified and plotted using the rssplot function. We then identified stable cell states based on their t-SNE clustering and explored the results using the web based utility SCope (https://scope.aertslab.org/) for visualization of gene and regulon coexpression.

### RNA trajectory, pseudotime, and velocity.

To assess cells’ transition from one functional state to another we assessed single-cell trajectories using 3 different approaches. Monocle3 (52) and Slingshot (53) were used to assess trajectory and pseudotime for RNA transcription. Monocle3 first performs dimension reduction with UMAP, and then it clusters the cells with Louvain/Leiden algorithms on a k-nearest neighbor graph to connect adjacent groups and resolves the trajectories of individual cells utilizing a learning principal graph. In contrast, Slingshot first performs inference of the global lineage structure using MST on clustered data points and then inferers pseudotime variables for cells along each lineage by fitting simultaneous principal curves across multiple lineages.

RNA velocity is a newer and more sensitive method for identifying directed information by distinguishing unspliced transcripts from mature mRNAs (spliced). To assess RNA velocity, raw sequence reads were processed in a Linux environment using Samtools to filter and sort reads and the velocyto (54) command line tool to determine spliced and unspliced transcripts. The resulting LOOM files were analyzed in the python implementation of scVelo to perform velocity analysis. scVelo (14) was used to convert spliced and unspliced reads into cell × gene matrices, processes these matrices to generate phase plots describing a dynamical transcription process, and fits transcriptional dynamics. This allows for display of velocity in a low-dimensional embedding (UMAP space) with the ability to calculate both the length and certainty of the velocity vectors and assess velocity pseudotime. Finally, to ascertain cellular fate decisions, the Cellrank (15) package was used to generate a PAGA plot where directed edges reflect local velocity compute_flow as well as pseudotemporal ordering to ascertain the starting cell type and restrict the possible ending states.

### Ligand-receptor interaction analysis.

To assess individual ligand-receptor interactions across the full range of conditions, we employed the MultiNicheNet package. MultiNicheNet is an implementation of NicheNet (16) that enables assessment of differential cell-cell communication events (ligand-receptor interactions and downstream signaling to target genes) based on upregulation of the ligand in a sender cell type and/or upregulation of the receptor in a receiver cell type, high expression of ligand and receptor in many samples of the same group, cell-type- and condition-specific expression of the ligand in the sender cell type and receptor in the receiver cell type, and high NicheNet ligand activity, to further prioritize ligand-receptor pairs based on their predicted effect of the ligand-receptor interaction on the gene expression in the receiver cell type. The algorithm utilizes pseduobulk expression for each sample scored used the muscat framework. MultiNicheNet combines all these criteria in a single prioritization score, which is also comparable between all sender-receiver pairs and the top 40 most highly prioritized up- and downregulated L:R pairs were mapped. Circos plots were generated in WT and TNF conditions indicating cell-specific ligand interactions. Systems network interactions were calculated using the ggraph_signaling_plot function to draw links between ligands of sender cell types and ligand/receptor-annotated target genes in receiver cells.

### Immunostaining.

Fluorescent immunohistology was performed on histological sections of WT and TNF lung tissues at 8 and 20 weeks (*n* = 5 per genotype per time point). Formalin-fixed paraffin sections (5 μm) were incubated at 60°C overnight. Tissue sections were quickly transferred to xylene and gradually hydrated by transferring slides to absolute alcohol, 96% alcohol, 70% alcohol, and water. Slides were immersed in an antigen retrieval solution, boiled for 30 minutes, and cooled down for 10 minutes at room temperature (RT). Slides were rinsed several times in water and transferred to PBS. Nonspecific binding was blocked with 5% normal donkey serum in PBS containing 0.1% Tween 20 and 0.1% Triton X-100 for 30 minutes, at RT in a humid chamber. Primary antibodies were added to slides and incubated in a humid chamber at room RT overnight. Slides were quickly washed in PBS and incubated with fluorescently labeled secondary antibodies for 2 hours at RT in a humid chamber. Finally, slides were rinsed for 1 hour in PBS and mounted with Vectashield antifade mounting media with DAPI (H-1200, Vector Laboratories). Pictures were acquired with a Zeiss Axioplan 2 microscope and recorded with a Hamamatsu camera.

Primary antibodies used included BMPR2 (rabbit, PA5-95839, Thermo Fisher Scientific), NOG (sheep, PA5-47807, Thermo Fisher Scientific), α-SMA (mouse, clone 1A4, MS-113-B, Thermo Fisher Scientific), BMP2 (rabbit, MBS249851, MyBioSource), BMP4 (rabbit, GTX100875, GeneTex), BMPR1 (rabbit, ABIN200094, Antibodies Online; https://www.antibodies-online.com/), HJV (goat, AF3720, R&D Systems), IGFBP7 (rabbit, PA5-76685, Thermo Fisher Scientific), COL4A1 (goat, NBP1-26549, Novus Biologicals), and ITGA2 (rat, 108901, BioLegend).

Secondary antibodies included Cy3 donkey anti-sheep IgG (713-166-147, Jackson ImmunoResearch Laboratories), Alexa Fluor 488 donkey anti-rabbit IgG (711-546-152, Jackson ImmunoResearch Laboratories), Cy3 donkey anti-goat IgG (705-166-147, Jackson ImmunoResearch Laboratories), and Alexa Fluor 647 donkey anti-rat IgG (712-606-153, Jackson ImmunoResearch Laboratories).

### Morphometric analysis.

Two to 3 random pictures (original magnification, ×200) per sample were taken with a Hamamatsu camera in pulmonary areas with blood vessels or bronchi. Areas covered by NOG, BMP2, or BMPR2 signals were quantified with ImageJ (NIH). Areas in squared pixels were transformed to square microns. Graphs represent the average and SEM.

### Network-based cell-cell communication.

Intercellular communication can be ascertained from scRNA-seq data; to do this, we utilized the CellChat (17) package to analyze cell-cell communication for continuous states along cellular developmental trajectories. This framework enables quantitative characterization of cellular interactions across cell types and comparisons of the inferred cell-cell communication networks using an integrated approach by combining social network analysis, pattern recognition, and manifold learning approaches. Specifically, we utilized this package to identify and visualize differential numbers and strengths of interactions across cell type, identify and characterize cellular communication patterns, and to ascertain differential pathway expression of ligand-receptor interactions between cells using literature annotations from the CellChat database.

### Cell isolation.

Mouse lungs were perfused through the left ventricle with HBSS containing 1000 U/mL heparin. Lungs were cut into small pieces and incubated with collagenase II (1000 U/mL), Dispase (2.5 U/mL) (Worthington Biochem), DNase I (10 mg/mL; Sigma-Aldrich), and CaCl_2_ (250 mM) (Sigma-Aldrich) at 37°C for 45 minutes. Lung pieces were passed through an insulin syringe and filtered in a sterile 100-μm cell strainer. Cell suspensions were centrifuged at 350*g* for 8 minutes and washed twice with PBS (Thermo Fisher Scientific). Cell suspensions were incubated in PBS containing 2% FBS (Sigma-Aldrich) and 2 μg/mL FcBlock (2.4G2, BioXcell) for 10 minutes on ice. Endothelial cells were isolated using Invitrogen Dynabeads CD31 Endothelial Cells and a magnetic particle concentrator according to the manufacturers protocol (Thermo Fisher Scientific). CD31^+^ cells were cultured on plates covered with fibronectin (10 mg/mL) and endothelial cell media (M1168, Cell Biologics) supplemented with 5% heat-inactivated FBS, 0.5 mL VEGF, ECGS, heparin, EGF, hydrocortisone, and antibiotic-antimycotic solution as provided by the manufacturer. Culture media were replaced every other day and CD31^+^ cells were purified 3 times before cytokine stimulation. For fibroblast culture, endothelial cells (anti-CD31; 390, BioLegend), leukocytes (anti-CD45; 30-F11, BioLegend), smooth muscle cells (anti-ITGA8: BAF4076, R&D Systems), pericytes (anti-CD140; APB5, BioLegend), and epithelial cells (anti–E-cadherin: BAF748, R&D Systems) were depleted by positive selection on LS+ columns (Miltenyi Biotec) using biotin-labeled antibodies (BD Biosciences) and streptavidin coupled to magnetic microbeads according to the manufacturer’s protocol. Fibroblasts were cultured in DMEM (GIBCO) supplemented with 20% heat-inactivated FBS (Sigma-Aldrich), 1× GlutaMax, and antibiotic-antimycotic solution. For the cell culture transfer experiment, endothelial cells or fibroblasts were cultured in complete endothelial cell culture or DMEM with or without 20 mg/mL human TNF (300-01A, Peprotech) for 24 hours. Cells were washed 5 times with PBS and cultured for 72 hours in serum-free endothelial or fibroblast media. Supernatants were collected and centrifuged at 485*g* for 5 minutes. Two million endothelial cells were cultured with fibroblast-conditioned media and 2 million fibroblasts were cultured with conditioned media from endothelial cells for 6 hours. Cells were harvested and RNA was isolated for gene expression analysis.

### Cell stimulations.

Lung fibroblasts and endothelial cells were stimulated with IL-1B (211-11B, Thermo Fisher Scientific), IL-6 (216-16, Thermo Fisher Scientific), CD40L (315-15, Thermo Fisher Scientific), and LTβ (LS-G13992, LSbio) for 3, 6 and 24 hours.

### Quantitative PCR.

Cells were lysed with TRizol (15596026, Thermo Fisher Scientific) and glycogen (R0551, Thermo Fisher Scientific) was used as a carrier to improve RNA isolation. RNA (2 mg) was reverse transcribed with a High Capacity cDNA Reverse Transcription kit (4368814, Thermo Fisher Scientific). cDNA concentration was adjusted to 10 ng/mL. Quantitative PCR reactions were performed with 10 ng of cDNA and iTaq Universal SYBR Green supermix (1725124, Bio-Rad). Gene expression was normalized to expression of the *18s* housekeeping gene and levels of gene expression in stimulated cells from TNF-Tg and C57BL/6 mice were compared to the gene expression in unstimulated cells of C57BL/6 mice.

### Western blot.

Total proteins from mouse lung endothelial cells and fibroblasts stimulated with IL-1B, IL-6, CD40L, and LTβ were extracted with CytoBuster buffer containing 1× protease inhibitor cocktail (Thermo Fisher Scientific) according to the manufacturer’s instructions. Protein was quantified using a micro bicinchoninic acid protein assay kit (Thermo Fisher Scientific). One volume of NuPAGE sample buffer (Thermo Fisher Scientific) was added to 15 μg of protein lysate and boiled for 5 minutes at 95°C. Protein samples were resolved in 4%–12% Bis-Tris Plus Bolt precast mini gels and transferred onto an iBlot 2 PVDF stack using an iBLot electrophoretic transfer unit (Thermo Fisher Scientific). PVDF membranes were blocked with 5% blotting grade blocker (Bio-Rad) at RT in PBS containing 0.05% Tween 20 (Sigma-Aldrich) at RT for 1 hour and incubated with primary antibodies against β-actin (PA1-183, Invitrogen), Smad1 (6944S, Cell Signaling Technology), and Smad2/3 (3102S, Cell Signaling Technology) overnight at 4°C. Bound antibodies were detected with HPR-conjugated rabbit antibodies (NA934V, MilliporeSigma) and probed by the SuperSignal chemiluminescence system (Thermo Fisher Scientific).

### Statistics.

Differential gene expression analysis between cells or groups of cells within clusters were performed using Seurat’s implementation of the nonparametric Wilcoxon’s rank-sum test (50). A Bonferroni correction was applied to the results. Genes differentially expressed along pseudotime lineages were calculated using tradeSeq (51). Prioritization scoring for ligand-receptor pairs was performed with expression values using the differential NicheNet algorithm (16). For comparisons between cell population frequency, scProportionTest (55) was utilized. For comparisons between 2 groups, statistical significance was calculated with a 2-tailed Student’s *t* test. A *P* value of less than 0.05 was considered significant.

### Study approval.

All animal work was approved by the University of Rochester (Rochester, New York, USA) University Committee on Animal Resources (UCAR) under the protocol number 102056.

### Data availability.

The raw data from the scRNA-seq have been deposited in the NCBI Gene Expression Omnibus (GEO) under accession number GSE279244. Values for all data points in graphs are reported in the Supporting Data Values file. Additional processed data and image files are available upon request.

## Author contributions

MLGH and JRM performed the immunostaining, in vitro isolation and stimulation experiments, and performed the bulk of the data analysis and writing. QX performed the flow isolation of lung cell and preparation of cells for scRNA-seq. YJ and RM performed cell isolation, culture, imaging, and data analysis. SD maintained detailed experimental logs, obtained tissue from animals, and performed imaging. SB and BDK performed multiple bioinformatic analysis. BDK conceived of the presented idea and designed the analysis plan. BDK and KY supervised the findings of this work and oversaw the writing of the manuscript. All authors discussed the results and contributed to the final manuscript.

## Supplementary Material

Supplemental data

Unedited blot and gel images

Supporting data values

## Version 1 06/26/2025

In-Press Preview

## Version 2 07/22/2025

Electronic publication
