# Supplementary material for: TNF drives aberrant BMP signaling to induce endothelial and mesenchymal dysregulation in pulmonary hypertension
Source: JCI Insight. 2025 Jun 26;10(14):e174456. doi: 10.1172/jci.insight.174456 (PMC12288976; doi:10.1172/jci.insight.174456)
Supplement: Supplemental data [file jciinsight-10-174456-s133.pdf]

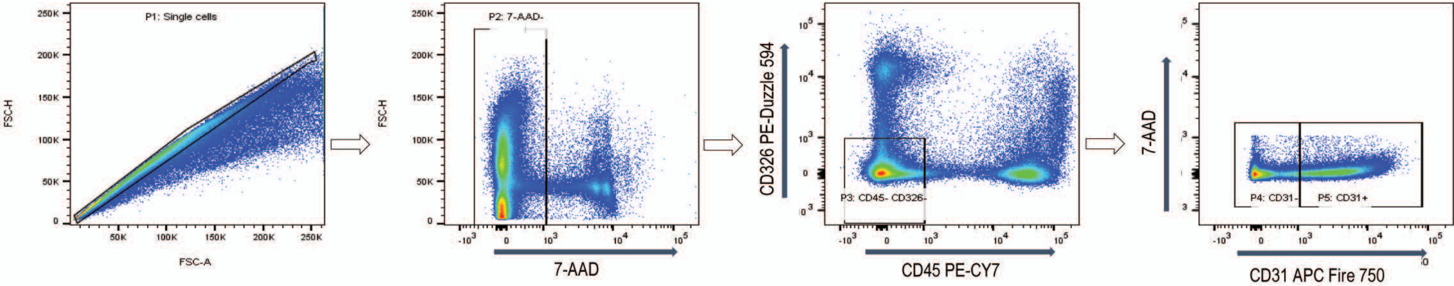
P1: Single cells
FSC-H
FSC-A
P2: 7-AAD-
FSC-H
7-AAD
CD326 PE-Duzzle 594
P3: CD45- CD326-
CD45 PE-CY7
7-AAD
P4: CD31-
P5: CD31+
CD31 APC Fire 750

Supplemental Figure 1. Gating strategy to generate non-hematopoietic, non-epithelial pulmonary single cells by flow cytometry. Gates shown include single cells (P1), viable cells (P2), non-epithelial cells ($CD326^-$) and non-leukocyte ($CD45^-$) cells (P3), CD31- (P4) and CD31+ (P5).

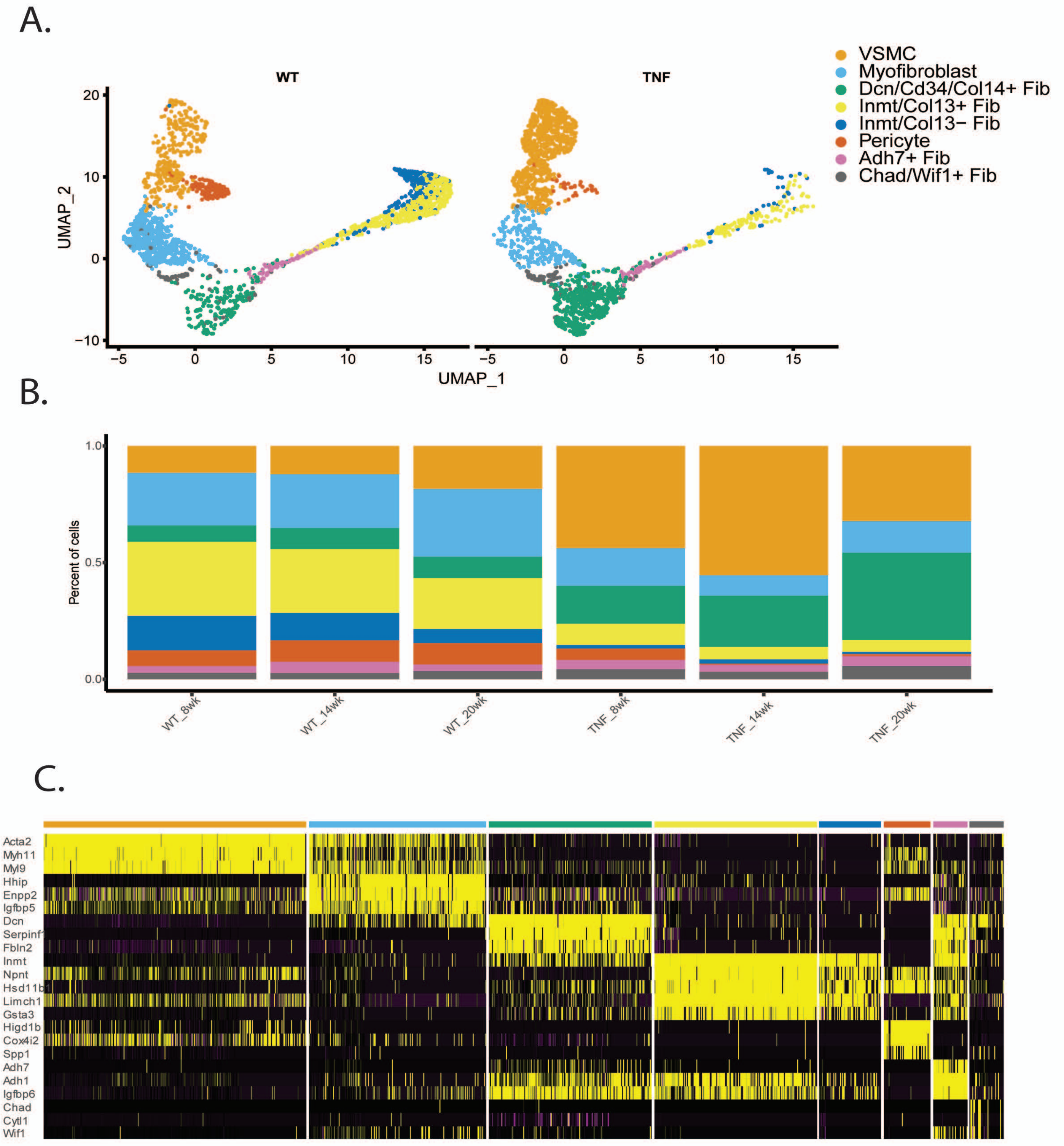
A.
WT
TNF
VSMC
Myofibroblast
Dcn/Cd34/Col14+ Fib
Inmt/Col13+ Fib
Inmt/Col13− Fib
Pericyte
Adh7+ Fib
Chad/Wif1+ Fib
UMAP_2
UMAP_1
20
10
0
−10
−5
0
5
10
15
B.
Percent of cells
1.0
0.5
0.0
WT_8wk
WT_14wk
WT_20wk
TNF_8wk
TNF_14wk
TNF_20wk
C.
Acta2
Myh11
Myl9
Hhip
Enpp2
Igfbp5
Dcn
Serpinf1
Fbln2
Inmt
Npnt
Hsd11b1
Limch1
Gsta3
Higd1b
Cox4i2
Spp1
Adh7
Adh1
Igfbp6
Chad
Cytl1
Wif1

Supplemental Figure 2. Reclustering of mesenchymal cells identifies additional fibroblast subsets. A. UMAP projection of resclustering of mesenchymal cells identifies that Col14+ fibroblasts include both DCN/CD34/Col14^{+} cells and Chad/Wif1^{+} fibroblast sub-populations while Col13+ fibroblasts are made of clusters including Inmt^{+}/Col13^{+}, Inmt^{+}/Col13^{-}, and Adh7^{+} sub-populations. B. Stacked bar plots indicating the relative proportion of cells of each mesenchymal subtype in each condition. C. Heatmap indicating top 3 genes differentiating each cluster.

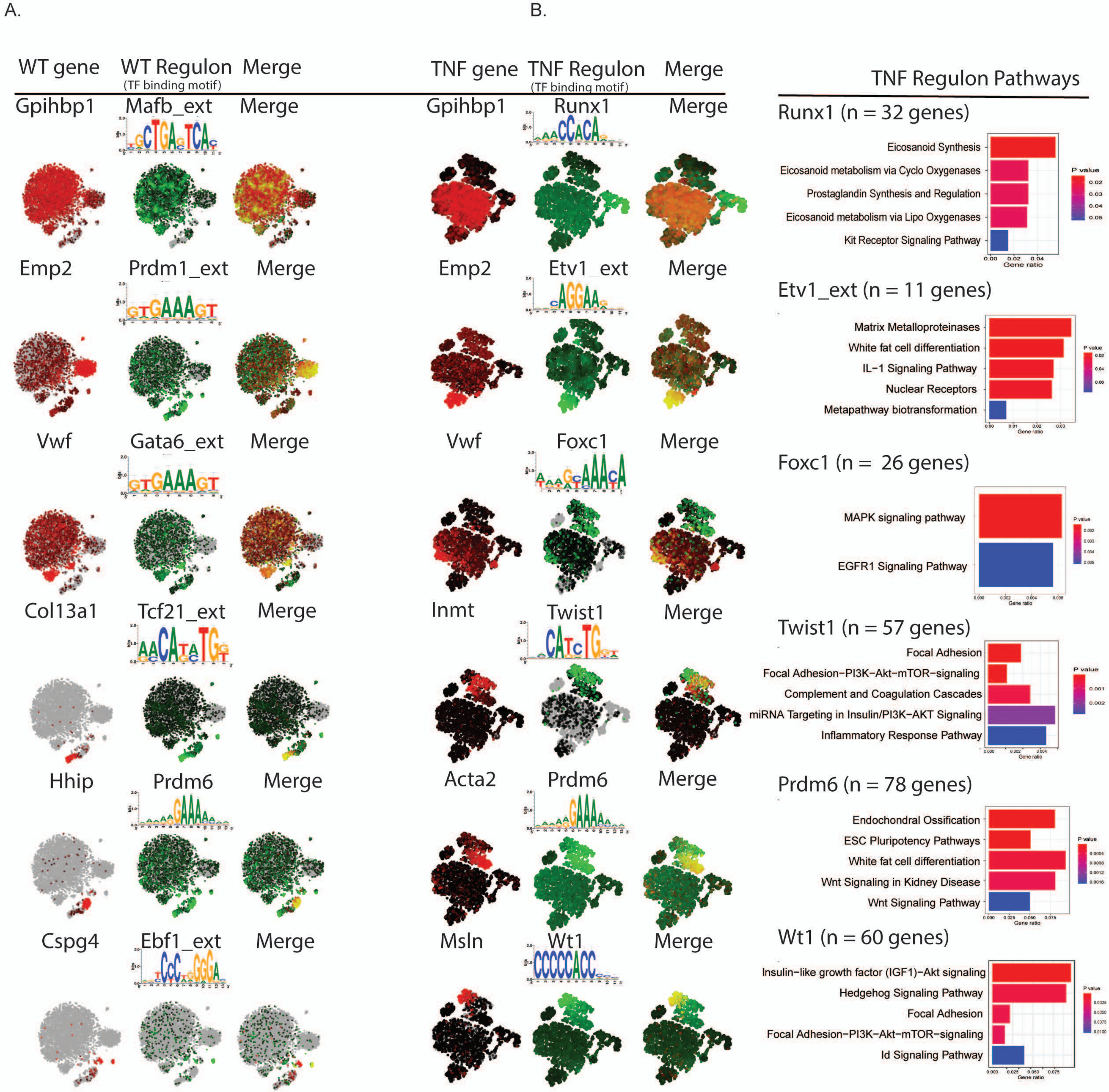

A.
B.
WT gene
WT Regulon
(TF binding motif)
Merge
TNF gene
TNF Regulon
(TF binding motif)
Merge
TNF Regulon Pathways
Gpihbp1
Mafb_ext
Merge
Gpihbp1
Runx1
Merge
Runx1 (n = 32 genes)
Eicosanoid Synthesis
Eicosanoid metabolism via Cyclo Oxygenases
Prostaglandin Synthesis and Regulation
Eicosanoid metabolism via Lipo Oxygenases
Kit Receptor Signaling Pathway
Gene ratio
P value
Emp2
Prdm1_ext
Merge
Emp2
Etv1_ext
Merge
Etv1_ext (n = 11 genes)
Matrix Metalloproteinases
White fat cell differentiation
IL−1 Signaling Pathway
Nuclear Receptors
Metapathway biotransformation
Vwf
Gata6_ext
Merge
Vwf
Foxc1
Merge
Foxc1 (n = 26 genes)
MAPK signaling pathway
EGFR1 Signaling Pathway
Col13a1
Tcf21_ext
Merge
Inmt
Twist1
Merge
Twist1 (n = 57 genes)
Focal Adhesion
Focal Adhesion−PI3K−Akt−mTOR−signaling
Complement and Coagulation Cascades
miRNA Targeting in Insulin/PI3K−AKT Signaling
Inflammatory Response Pathway
Hhip
Prdm6
Merge
Acta2
Prdm6
Merge
Prdm6 (n = 78 genes)
Endochondral Ossification
ESC Pluripotency Pathways
White fat cell differentiation
Wnt Signaling in Kidney Disease
Wnt Signaling Pathway
Cspg4
Ebf1_ext
Merge
Msln
Wt1
Merge
Wt1 (n = 60 genes)
Insulin−like growth factor (IGF1)−Akt signaling
Hedgehog Signaling Pathway
Focal Adhesion
Focal Adhesion−PI3K−Akt−mTOR−signaling
Id Signaling Pathway

Supplemental Figure 3. Identification of altered gene regulation and transcription factor binding in TNF-Tg cells using the SCENIC pipeline. A. t-SNE plots indicate the distribution of expression of markers of cell populations (red, left), regulons (green, center), and merged (right, yellow indicating co-expression of the cell identity marker and the regulon) for six regulons which are present predominantly in WT (left panel) and only in TNF (right panel). Each regulon is characterized by transcription factor binding indicated by the name of the regulon and the binding motif indicated under this name. B. Differentially regulated pathways defined by the genes in each of the regulons which were found in TNF-Tg but not WT lungs

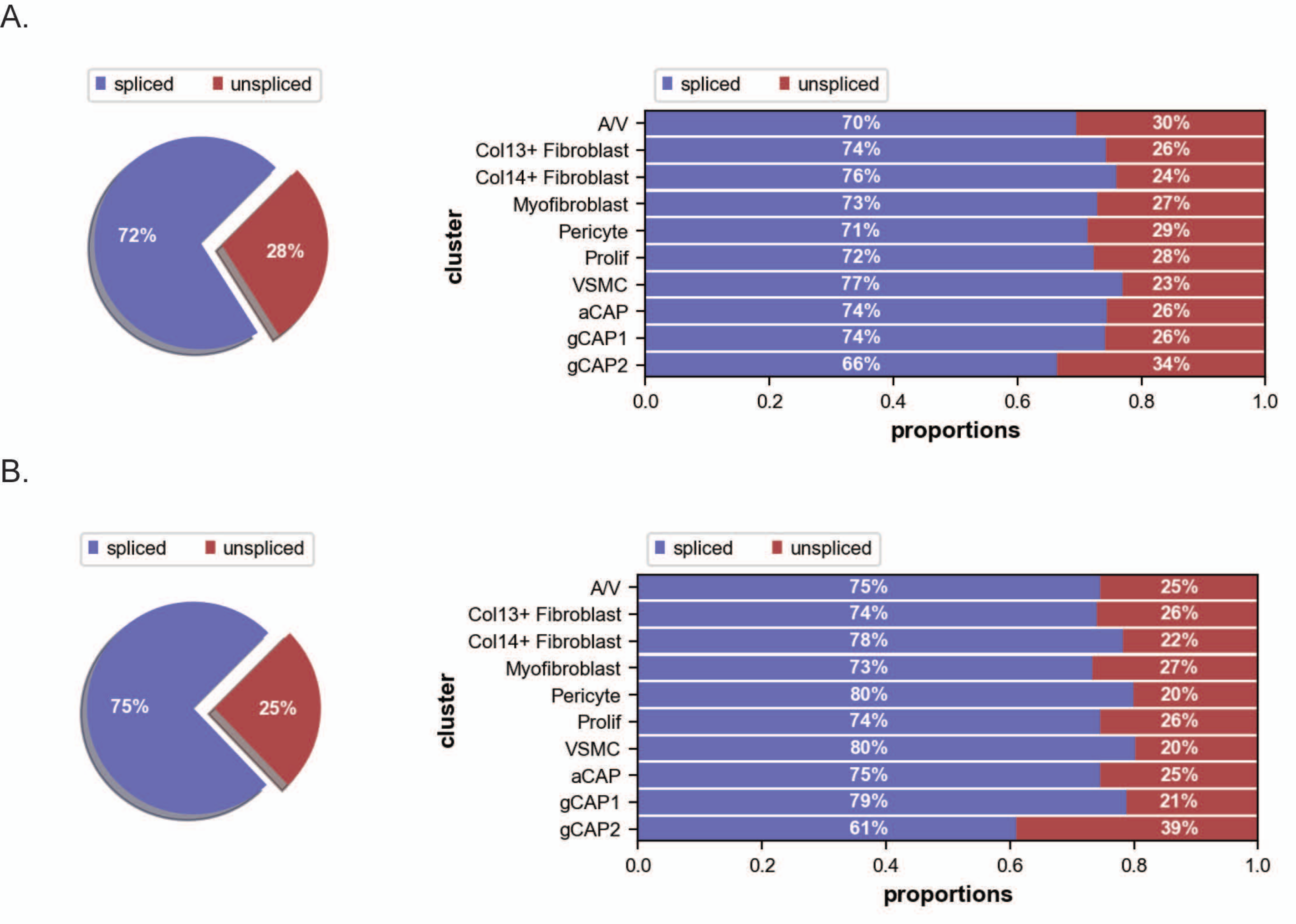
A.
spliced
unspliced
72%
28%
spliced
unspliced
A/V
Col13+ Fibroblast
Col14+ Fibroblast
Myofibroblast
Pericyte
Prolif
VSMC
aCAP
gCAP1
gCAP2
70% 30%
74% 26%
76% 24%
73% 27%
71% 29%
72% 28%
77% 23%
74% 26%
74% 26%
66% 34%
cluster
0.0
0.2
0.4
0.6
0.8
1.0
proportions
B.
spliced
unspliced
75%
25%
spliced
unspliced
A/V
Col13+ Fibroblast
Col14+ Fibroblast
Myofibroblast
Pericyte
Prolif
VSMC
aCAP
gCAP1
gCAP2
75% 25%
74% 26%
78% 22%
73% 27%
80% 20%
74% 26%
80% 20%
75% 25%
79% 21%
61% 39%
cluster
0.0
0.2
0.4
0.6
0.8
1.0
proportions

Supplemental Figure 4. Differential mRNA splicing across condition and cell types. Percentage of spliced and unspliced transcripts calculated by velocyto in A. WT and B. TNF-Tg lungs. All cells are shown in pie charts (left) while proportions of splicing in each cellular subset are shown in bar charts (right).

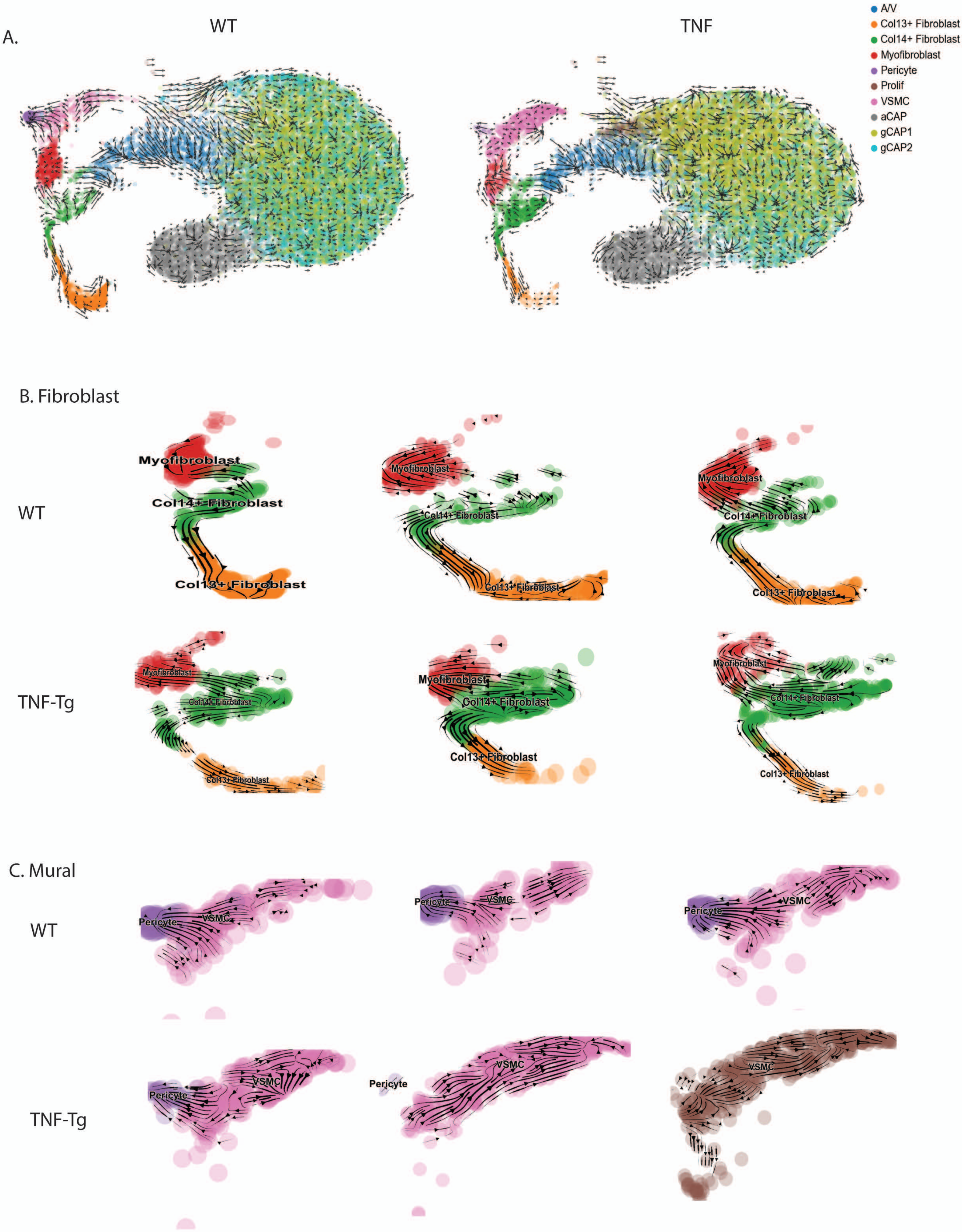
A.
WT
TNF
A/V
Col13+ Fibroblast
Col14+ Fibroblast
Myofibroblast
Pericyte
Prolif
VSMC
aCAP
gCAP1
gCAP2
B. Fibroblast
WT
TNF-Tg
Myofibroblast
Col14+ Fibroblast
Col13+ Fibroblast
C. Mural
WT
TNF-Tg
Pericyte
VSMC

Supplemental Figure 5. RNA velocity in endothelial, fibroblast, and mural cell subset over time. Velocities were projected onto UMAP embeddings with arrows indicating direction and relative velocity were generated in A. endothelial B. fibroblast and C. mural cells using scVelo. In each cell condition, separate velocity embeddings were generated for WT (upper) and TNF-Tg (lower) conditions at 8 weeks (left), 14 weeks (center), and 20 weeks (right).

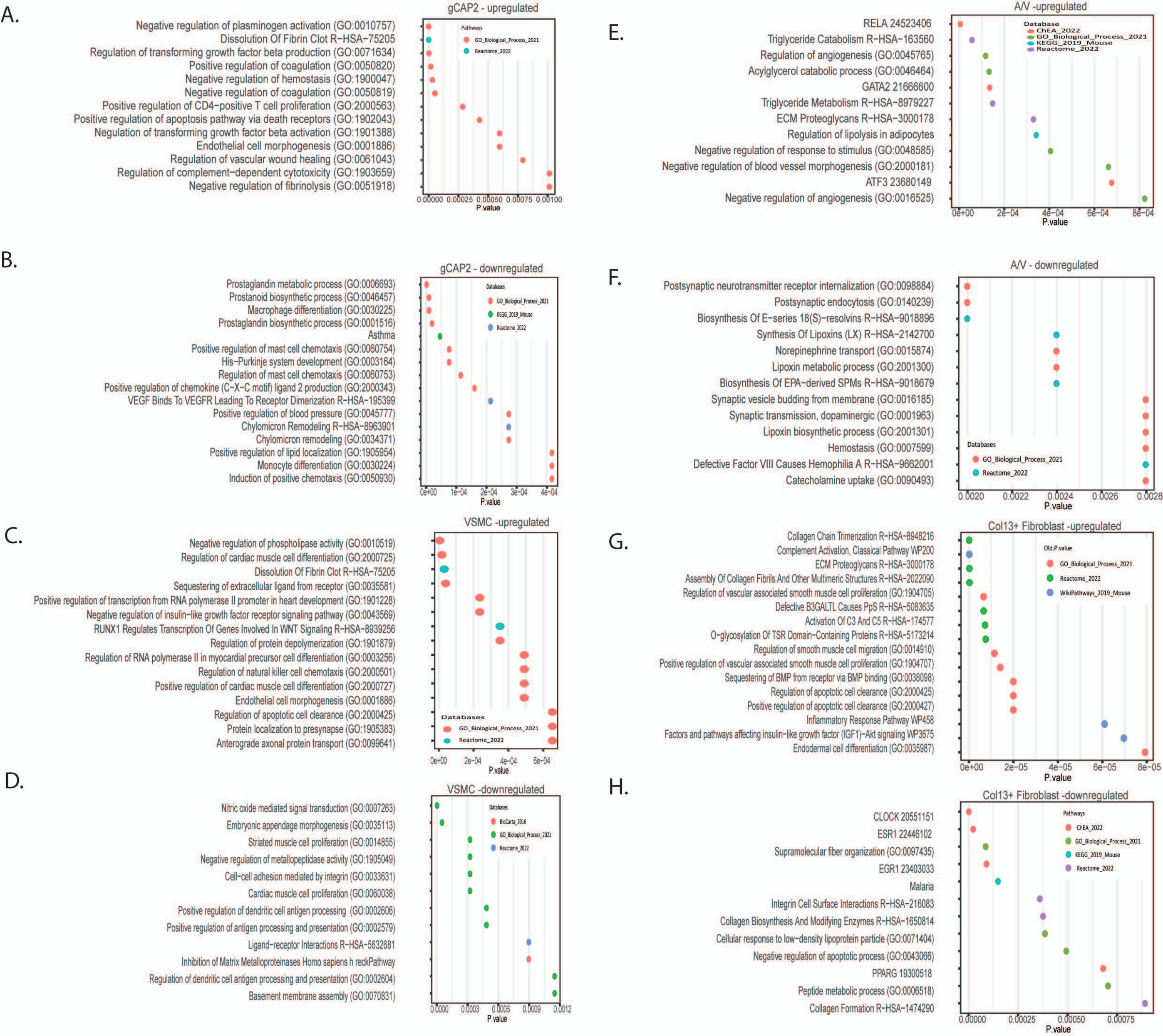
A.
gCAP2 - upregulated
Negative regulation of plasminogen activation (GO:0010757)
Dissolution Of Fibrin Clot R-HSA-75205
Regulation of transforming growth factor beta production (GO:0071634)
Positive regulation of coagulation (GO:0050820)
Negative regulation of hemostasis (GO:1900047)
Negative regulation of coagulation (GO:0050819)
Positive regulation of CD4-positive T cell proliferation (GO:2000563)
Positive regulation of apoptosis pathway via death receptors (GO:1902043)
Regulation of transforming growth factor beta activation (GO:1901388)
Endothelial cell morphogenesis (GO:0001886)
Regulation of vascular wound healing (GO:0061043)
Regulation of complement-dependent cytotoxicity (GO:1903659)
Negative regulation of fibrinolysis (GO:0051918)
Pathways
GO_Biological_Process_2021
Reactome_2022
0.00000 0.00025 0.00050 0.00075 0.00100
P.value
B.
gCAP2 - downregulated
Prostaglandin metabolic process (GO:0006693)
Prostanoid biosynthetic process (GO:0046457)
Macrophage differentiation (GO:0030225)
Prostaglandin biosynthetic process (GO:0001516)
Asthma
Positive regulation of mast cell chemotaxis (GO:0060754)
His-Purkinje system development (GO:0003164)
Regulation of mast cell chemotaxis (GO:0060753)
Positive regulation of chemokine (C-X-C motif) ligand 2 production (GO:2000343)
VEGF Binds To VEGFR Leading To Receptor Dimerization R-HSA-195399
Positive regulation of blood pressure (GO:0045777)
Chylomicron Remodeling R-HSA-8963901
Chylomicron remodeling (GO:0034371)
Positive regulation of lipid localization (GO:1905954)
Monocyte differentiation (GO:0030224)
Induction of positive chemotaxis (GO:0050930)
Databases
GO_Biological_Process_2021
KEGG_2019_Mouse
Reactome_2022
0e+00 1e-04 2e-04 3e-04 4e-04
P.value
C.
VSMC -upregulated
Negative regulation of phospholipase activity (GO:0010519)
Regulation of cardiac muscle cell differentiation (GO:2000725)
Dissolution Of Fibrin Clot R-HSA-75205
Sequestering of extracellular ligand from receptor (GO:0035581)
Positive regulation of transcription from RNA polymerase II promoter in heart development (GO:1901228)
Negative regulation of insulin-like growth factor receptor signaling pathway (GO:0043569)
RUNX1 Regulates Transcription Of Genes Involved In WNT Signaling R-HSA-8939256
Regulation of protein depolymerization (GO:1901879)
Regulation of RNA polymerase II in myocardial precursor cell differentiation (GO:0003256)
Regulation of natural killer cell chemotaxis (GO:2000501)
Positive regulation of cardiac muscle cell differentiation (GO:2000727)
Endothelial cell morphogenesis (GO:0001886)
Regulation of apoptotic cell clearance (GO:2000425)
Protein localization to presynapse (GO:1905383)
Anterograde axonal protein transport (GO:0099641)
Databases
GO_Biological_Process_2021
Reactome_2022
0e+00 2e-04 4e-04 6e-04
P.value
D.
VSMC -downregulated
Nitric oxide mediated signal transduction (GO:0007263)
Embryonic appendage morphogenesis (GO:0035113)
Striated muscle cell proliferation (GO:0014855)
Negative regulation of metallopeptidase activity (GO:1905049)
Cell-cell adhesion mediated by integrin (GO:0033631)
Cardiac muscle cell proliferation (GO:0060038)
Positive regulation of dendritic cell antigen processing (GO:0002606)
Positive regulation of antigen processing and presentation (GO:0002579)
Ligand-receptor Interactions R-HSA-5632681
Inhibition of Matrix Metalloproteinases Homo sapiens h reckPathway
Regulation of dendritic cell antigen processing and presentation (GO:0002604)
Basement membrane assembly (GO:0070831)
Databases
BioCarta_2016
GO_Biological_Process_2021
Reactome_2022
0.0000 0.0003 0.0006 0.0009 0.0012
P.value
E.
A/V -upregulated
RELA 24523406
Triglyceride Catabolism R-HSA-163560
Regulation of angiogenesis (GO:0045765)
Acylglycerol catabolic process (GO:0046464)
GATA2 21666600
Triglyceride Metabolism R-HSA-8979227
ECM Proteoglycans R-HSA-3000178
Regulation of lipolysis in adipocytes
Negative regulation of response to stimulus (GO:0048585)
Negative regulation of blood vessel morphogenesis (GO:2000181)
ATF3 23680149
Negative regulation of angiogenesis (GO:0016525)
Database
ChEA_2022
GO_Biological_Process_2021
KEGG_2019_Mouse
Reactome_2022
0e+00 2e-04 4e-04 6e-04 8e-04
P.value
F.
A/V - downregulated
Postsynaptic neurotransmitter receptor internalization (GO:0098884)
Postsynaptic endocytosis (GO:0140239)
Biosynthesis Of E-series 18(S)-resolvins R-HSA-9018896
Synthesis Of Lipoxins (LX) R-HSA-2142700
Norepinephrine transport (GO:0015874)
Lipoxin metabolic process (GO:2001300)
Biosynthesis Of EPA-derived SPMs R-HSA-9018679
Synaptic vesicle budding from membrane (GO:0016185)
Synaptic transmission, dopaminergic (GO:0001963)
Lipoxin biosynthetic process (GO:2001301)
Hemostasis (GO:0007599)
Defective Factor VIII Causes Hemophilia A R-HSA-9662001
Catecholamine uptake (GO:0090493)
Databases
GO_Biological_Process_2021
Reactome_2022
0.0020 0.0022 0.0024 0.0026 0.0028
P.value
G.
Col13+ Fibroblast -upregulated
Collagen Chain Trimerization R-HSA-8948216
Complement Activation, Classical Pathway WP200
ECM Proteoglycans R-HSA-3000178
Assembly Of Collagen Fibrils And Other Multimeric Structures R-HSA-2022090
Regulation of vascular associated smooth muscle cell proliferation (GO:1904705)
Defective B3GALTL Causes PpS R-HSA-5083635
Activation Of C3 And C5 R-HSA-174577
O-glycosylation Of TSR Domain-Containing Proteins R-HSA-5173214
Regulation of smooth muscle cell migration (GO:0014910)
Positive regulation of vascular associated smooth muscle cell proliferation (GO:1904707)
Sequestering of BMP from receptor via BMP binding (GO:0038098)
Regulation of apoptotic cell clearance (GO:2000425)
Positive regulation of apoptotic cell clearance (GO:2000427)
Inflammatory Response Pathway WP458
Factors and pathways affecting insulin-like growth factor (IGF1)-Akt signaling WP3675
Endodermal cell differentiation (GO:0035987)
Old.P.value
GO_Biological_Process_2021
Reactome_2022
WikiPathways_2019_Mouse
0e+00 2e-05 4e-05 6e-05 8e-05
P.value
H.
Col13+ Fibroblast -downregulated
CLOCK 20551151
ESR1 22446102
Supramolecular fiber organization (GO:0097435)
EGR1 23403033
Malaria
Integrin Cell Surface Interactions R-HSA-216083
Collagen Biosynthesis And Modifying Enzymes R-HSA-1650814
Cellular response to low-density lipoprotein particle (GO:0071404)
Negative regulation of apoptotic process (GO:0043066)
PPARG 19300518
Peptide metabolic process (GO:0006518)
Collagen Formation R-HSA-1474290
Pathways
ChEA_2022
GO_Biological_Process_2021
KEGG_2019_Mouse
Reactome_2022
0.00000 0.00025 0.00050 0.00075
P.value

Supplemental Figure 6. Up-regulated and down-regulated pathways in selected cell types. More granular pathway analysis of cells from volcano plots in Figure 4 was performed in (A-B) gCAP2 cells, (C-D) vascular smooth muscle cells, (E-F) arterial/venous endothelial cells, and (G-H) collagen 13+ fibroblasts using the enrichR package. Up-regulated (A,C,E,G) and down-regulated (B,D,F,H) pathways in TNF vs WT cells are demonstrated by Cleveland dot plots with colors indicating the database where pathway annotation was derived and x-axis indicating significance (p-value) of over-representation of each specified pathway

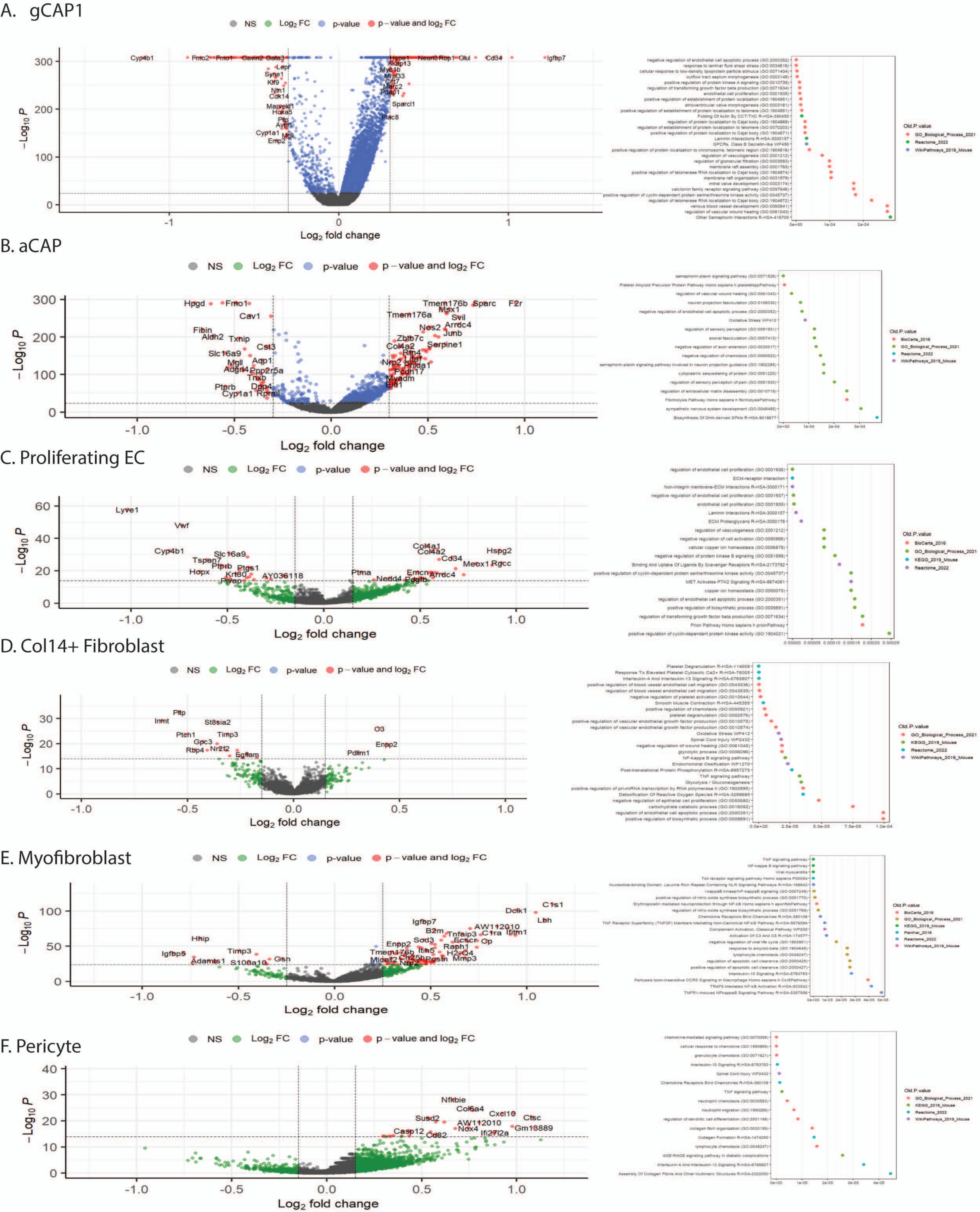
A. gCAP1
NS Log2 FC p-value p – value and log2 FC
Cyp4b1 Fmo2 Fmo1 Cavin2 Gata3 Hspe1 Neurl3 Rbp1 Glul Cd34 Igfbp7
Lepr Syne1 Klf9 Ntn1 Cdk14 Marveld1 Hoxa5 Pltp Avpr1 Cyp1a1 Mgll Emp2
Akap13 Myo1b Mrpl33 Cct7 Marc2 Pdap1 Sparcl1 Plac8
−Log10 P
Log2 fold change
Old.P.value
GO_Biological_Process_2021
Reactome_2022
WikiPathways_2019_Mouse
B. aCAP
NS Log2 FC p-value p – value and log2 FC
Hpgd Fmo1 Cav1 Fibin Aldh2 Txnip Cst3 Slc16a9 Mgll Aqp1 Adgrl4 Ppp2r5a Tnxb Ptprb Dpp4 Cyp1a1 Rprml
Tmem176b Sparc F2r Msx1 Tmem176a Svil Arrdc4 Nos2 Junb Zbtb7c Col4a2 Serpine1 Rtn4 Nrp2 Litaf Phlda1 Pcdh17 Myadm Ehd1
−Log10 P
Log2 fold change
Old.P.value
BioCarta_2016
GO_Biological_Process_2021
Reactome_2022
WikiPathways_2019_Mouse
C. Proliferating EC
NS Log2 FC p-value p – value and log2 FC
Lyve1 Vwf Cyp4b1 Slc16a9 Tspan7 Ptprb Ptgs1 Hopx Krt80 Plvap AY036118
Ptma Nedd4 Col4a1 Col4a2 Cd34 Hspg2 Meox1 Rgcc Emcn Pdgfb Arrdc4
−Log10 P
Log2 fold change
Old.P.value
BioCarta_2016
GO_Biological_Process_2021
KEGG_2019_Mouse
Reactome_2022
D. Col14+ Fibroblast
NS Log2 FC p-value p – value and log2 FC
Pltp Inmt St8sia2 Ptch1 Timp3 Gpc3 Rbp4 Nr2f2 Egflam
C3 Enpp2 Pdlim1
−Log10 P
Log2 fold change
Old.P.value
GO_Biological_Process_2021
KEGG_2019_Mouse
Reactome_2022
WikiPathways_2019_Mouse
E. Myofibroblast
NS Log2 FC p-value p – value and log2 FC
Hhip Igfbp5 Timp3 Adamts1 S100a10 Gsn
Igfbp7 B2m Dclk1 C1s1 Lbh AW112010 Ifitm1 Tnfaip3 C1ra Sod3 Enpp2 Ecscr Cp Itih5 Raph1 Tmem176b H2-Q4 Ch25h Midell2 Postn Mmp3 Nrp2
−Log10 P
Log2 fold change
Old.P.value
BioCarta_2016
GO_Biological_Process_2021
KEGG_2019_Mouse
Panther_2016
Reactome_2022
WikiPathways_2019_Mouse
F. Pericyte
NS Log2 FC p-value p – value and log2 FC
Nfkbie Col6a4 Cxcl10 Ctsc Susd2 AW112010 Gm13889 Nox4 Ifi27l2a Casp12 Cd82
−Log10 P
Log2 fold change
Old.P.value
GO_Biological_Process_2021
KEGG_2019_Mouse
Reactome_2022
WikiPathways_2019_Mouse

Supplemental Figure 7. Differential gene expression in additional endothelial and mesenchymal cell populations. Volcano plots (left) demonstrate genes which are most differentially over and under expressed in TNF vs WT mice in (A) gCAP1 cells, (B) aCAP cells, (C) proliferating EC cells, (D) collagen 14+ fibroblasts, (E) Myofibroblasts, and (F) Pericytes. Differentially regulated pathways in TNF vs WT cells are demonstrated by Cleveland dot plots (right) with colors indicating the database where pathway annotation was derived and x-axis indicating significance (p-value) of over-representation of the pathway in each cell population

A.

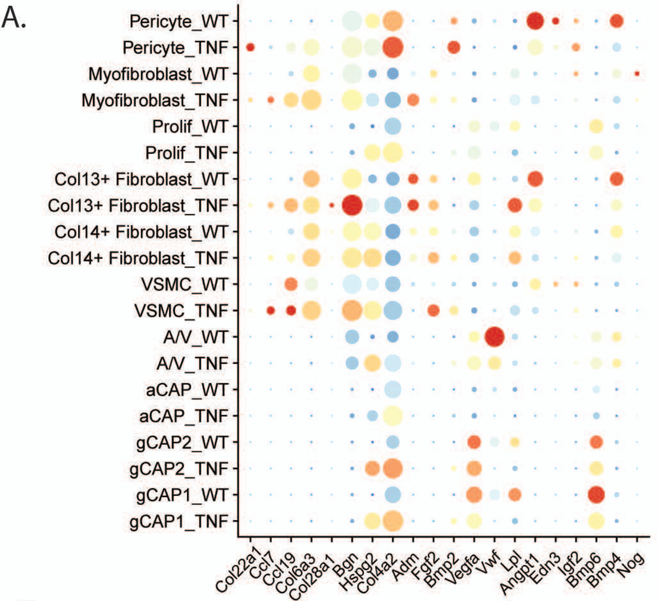

B.

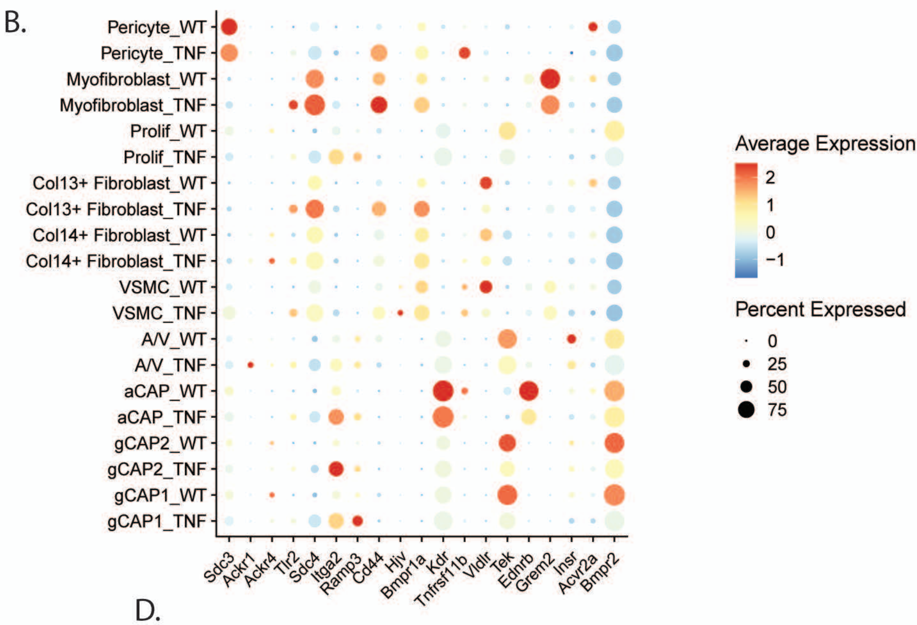

C.

Ligand	Receptor	Sender	Receiver	Score
Nog	Bmpr2	Myofibroblast	gCAP2	0.974955
Nog	Bmpr2	Myofibroblast	gCAP1	0.9717
Bmp4	Acvr2a	Col13+ Fibroblast	Col13+ Fibroblast	0.963599
Bmp6	Bmpr2	gCAP1	gCAP2	0.963371
Bmp6	Bmpr2	gCAP1	gCAP1	0.96232
Bmp4	Bmpr2	Col13+ Fibroblast	gCAP2	0.961405
Igf2	Insr	Myofibroblast	A/V	0.959702
Bmp4	Bmpr2	Col13+ Fibroblast	gCAP1	0.95815
Bmp4	Grem2	Col13+ Fibroblast	Myofibroblast	0.957198
Bmp6	Bmpr2	gCAP2	gCAP2	0.955055
Bmp6	Bmpr2	gCAP2	gCAP1	0.954004
Edn3	Ednrb	VSMC	aCAP	0.953497
Bmp4	Acvr2a	Col13+ Fibroblast	Myofibroblast	0.948611
Angpt1	Tek	Col13+ Fibroblast	A/V	0.948205
Lpl	Vldlr	gCAP1	VSMC	0.946577
Angpt1	Tek	Col13+ Fibroblast	gCAP2	0.943602
Lpl	Vldlr	gCAP2	VSMC	0.941858
Vwf	Tnfrsf11b	A/V	aCAP	0.941408
Bmp4	Acvr2a	Col14+ Fibroblast	Col13+ Fibroblast	0.941298
Vegfa	Kdr	gCAP1	aCAP	0.941177
Vegfa	Nrp1	gCAP1	aCAP	0.939263
Bmp4	Bmpr2	Col14+ Fibroblast	gCAP2	0.939104
Nog	Bmpr2	Myofibroblast	A/V	0.936615
Angpt1	Tek	VSMC	A/V	0.93634
Bmp4	Acvr1	Col13+ Fibroblast	Myofibroblast	0.936154
Nog	Bmpr2	Myofibroblast	aCAP	0.935992
Bmp4	Bmpr2	Col14+ Fibroblast	gCAP1	0.935849
Gpc3	Flt1	Col14+ Fibroblast	gCAP2	0.935479
Fgf2	Fgfr4	Myofibroblast	Col13+ Fibroblast	0.935387
Fgf1	Fgfr4	aCAP	Col13+ Fibroblast	0.935123
Bmp4	Grem2	Col14+ Fibroblast	Myofibroblast	0.934896
Bmp7	Bmpr2	Col14+ Fibroblast	gCAP2	0.93467
Vegfa	Kdr	Col13+ Fibroblast	aCAP	0.934104
Fgf7	Nrp1	Myofibroblast	aCAP	0.93291
Vegfa	Nrp1	Col13+ Fibroblast	aCAP	0.93219
Angpt1	Tek	VSMC	gCAP2	0.931737
Bmp6	Acvr2a	gCAP1	Col13+ Fibroblast	0.931162
Bmp7	Bmpr2	Col14+ Fibroblast	gCAP1	0.930387
Vegfa	Gpc1	gCAP1	Col13+ Fibroblast	0.930347
Efna1	Epha1	A/V	Col13+ Fibroblast	0.929029

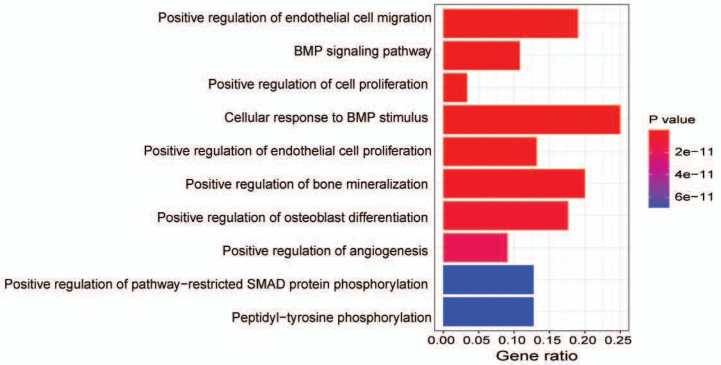

D.

Ligand	Receptor	Sender	Receiver	Score
Bmp2	Bmpr1a	gCAP2	Col13+ Fibroblast	0.975143
Bmp2	Hjv	gCAP2	VSMC	0.973179
Bmp2	Bmpr1a	gCAP1	Col13+ Fibroblast	0.972076
Bmp2	Hjv	gCAP1	VSMC	0.970113
Fgf2	Cd44	VSMC	Col13+ Fibroblast	0.968956
Adm	Ramp3	Myofibroblast	gCAP1	0.967932
Col4a2	Itga2	gCAP2	gCAP2	0.958641
Bmp2	Bmpr1a	A/V	Col13+ Fibroblast	0.957349
Fgf2	Cd44	Col14+ Fibroblast	Col13+ Fibroblast	0.956086
Bmp2	Bmpr1a	Prolif	Col13+ Fibroblast	0.955828
Bmp2	Hjv	A/V	VSMC	0.955385
Bmp2	Hjv	Prolif	VSMC	0.953864
Bmp2	Bmpr1a	VSMC	Col13+ Fibroblast	0.95358
Adm	Ramp3	Myofibroblast	aCAP	0.953385
Fgf2	Sdc4	VSMC	Col13+ Fibroblast	0.952542
Hspg2	Itga2	gCAP2	gCAP2	0.951834
Bmp2	Hjv	VSMC	VSMC	0.951617
Bgn	Tlr2	Col13+ Fibroblast	Myofibroblast	0.950039
Col4a2	Itga2	gCAP1	gCAP2	0.949881
Col28a1	Itga2	Col13+ Fibroblast	gCAP2	0.949281
Bmp2	Bmpr1a	Col14+ Fibroblast	Col13+ Fibroblast	0.946327
Bgn	Tlr2	VSMC	Myofibroblast	0.94497
Bmp2	Hjv	Col14+ Fibroblast	VSMC	0.944364
Col6a3	Itga2	VSMC	gCAP2	0.943428
Ccl19	Ackr4	Myofibroblast	Col14+ Fibroblast	0.94303
Ccl19	Ackr4	Col13+ Fibroblast	Col14+ Fibroblast	0.941953
Ccl7	Ackr1	VSMC	A/V	0.941076
Fgf2	Sdc3	VSMC	VSMC	0.940399
Col22a1	Itga2	Myofibroblast	gCAP2	0.940002
Fgf2	Sdc4	Col14+ Fibroblast	Col13+ Fibroblast	0.939672
Hspg2	Itga2	gCAP1	gCAP2	0.93967
Tgfb1	Itgb8	aCAP	Col13+ Fibroblast	0.939069
Hspg2	Itga2	Prolif	gCAP2	0.93877
Hspg2	Itga2	gCAP2	Prolif	0.936813
Sema3b	Nrp2	aCAP	gCAP2	0.936532
Col5a2	Itga2	Col13+ Fibroblast	gCAP2	0.935513
Ccl7	Ackr1	Myofibroblast	A/V	0.935292
Hspg2	Itga2	A/V	gCAP2	0.935096
Bgn	Tlr2	Col13+ Fibroblast	Col13+ Fibroblast	0.934898
Adm	Ramp3	Myofibroblast	gCAP2	0.934682

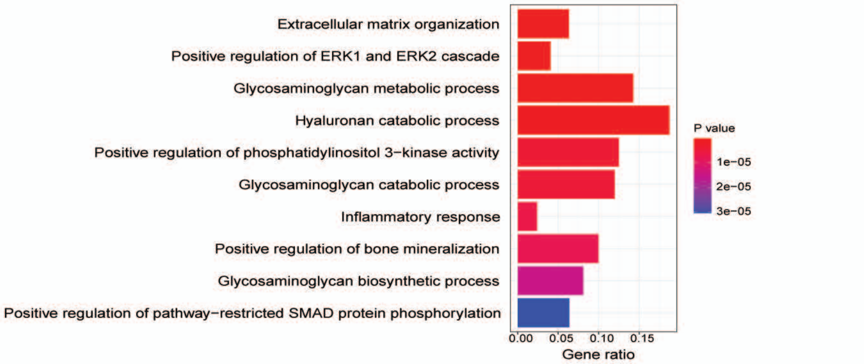

Supplemental Figure 8. Ligand-receptor analysis demonstrates prominent role of loss of BMPR2 signaling in TNF mediated PAH. Differentially regulated ligands (A) and receptors (B) were identified across cell types by mulitnichenetr and are represented as dotplots. The top ligand-receptor pairs between two interacting cell types were scored according to probability and ranked in terms of differential regulation in TNF vs WT conditions with those down-regulated in TNF (or up in WT) shown in (C) and those up-regulated in TNF conditions shown in (D). Pathways indicated by upregulated L:R pairs are shown below

A. gCAP1

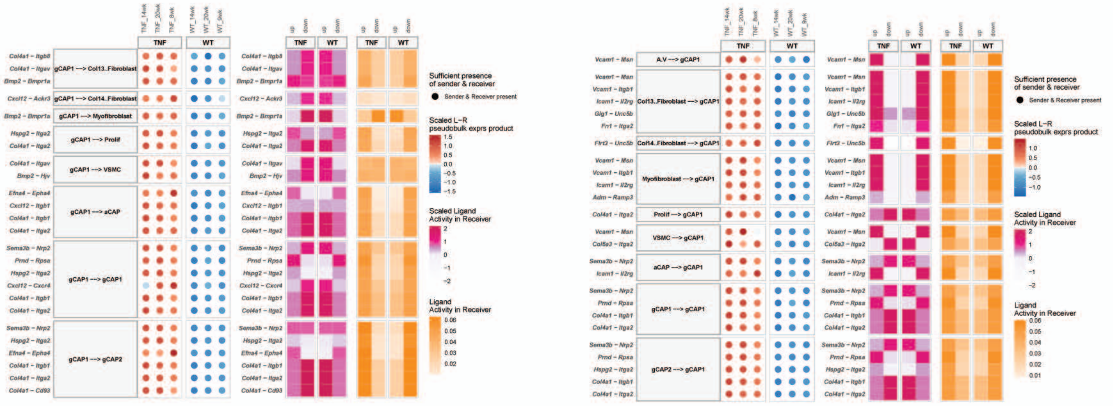

B. A/V

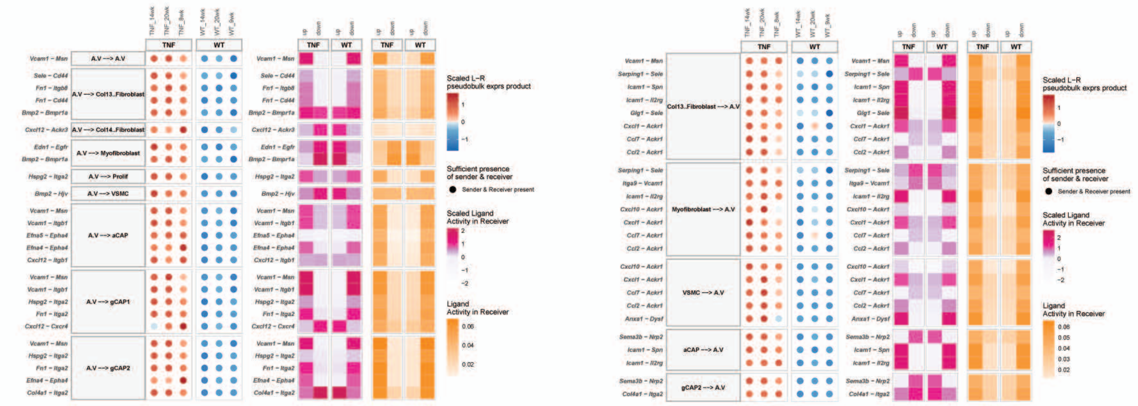

C. VSMC

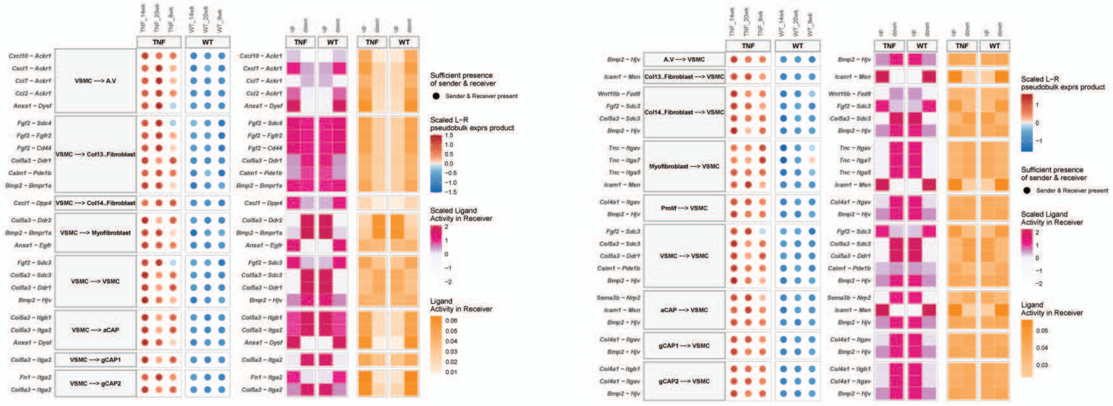

D. Collagen 13+ fibroblasts

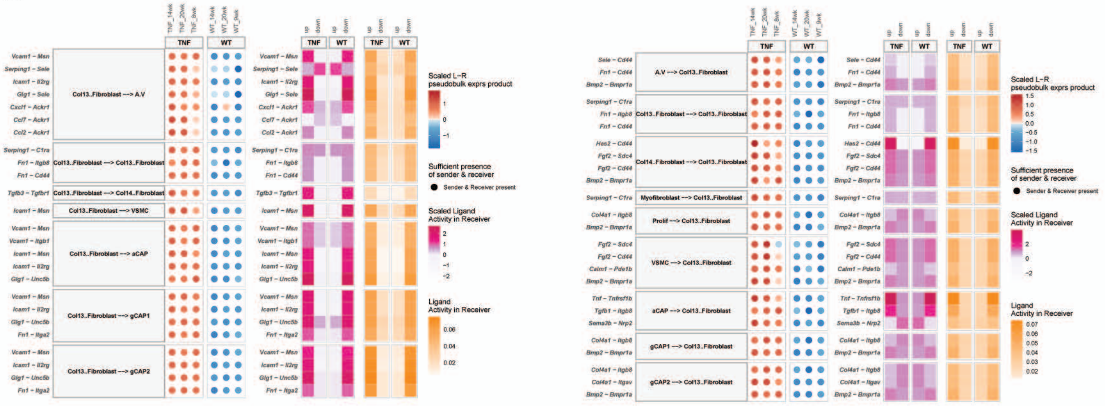

Supplemental Figure 9. Ligand receptor interactions in key cell types in TNF-Tg PAH as senders and receivers. Multinichenetr was used to visualize per sample the scaled product of ligand and receptor expression in (A) gCAP1 cells (B) A/V endothelial cells (C) VSMCs and (D) Col13+ Fibroblasts. Each cell types' interactions are represented both as senders (producers of ligands, left) as receivers (producers of receptors, right). Dot-plots (left) represent the predicted ligand-receptor interaction per genotype/condition in each pair of indicated cell types using pseudo-bulk expression. Heatmaps (right) indicating the scaled (left) and total (right) ligand activity are expressed for each ligand-receptor pair across WT and TNF-Tg conditions with colors used to indicate ligand activity.

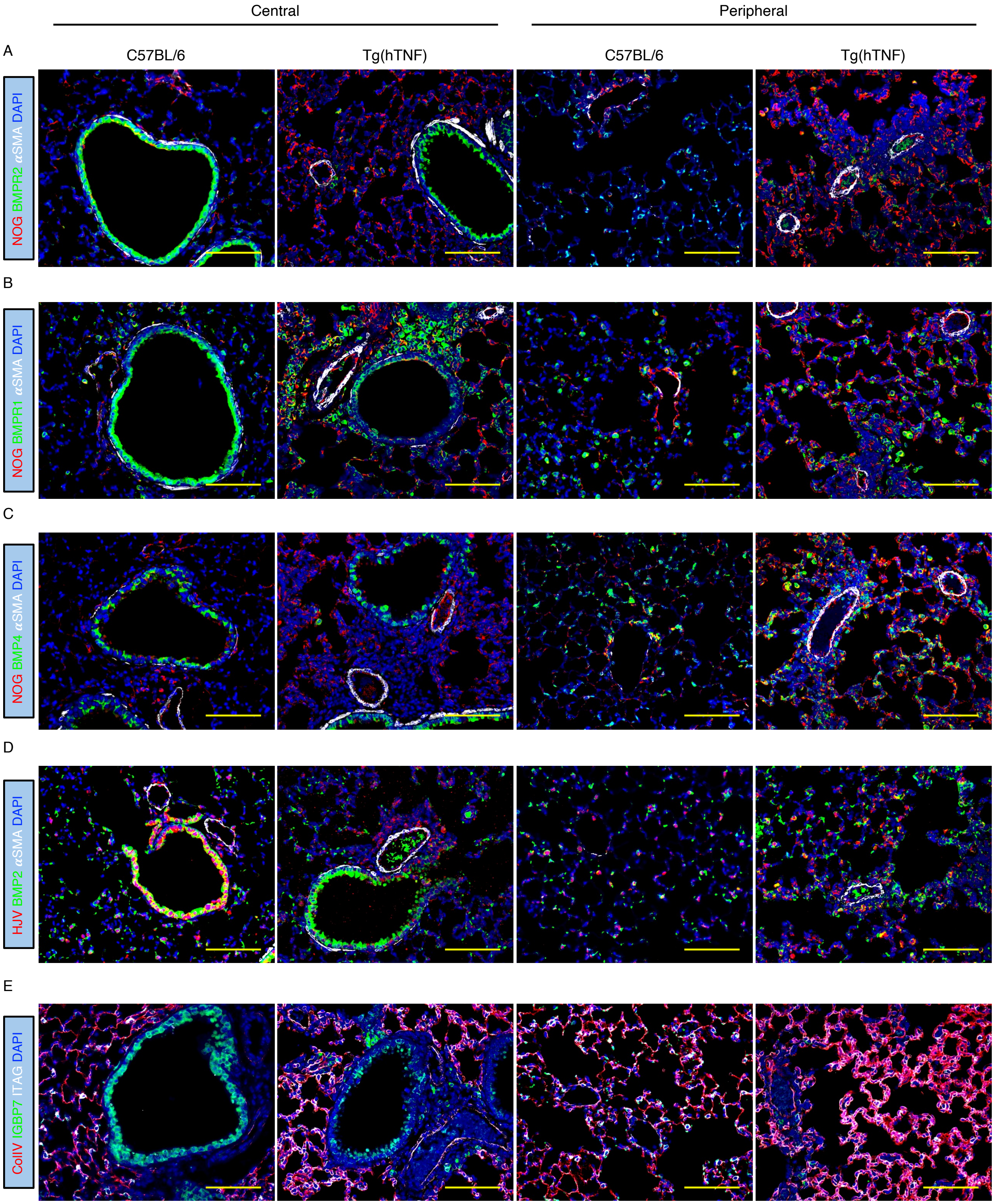
Central
Peripheral
A
C57BL/6
Tg(hTNF)
C57BL/6
Tg(hTNF)
NOG BMPR2 αSMA DAPI
B
NOG BMPR1 αSMA DAPI
C
NOG BMP4 αSMA DAPI
D
HJV BMP2 αSMA DAPI
E
ColIV IGBP7 ITAG DAPI

Supplemental Figure10. Immunofluorescent co-staining of molecules from BMP and TGF-beta pathways in 8 week old WT and TNF-Tg mice. Lungs from 8 week old mice (n=3 per condition) were collected and paraffin embedded and subsequently immunostaining was performed to colocalize relevant molecules and structures. BMP pathways were assessed in (A) with Bmpr2/Nog/aSMA, (B) with Bmpr1a/Nog/aSMA, (C) Bmp4/Nog/aSMA, and (D) Bmp2/Hjv/aSMA; TGF-beta/collagen pathway is indicated by (E) Igfbp7/Col4a1/Itga2 staining. In each instance colors are indicated in the inset titles above the images. Top rows for each stain indicate WT mice and bottom rows TNF-Tg mice. Images were taken at 20x magnification and represent central bronchiolar regions (left) and the lung periphery (right). Representative images

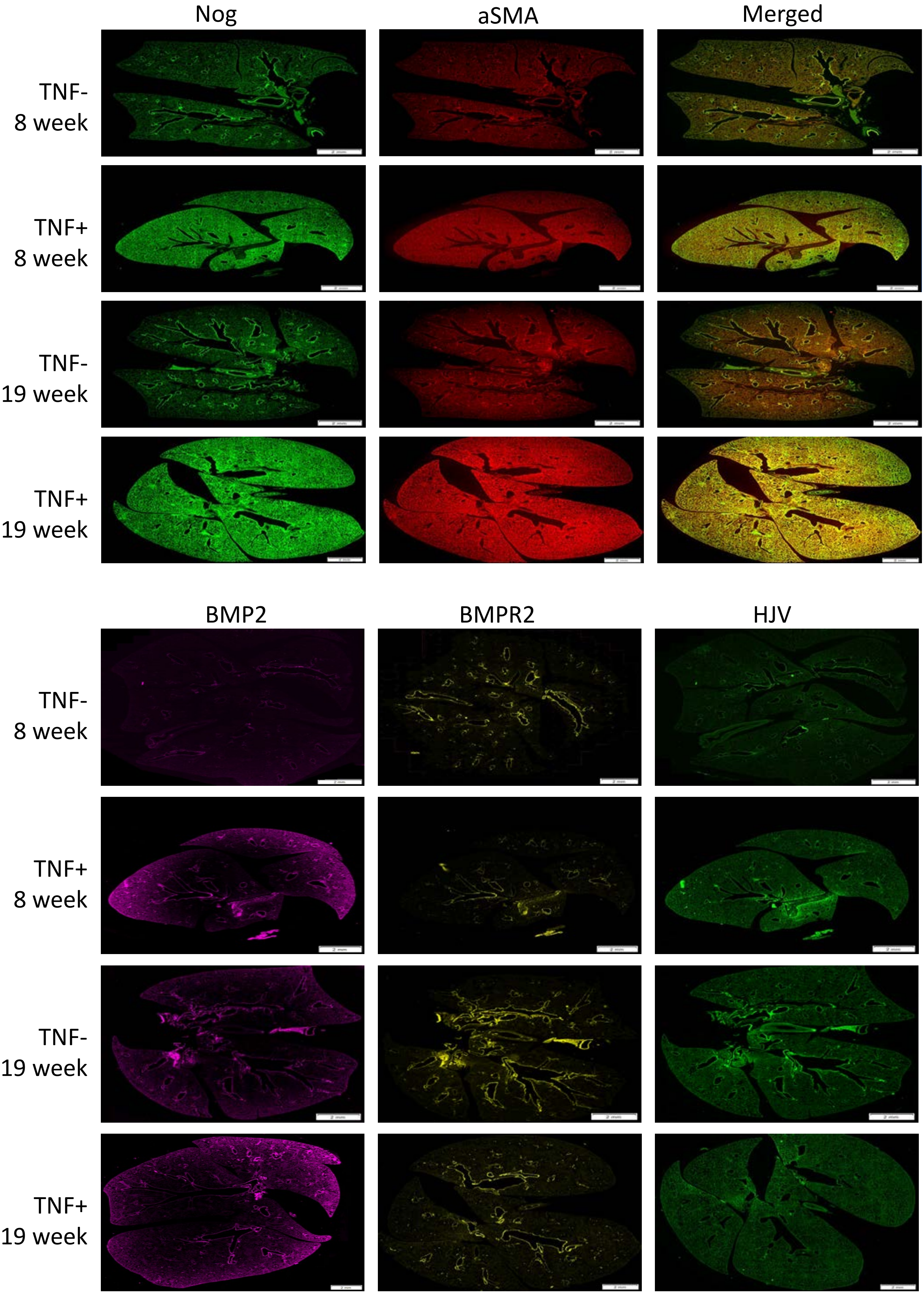
Nog
aSMA
Merged
TNF-
8 week
TNF+
8 week
TNF-
19 week
TNF+
19 week
BMP2
BMPR2
HJV
TNF-
8 week
TNF+
8 week
TNF-
19 week
TNF+
19 week

Supplemental Figure 11. Low magnification immunofluorescent co-staining of BMP molecules. Lungs from 8 and 20 week old mice (n=5 per condition / timepoint) were collected and paraffin embedded and subsequently immunostaining was performed to colocalize relevant molecules at 10x. Co-immunostaining is shown (A) Nog/aSMA and merged images, and (B) Hjv, Bmp2, and Bmpr2. Representative images.

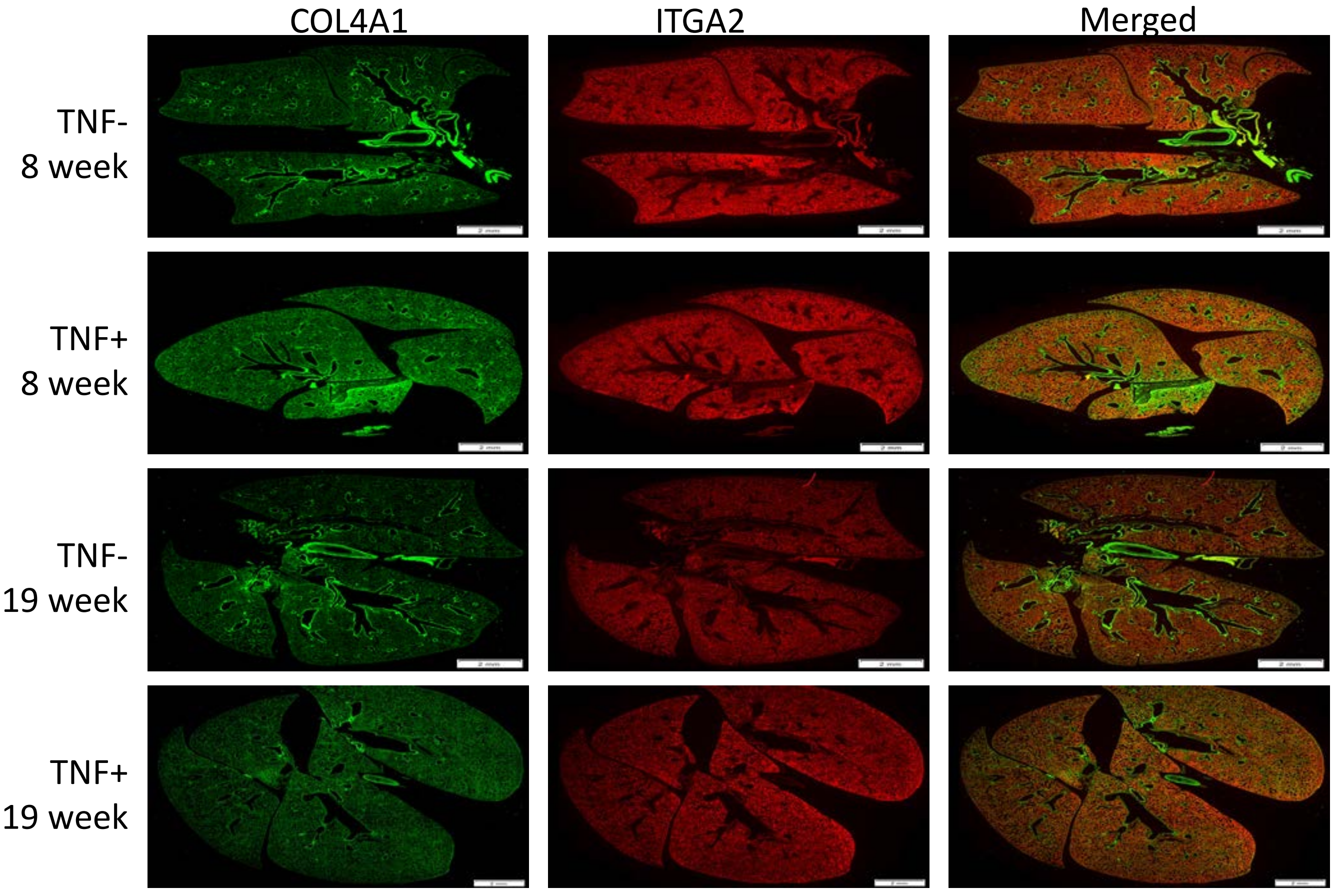
COL4A1
ITGA2
Merged
TNF-
8 week
TNF+
8 week
TNF-
19 week
TNF+
19 week

Supplemental Figure 12. Low magnification immunofluorescent co-staining of Col4a1/Itga2. Lungs from 8 and 20 week old mice (n=3-5 per condition / timepoint) were collected and paraffin embedded and subsequently immunostaining was performed to colocalize relevant molecules at 10x. Co-immunostaining is shown Col4a1/Itga2/Merge. Representative images.

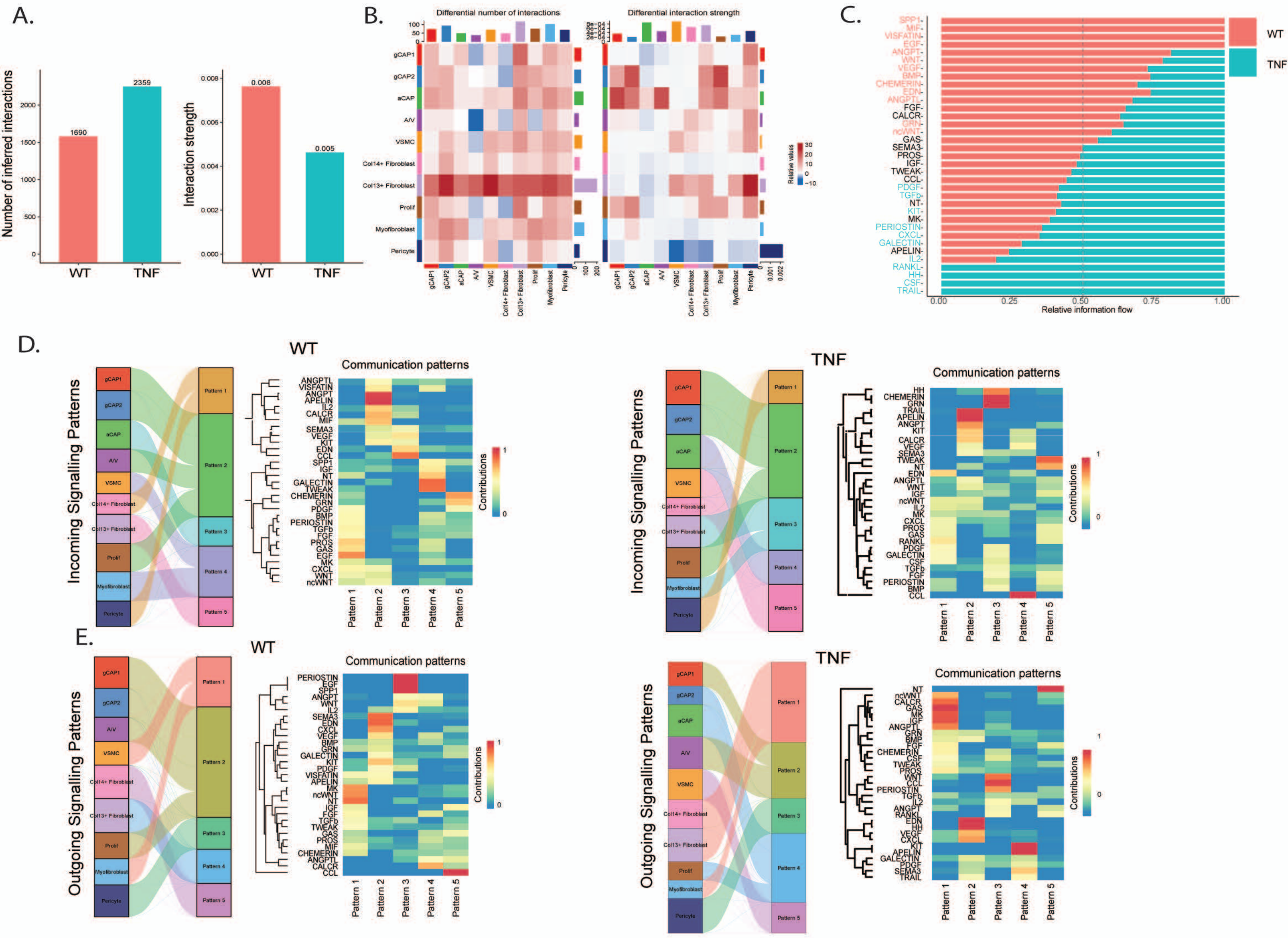
A.
Number of inferred interactions
1690
2359
WT
TNF
Interaction strength
0.008
0.005
B.
Differential number of interactions
Differential interaction strength
gCAP1
gCAP2
aCAP
A/V
VSMC
Col14+ Fibroblast
Col13+ Fibroblast
Prolif
Myofibroblast
Pericyte
Relative values
C.
WT
TNF
SPP1
MIF
VISFATIN
EGF
ANGPT
WNT
VEGF
BMP
CHEMERIN
EDN
ANGPTL
FGF
CALCR
GRN
ncWNT
GAS
SEMA3
PROS
IGF
TWEAK
CCL
PDGF
TGFb
NT
KIT
MK
PERIOSTIN
CXCL
GALECTIN
APELIN
IL2
RANKL
HH
CSF
TRAIL
Relative information flow
D.
WT
TNF
Incoming Signalling Patterns
Communication patterns
Contributions
Pattern 1
Pattern 2
Pattern 3
Pattern 4
Pattern 5
E.
WT
TNF
Outgoing Signalling Patterns
Communication patterns
Contributions

Supplemental Figure 13. Network based analysis of perturbations in cell-cell communication in TNF mediated PH. Cell-cell communication was investigated using Cellchat to assess global changes in cell signaling across cell types. A. Differential number and strength of cell-cell interactions in endothelial and mesenchymal cells across conditions. B. Heatmap of differential number of interactions and interaction strength across WT and TNF conditions for each cell type. The top colored bar plot represents the sum of column of values displayed in the heatmap (incoming signaling). The right colored bar plot represents the sum of row of values (outgoing signaling). In the central heatmap, red (or blue) represents increased (or decreased) signaling in the TNF condition compared to WT. C. Stacked bar plot indicating conserved and context-specific signaling pathways across WT and TNF conditions. Significant signaling pathways are ranked based on differences in the overall information flow within the inferred networks between WT and TNF lungs. The top signaling pathways colored red are enriched in WT, and those colored green are enriched in the TNF. Incoming (D.) and outgoing (E.) signaling patterns shared across cell types in WT (left) and TNF-Tg (right) conditions are visualized as both alluvial plots (left) and heatmaps (right) to indicated cell types and pathways which are differentially regulated by each cell signaling pattern.

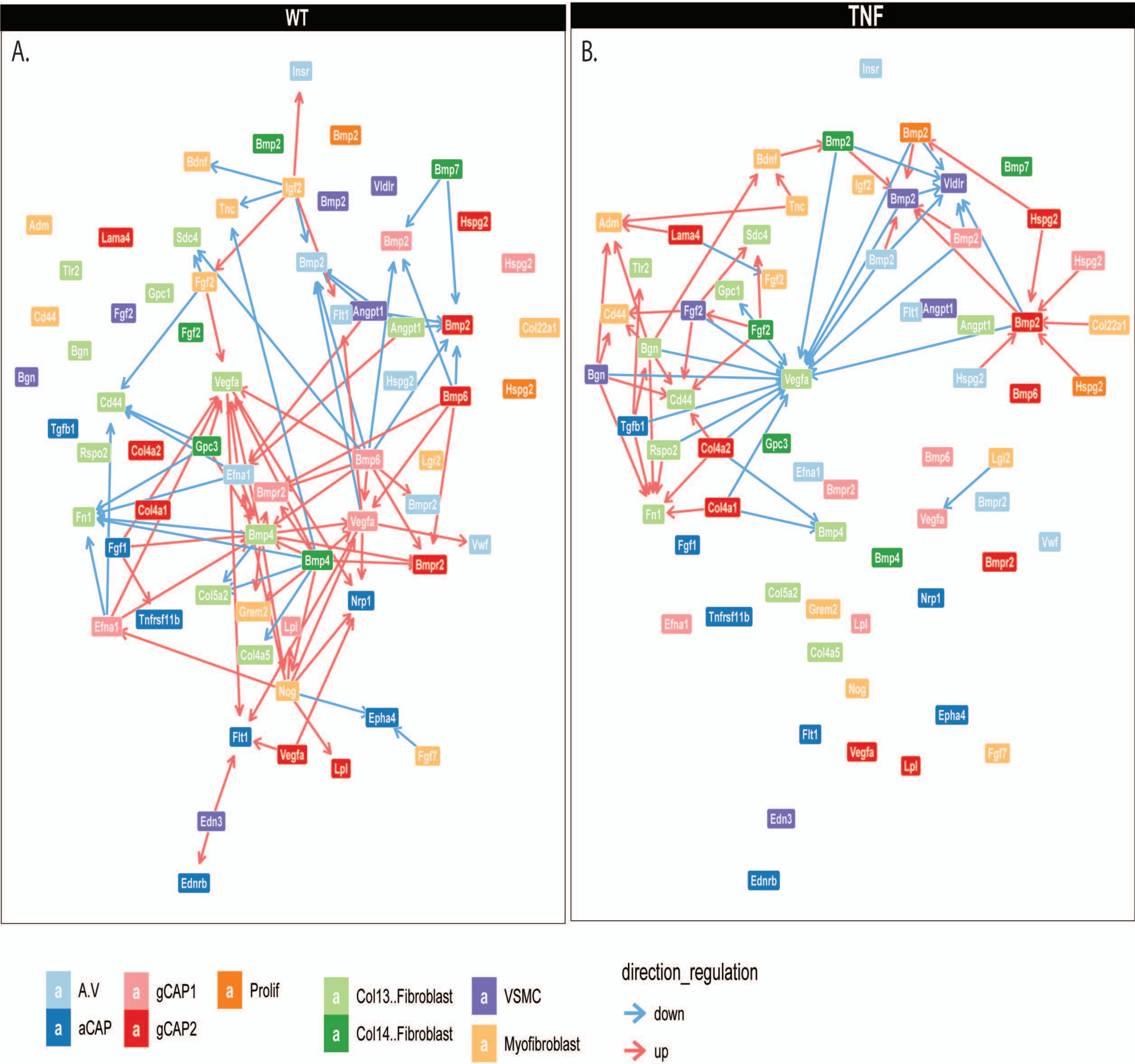
WT
TNF
A.
B.
A.V
aCAP
gCAP1
gCAP2
Prolif
Col13..Fibroblast
Col14..Fibroblast
VSMC
Myofibroblast
direction_regulation
down
up

Supplemental Figure 14. Intercellular regulatory network systems view of ligand-receptor interactions. A network analysis was generated using multinichenetR package to show the gene regulatory links between ligands from sender cell types to their induced ligands/receptors in receiver cell types in WT (left) and TNF (right) conditions. Lines show if the ligand/receptor in the receiver is a potential downstream target of the ligand based on literature/database knowledge and correlation in expression across samples.

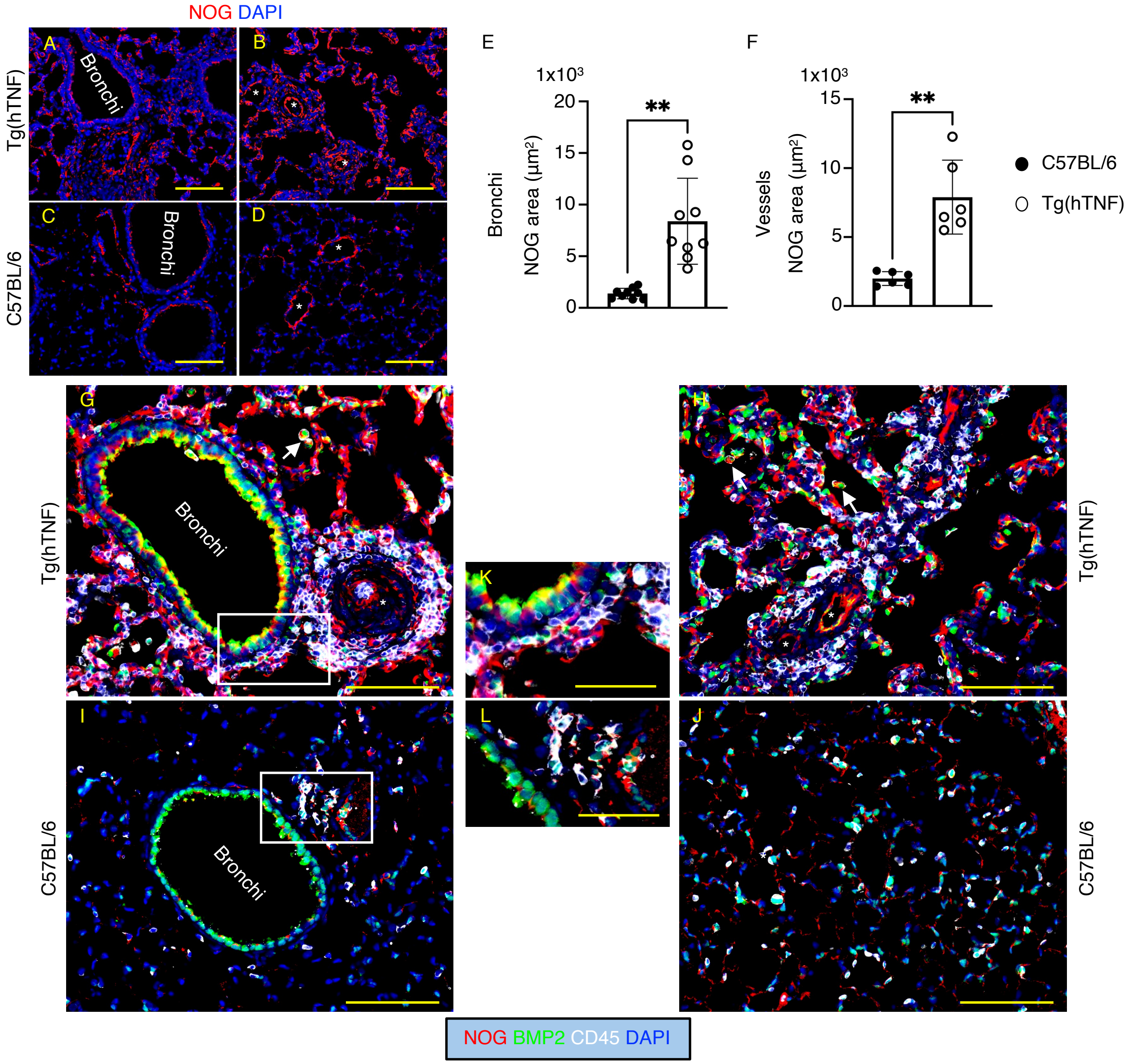

NOG DAPI
A
B
C
D
Bronchi
Bronchi
Tg(hTNF)
C57BL/6
E
F
1x10³
Bronchi
NOG area (μm²)
Vessels
NOG area (μm²)
**
**
0
5
10
15
20
C57BL/6
Tg(hTNF)
G
H
I
J
K
L
Bronchi
Bronchi
Tg(hTNF)
C57BL/6
NOG BMP2 CD45 DAPI

Individual channels, A-D

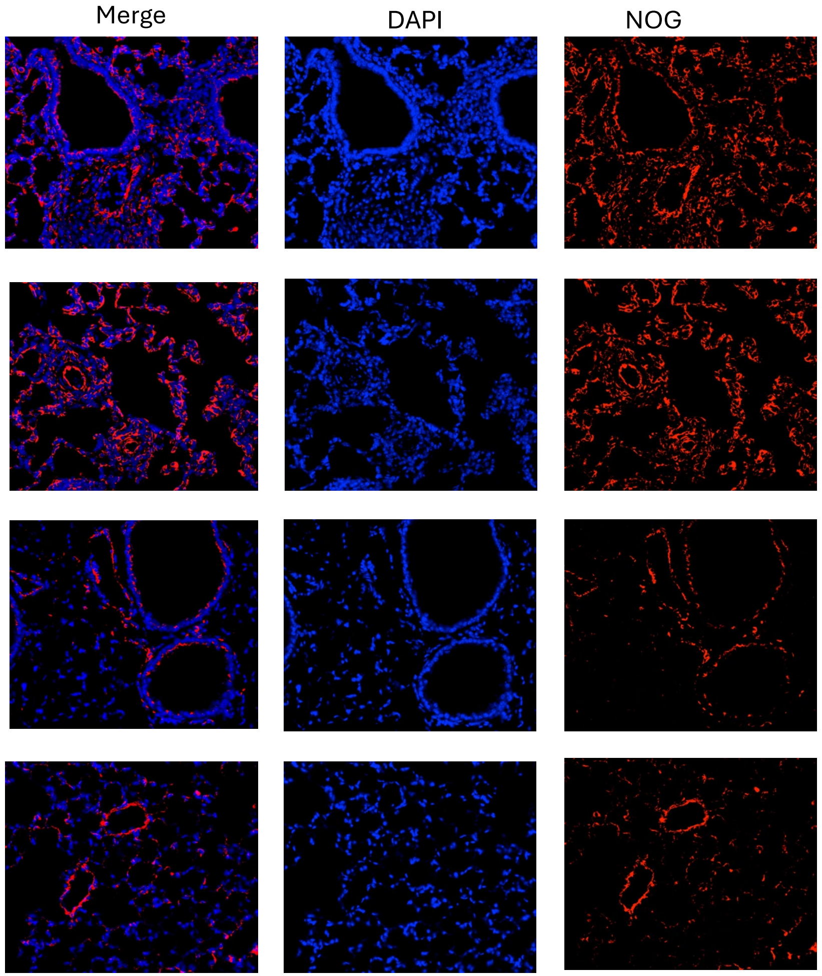

Individual channels, G-J

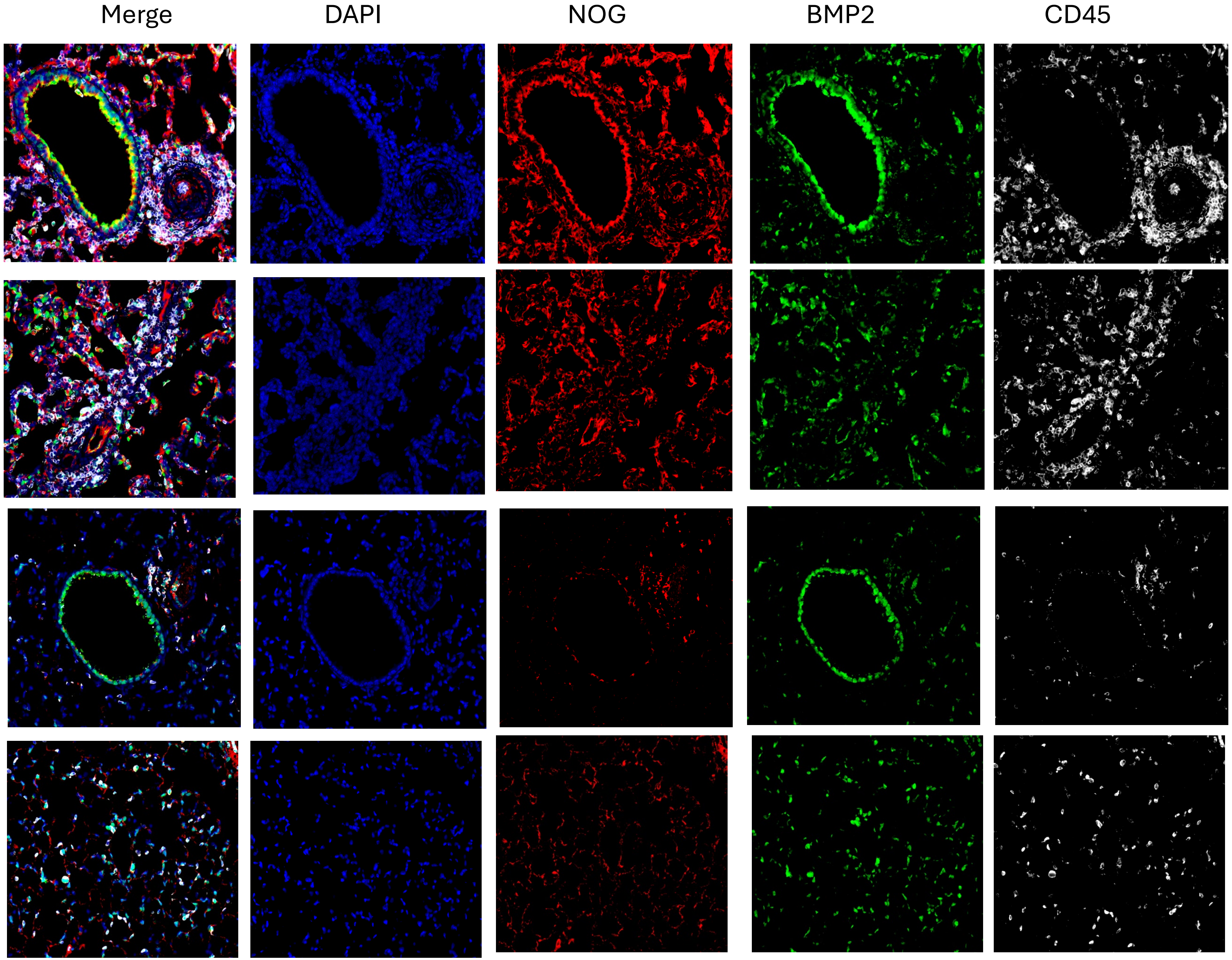

Supplementary Fig 15. NOG is mainly expressed by CD45^{-} stromal cells and increased in vascularized and bronchial areas in lungs of Tg(hTNF) mice. Lungs from Tg(hTNF) and C57BL/6 mice were stained with antibodies specific for NOG and nuclei labeled with DAPI. NOG expression is increased in A) bronchial and B) vascularized areas in lungs from TNF Tg mice compared to NOG expression in C) bronchial and D) vascularized areas in lungs of C57BL/6 mice. Asterisks depict lumen of blood vessels. Scale bars = 100 µm. 3 random 200x pictures were taken in bronchial and vascularized areas in the lungs of Tg(hTNF) (n = 2-3, 6-9 pictures) and C57BL/6 mice (n = 2-3, 6-9 pictures). Representative 200x pictures are shown. Area of NOG positive signal in 200x random fields was measured with NIH image J using identical settings. Area covered by NOG signal in E) bronchial and F) vascularized regions in the lungs of Tg(hTNF) mice is significantly bigger than areas of NOG signal in C57BL/6 mice. Graphs represent the mean ± SEM. Statistical significance was calculated with paired two tailed Student's t test. **, $p \leq 0.005$. Lung sections from G, H) TNF Tg and I, J) C57BL/6 mice were stained with antibodies against NOG, BMP2 and CD45. Nuclei were labeled with DAPI. NOG is expressed mainly by CD45^{-} stromal cells. White arrows point to a few CD45^{+}NOG^{+} cells with macrophage like morphology in the alveolar spaces. Asterisks depict lumen of vessels and scale bars represent 100 µm. Insets of K) Tg(hTNF) and L) C57BL/6 mice show the lack of colocalization between CD45 and NOG in peribronchial areas. Scale bars: 50 µm. A second panel shows the individual channels for each image A-D and G-J

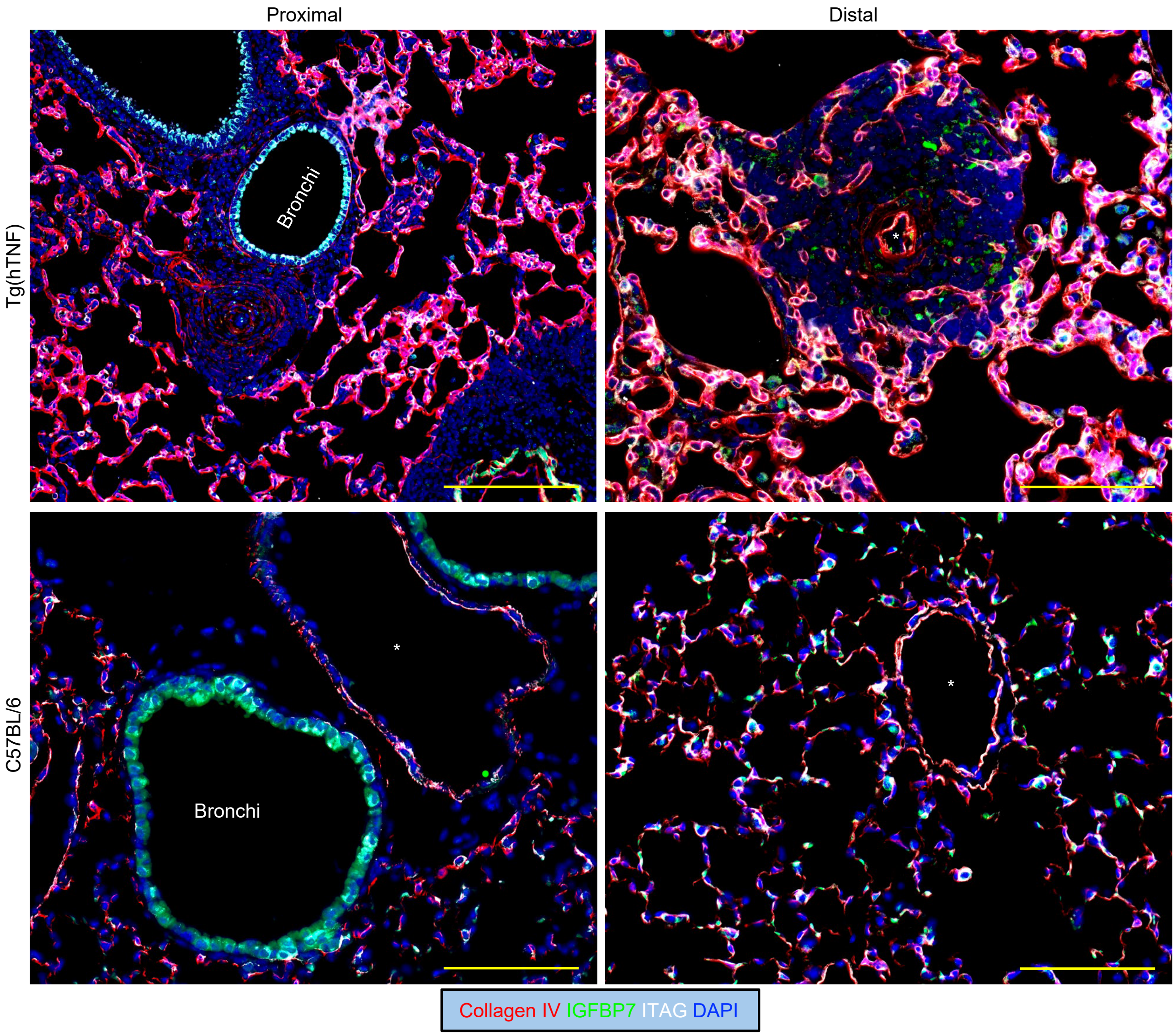

Proximal
Distal
Tg(hTNF)
C57BL/6
Bronchi
Bronchi
*
*
*
Collagen IV IGFBP7 ITAG DAPI

Supplementary Fig 16. Hi resolution image of collagen IV staining. Immunofluorescent co-staining of molecules Col4a1/Itga2/IGFBP7 staining. Colors are indicated in the inset titles above the images. Top rows for each stain indicate TNF mice and bottom rows WT mice. Images were taken at 20x magnification and represent central bronchiolar regions (left) and the lung periphery (right). Representative images.

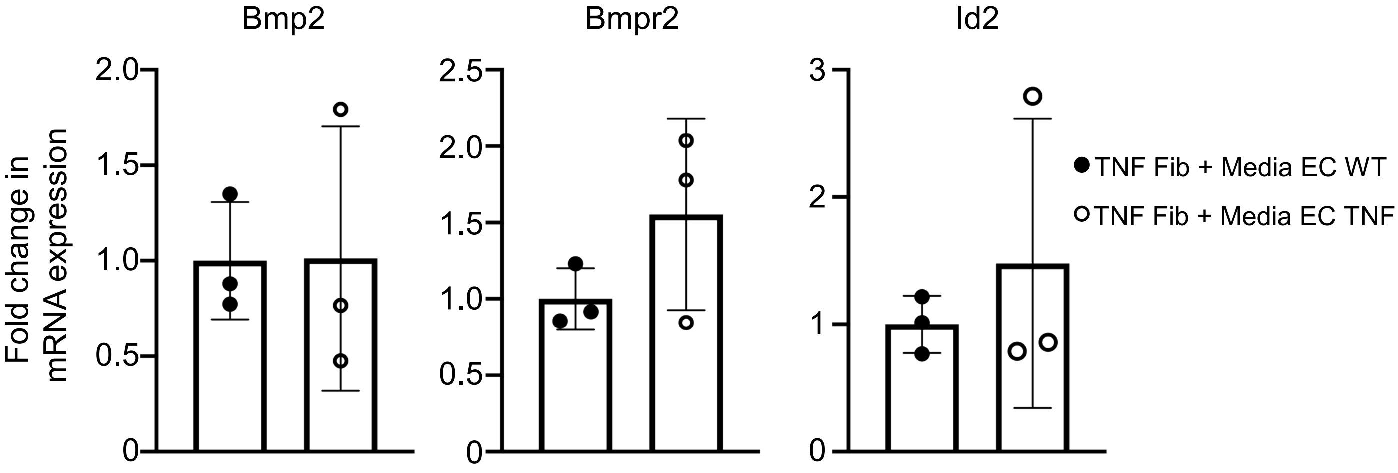
Bmp2
Bmpr2
Id2
Fold change in mRNA expression
2.0
1.5
1.0
0.5
0
2.5
2.0
1.5
1.0
0.5
0
3
2
1
0
TNF Fib + Media EC WT
TNF Fib + Media EC TNF

Supplementary Fig 17. Media from endothelial cells of Tg(hTNF) does not alter BMP2-BMPR2 axis. Fibroblasts (FB) from Tg(hTNF) mice were cultured with media from endothelial cells (EC) from C57BL/6 (WT) or Tg(hTNF) mice activated with TNF for 72h. RNA was isolated from cocultures 6h later and cDNA prepared for qPCR. Gene expression was normalized to18s and gene expression in different cultures was compared to FB from Tg(hTNF) mice stimulated with media from WT EC. A) BMP2, B) BMPR2 and C) Id2 in TNF EC were not affedted by media from TNF EC.

	Lung number (pooled)	Single cell of total cells	Viable cells of single cells (7-AAD-)	CD45+ (leukocytes) of Viable cells	CD326+ (epithelial cells) of Viable cells	CD45-/CD326- (non-epithelial non-leukocytes) cells of Viable cells	CD31- (non-endothelial cells) of CD45-/CD326-	CD31+ (endothelial cells) of CD45-/CD326-
WT 8 week	3	79.7 (119513)	92.1 (110086)	26.3 (28928)	9.11 (10032)	67.1 (73852)	59.4 (43886)	40.7 (30058)
TNF 8 week	3	78.0 (116951)	83.6 (97739)	63.9 (62469)	11.4 (11145)	30.7 (30012)	75.4 (22764)	24.2 (7260)
WT 14 week	4	79.8 (119640)	93.5 (111855)	24.2 (27120)	9.16 (10245)	68.7 (76815)	64.0 (49155)	36.0 (27840)
TNF 14 week	4	78.6 (117835)	82.3 (96928)	79.0 (76530)	7.48 (7255)	17.2 (16645)	84.3 (14040)	15.7 (2610)
WT 20 week	4	89.3 (112419)	83.7 (88186)	66.2 (58417)	5.58 (4922)	29.3 (25826)	53.6 (13801)	46.4 (11870)
TNF 20 week	5	85.1 (121008)	81.8 (90456)	89.3 (80736)	4.74 (3836)	7.47 (6760)	87.6 (5923)	12.4 (840)

Supplemental table 1. **Summary of FACS results.** Staining of single cells from lungs was performed for CD45, CD326, and CD31 and a live/dead stain. Cell proportions are reflected as percentage of gated cells per 150,000 cells counted.

	WT 8wk	WT 14wk	WT 20wk	TNF 8wk	TNF 14wk	TNF 20wk	Total
**gCAP1**	2271	2383	2827	2993	2279	2040	14793
**gCAP2**	2121	1680	2087	1191	913	673	8665
**aCAP**	914	736	778	808	680	541	4457
**A/V**	466	488	578	464	267	529	2792
**VSMC**	126	82	134	217	450	356	1365
**Col14+ Fibroblast**	128	106	105	125	226	447	1137
**Col13+ Fibroblast**	482	257	203	53	57	58	1110
**Prolif**	45	14	44	439	284	164	990
**Myofibroblast**	229	154	230	79	56	78	826
**Epithelial**	63	134	65	20	447	45	774
**Mesothelial**	30	77	51	36	124	324	642
**Lymphoid**	15	10	6	5	14	538	588
**Lyz2+ Myeloid**	2	1	8	27	60	380	478
**PF4+ Myeloid**	46	21	25	68	77	122	359
**Pericyte**	66	57	60	23	2	0	208
**Total**	7004	6200	7201	6548	5936	6295	39184

Supplemental table 2. Number of cells per UMAP cluster across conditions. Total number of cells sequenced per condition is enumerated as a sum in the bottom row, cell types are listed in order of frequency across all conditions.

	gCAP1	gCAP2	A/V	aCAP	Prolif	VSMC	Col14+ F	Col13+ F	Myofib	Pericyte
FADD/TRADD apoptosis	**6.6E-03**	3.5E-01	**5.3E-04**	**1.8E-03**	**4.5E-02**	**2.0E-03**	**3.5E-02**	**1.1E-03**	**1.3E-03**	**7.3E-05**
RIP3K necroptosis	**2.8E-02**	**4.1E-02**	**2.7E-02**	1.0E-01	2.6E-01	6.7E-02	9.4E-01	**1.1E-02**	**2.0E-02**	3.9E-01
PI3 kinase	**1.8E-02**	**3.4E-02**	**1.2E-03**	**6.3E-05**	**1.6E-02**	**2.9E-02**	1.2E-01	8.1E-02	**1.3E-02**	5.2E-02
MAP3K	**4.8E-02**	9.5E-01	4.7E-01	2.3E-01	8.5E-01	3.9E-01	1.8E-01	1.2E-01	**2.4E-02**	9.4E-01
JAK-STAT	8.3E-01	1.4E-01	**4.5E-02**	**3.7E-04**	4.4E-01	6.1E-02	7.8E-01	5.5E-02	**2.9E-02**	7.0E-01
NFkB	9.8E-01	**2.9E-02**	6.2E-01	**4.0E-04**	1.4E-01	**3.6E-02**	4.5E-01	3.2E-01	**5.0E-02**	1.7E-01
p38-ERK	**6.1E-04**	6.8E-02	**1.8E-02**	2.3E-01	**4.9E-02**	**2.5E-02**	**1.1E-02**	**2.9E-03**	5.4E-01	1.3E-01

Supplemental table 3. Cell-specific analysis of KEGG pathways downstream of TNF. Annotations are listed in scientific notation and indicate p-values for the likelihood of pathway over-representation in the particular cell population, $p<0.05$ is bolded.
