# Supplementary figures and images for: TNF drives aberrant BMP signaling to induce endothelial and mesenchymal dysregulation in pulmonary hypertension
Source: JCI Insight. 2025 Jun 26;10(14):e174456. doi: 10.1172/jci.insight.174456 (PMC12288976; doi:10.1172/jci.insight.174456)

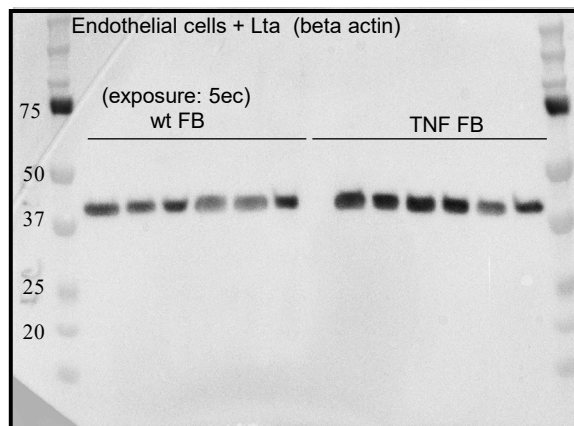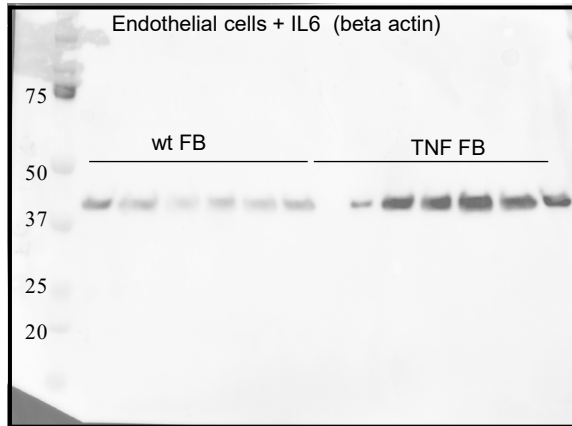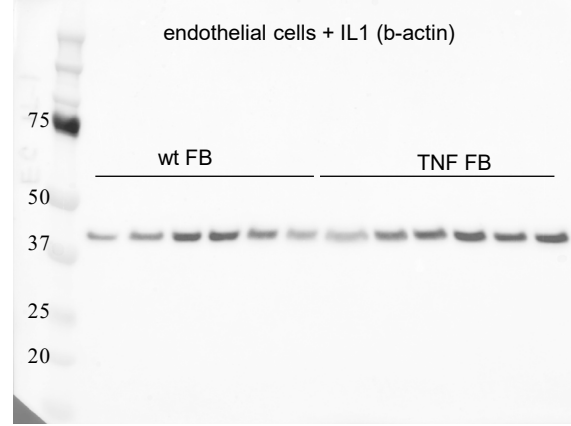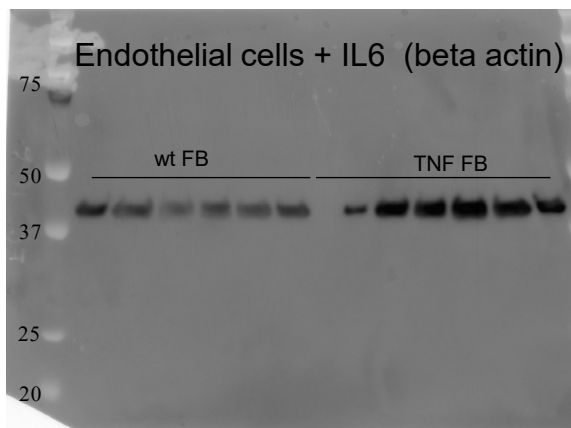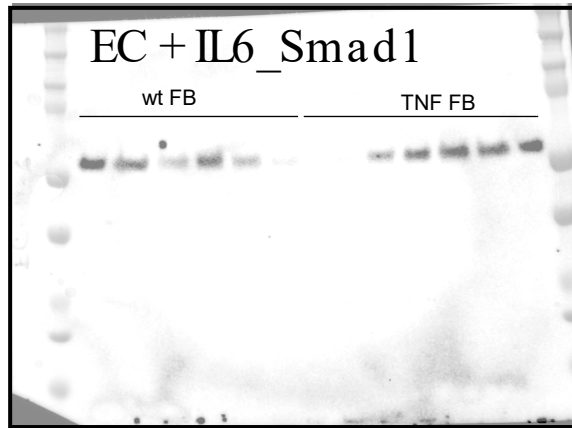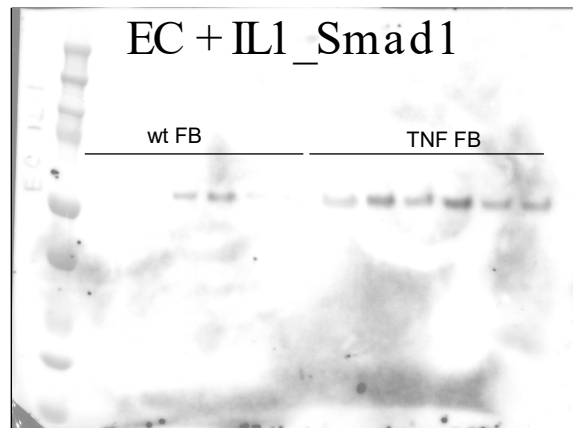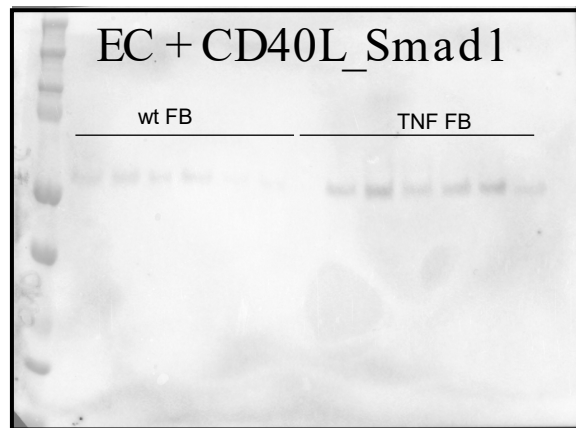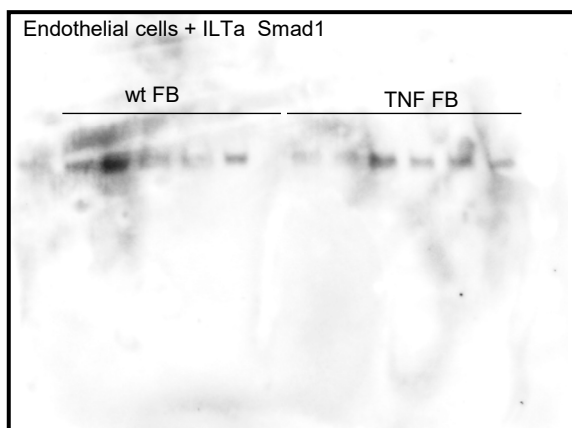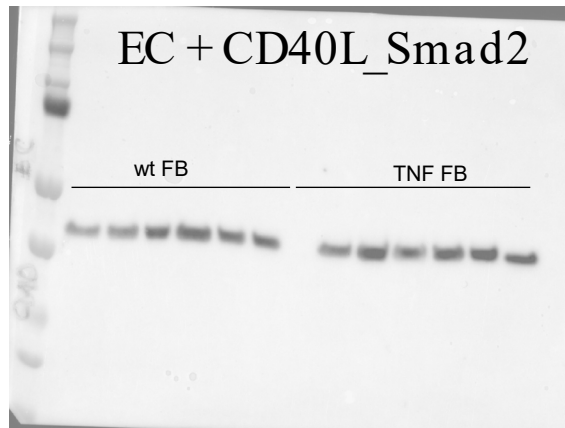

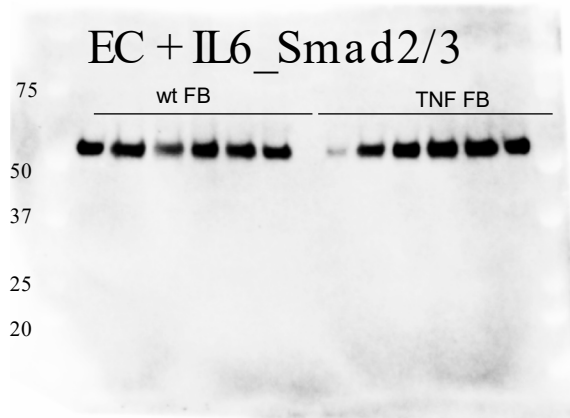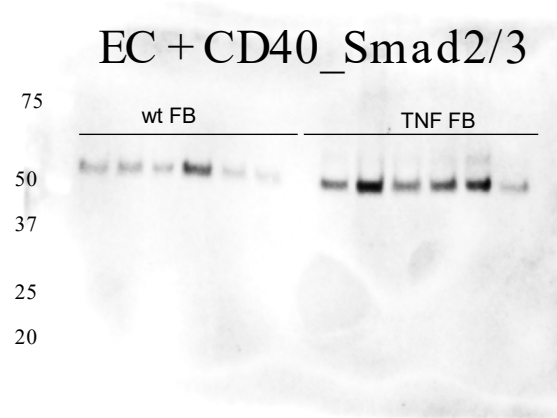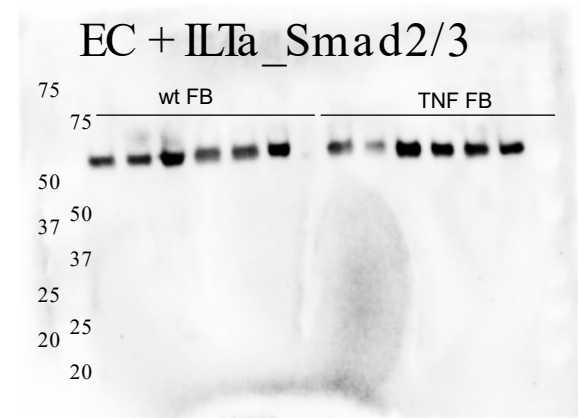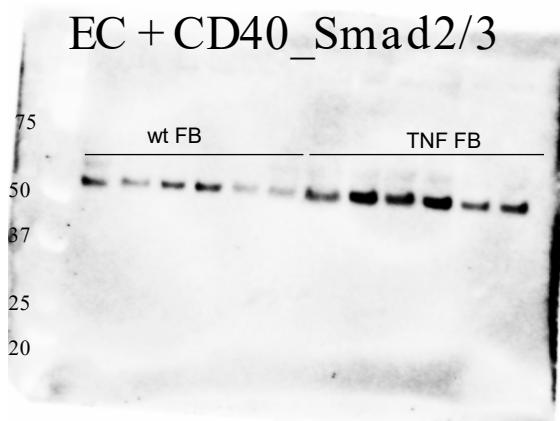

Smad2/3 = 60 Kda  
Smad1 = 60 KDa

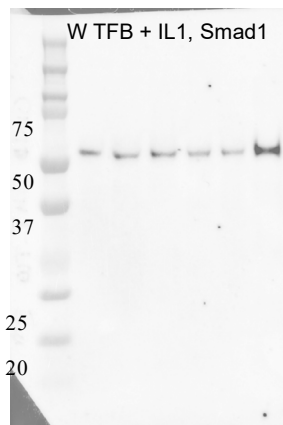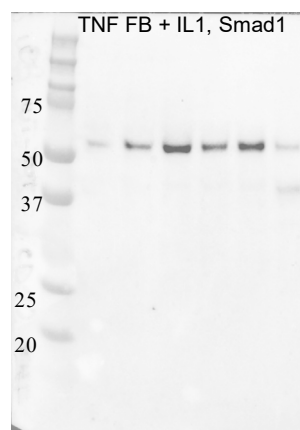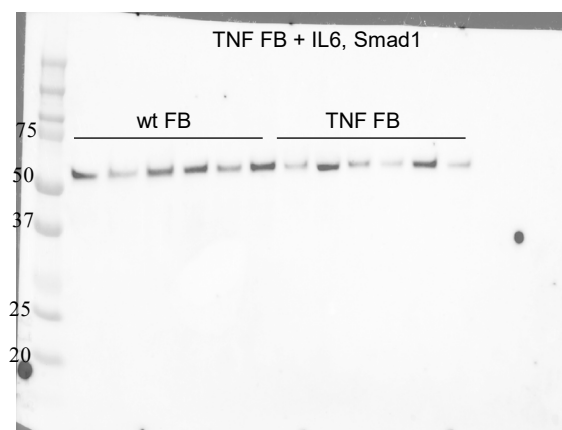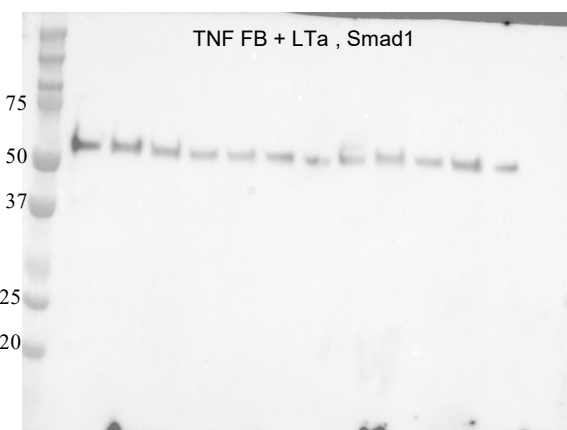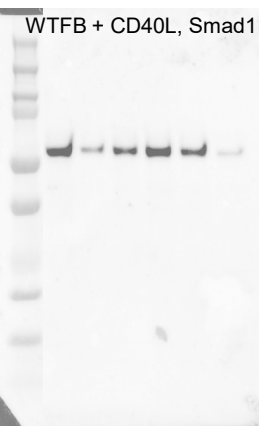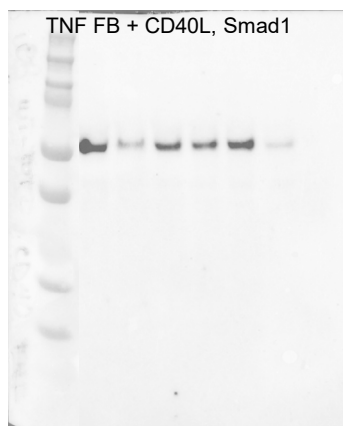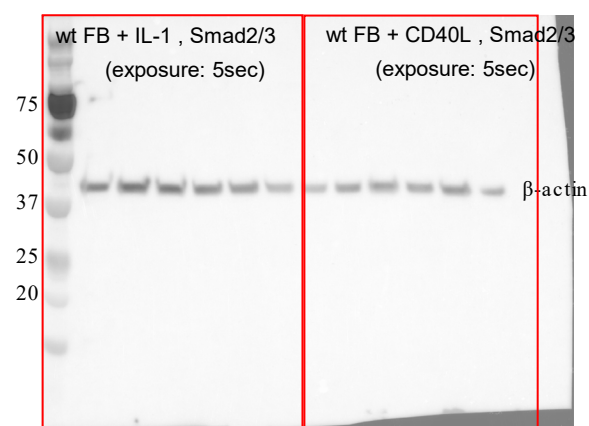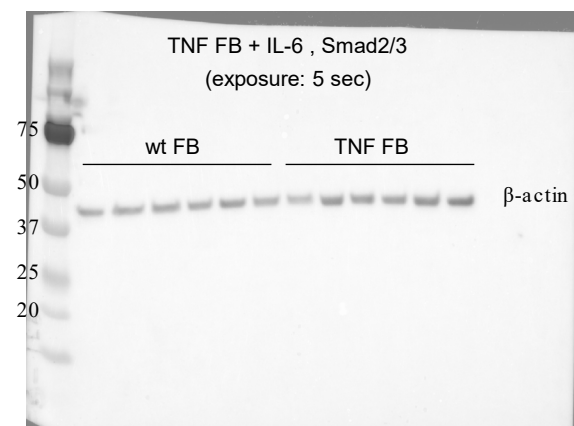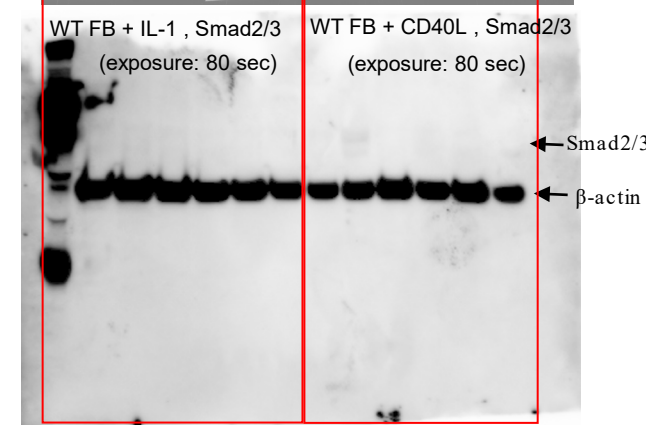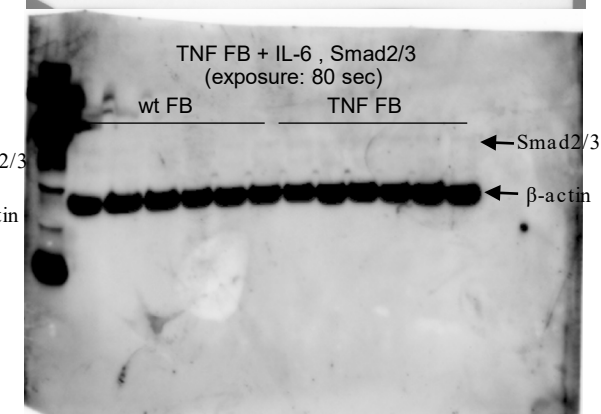

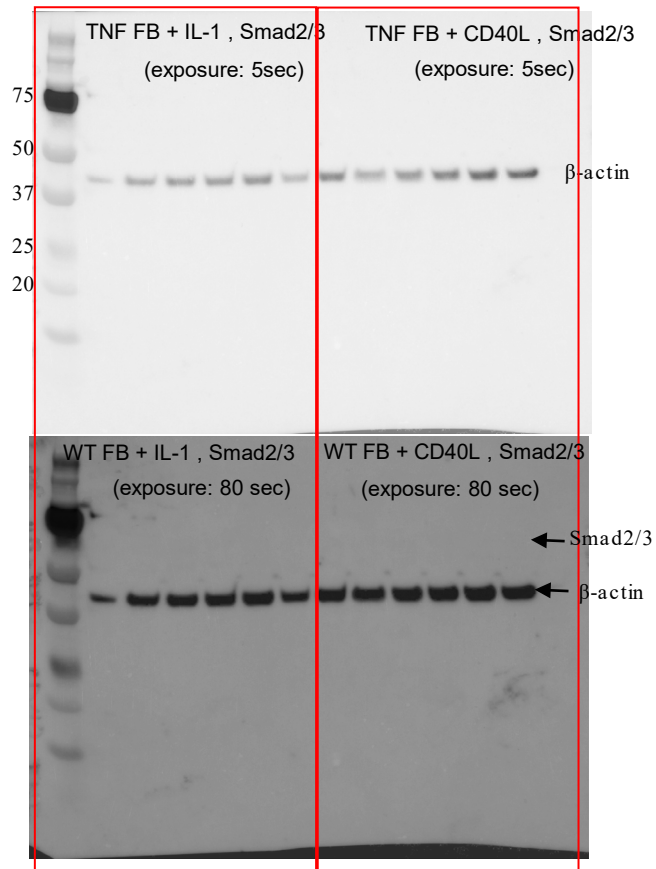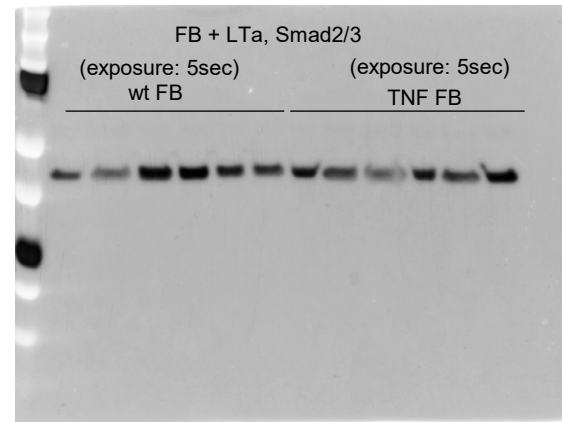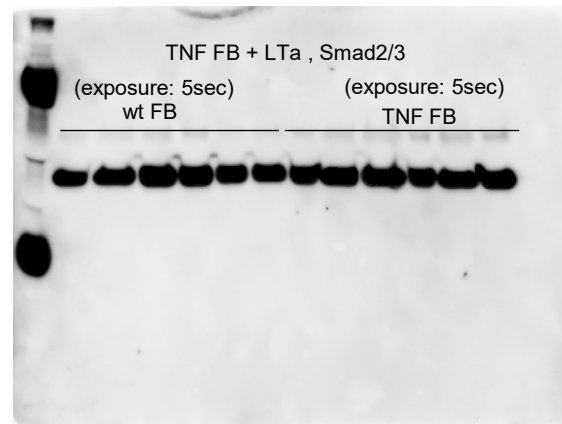

Smad2/3 = 60 Kda  
Smad1 = 60 KDa

Supplement: Unedited blot and gel images [file jciinsight-10-174456-s134.pdf]
